# Potential causal relationships between blood metabolites, inflammatory cytokines, and venous thromboembolism

**DOI:** 10.3389/fimmu.2024.1445790

**Published:** 2024-09-30

**Authors:** Qianying Liu, Fan Yang, Kangli Kong, Fangfang Lu

**Affiliations:** Department of Clinical Laboratory, Nanjing Jiangning Hospital, The Affiliated Jiangning Hospital with Nanjing Medical University, Nanjing, China

**Keywords:** metabolites, inflammatory cytokines, venous thromboembolism, Mendelian randomization analysis, deep vein thrombosis, pulmonary embolism

## Abstract

**Background:**

Venous thromboembolism (VTE) is the abnormal coagulation of blood in deep veins, which impairs venous return and includes deep vein thrombosis (DVT) and pulmonary embolism (PE). The incidence of VTE is increasing, leading to severe complications and sequelae. Despite the widespread application of multi-omics analyses in vascular disease research, identifying the specific links between various metabolic products, cytokines, and VTE, as well as their potential mediating roles, requires further validation due to confounding factors.

**Methods:**

Summary statistics for 1,091 metabolites, 309 metabolite ratios (8,299 individuals), and 41 inflammatory cytokines (8,293 individuals) were obtained from the largest genome-wide association studies (GWAS). Summary statistics for VTE (21,021 cases, 391,160 controls), DVT (6,501 cases, 357,111 controls), and PE (10,046 cases, 401,128 controls) were derived from the FinnGen R10 dataset. We initially examined causal relationships using two-sample MR analysis, followed by Two-step Mendelian Randomization (TSMR) and Multivariable Mendelian Randomization (MVMR) to identify potential mediating mechanisms.

**Results:**

We identified causal associations for 78 blood metabolites with VTE, 79 with DVT, and 81 with PE. Among all 41 inflammatory cytokines included, only platelet-derived growth factor BB (PDGF-BB) levels showed a causal relationship with increased risks of VTE, DVT, and PE. MVMR analysis revealed that the associations between glycocholate levels and VTE, DVT, and PE were mediated by PDGF-BB, accounting for 14.54% (p=2.84E-04), 17.10% (p=3.64E-05), and 10.44% (p=1.39E-02), respectively. Furthermore, the associations between dodecanedioate (C12:1-DC) levels and VTE and DVT were also mediated by PDGF-BB, accounting for 12.79% (p=6.10E-04) and 12.17% (p=2.13E-04), respectively.

**Conclusion:**

This study reveals significant associations between specific blood metabolites and the risks of VTE, DVT, and PE, with some associations potentially mediated by PDGF-BB.

## Introduction

Venous thromboembolism (VTE), which includes deep vein thrombosis (DVT) and pulmonary embolism (PE), is a serious health issue affecting approximately 10 million people globally each year (1). Currently, VTE diagnosis relies on clinical judgment and ultrasound examinations, with D-dimer testing widely used for its sensitivity, though it lacks specificity (2). Existing antithrombotic treatments primarily target the coagulation system and platelets, but their long-term use significantly increases bleeding risks. Therefore, identifying new biomarkers and potential causal risk factors for VTE is crucial to improve diagnostic and treatment strategies.

Metabolomics studies have demonstrated that metabolites potentially associated with VTE include carnitine, glucose, phenylalanine, 3-hydroxybutyrate, lactate, tryptophan, various monounsaturated and polyunsaturated fatty acids, mainly involves major metabolic pathways such as carbohydrate metabolism, lipid metabolism, amino acid metabolism, and energy metabolism (3, 4). In a case-control study conducted by Jiang and colleagues (5), which included 240 cases and 6,963 control subjects, the results showed a significant association between C5 carnitine and the occurrence of acute VTE. However, the characteristics of metabolomic profiles are often influenced by factors such as age, gender, smoking status, BMI, experimental techniques, and various environmental conditions. Furthermore, observational studies have typically involved a limited range of metabolites and small sample sizes, making the results susceptible to various confounding factors and reverse causality. Consequently, the causal relationship between circulating metabolites and the risk of VTE remains unclear.

Inflammatory factors are thought to play a significant role in VTE. Some inflammatory cytokines, such as tumor necrosis factor-alpha (TNF-α), interleukin-6 (IL-6), interleukin-8, and other inflammatory markers, have been extensively studied as predictive diagnostic tools for VTE (6, 7). Studies have shown that certain lipid metabolites significantly affect the function of inflammatory cells and the levels of cytokines in circulation. For example, omega-3 fatty acids, particularly eicosapentaenoic acid (EPA) and docosahexaenoic acid (DHA), exhibit anti-inflammatory properties. These effects are mediated by inhibiting the production of several inflammatory cytokines (such as TNF-α, IL-1β, and IL-6) in human endothelial cells and monocytes upon endotoxin stimulation. In contrast, omega-6 fatty acids, mainly represented by arachidonic acid, serve as precursors to various pro-inflammatory mediators and play a key role in promoting inflammatory responses (8, 9). Therefore, we hypothesize that there may be causal relationships between blood metabolites, inflammatory cytokines, and VTE.

Mendelian Randomization (MR) is an epidemiological method based on genome-wide association studies (GWAS) data that uses single nucleotide polymorphisms (SNPs) as instrumental variables (IVs) to assess the causal relationships between genetic variants and target outcomes. Due to the random allocation of genes, this method is less susceptible to confounding variables and measurement errors, and it avoids biases from reverse causation, thereby enhancing the accuracy of causal inference (10, 11). Recently, some MR studies have been limited to investigating the effects of fatty acids or phospholipid metabolites, which are subsets of lipids, on VTE. This study conducts a comprehensive MR analysis to examine the causal relationships between blood metabolites (including eight super pathways), inflammatory cytokines, and VTE, while further examining whether inflammatory cytokines mediate the association between metabolites and VTE.

## Methods

### Study design

**Figure 1:**
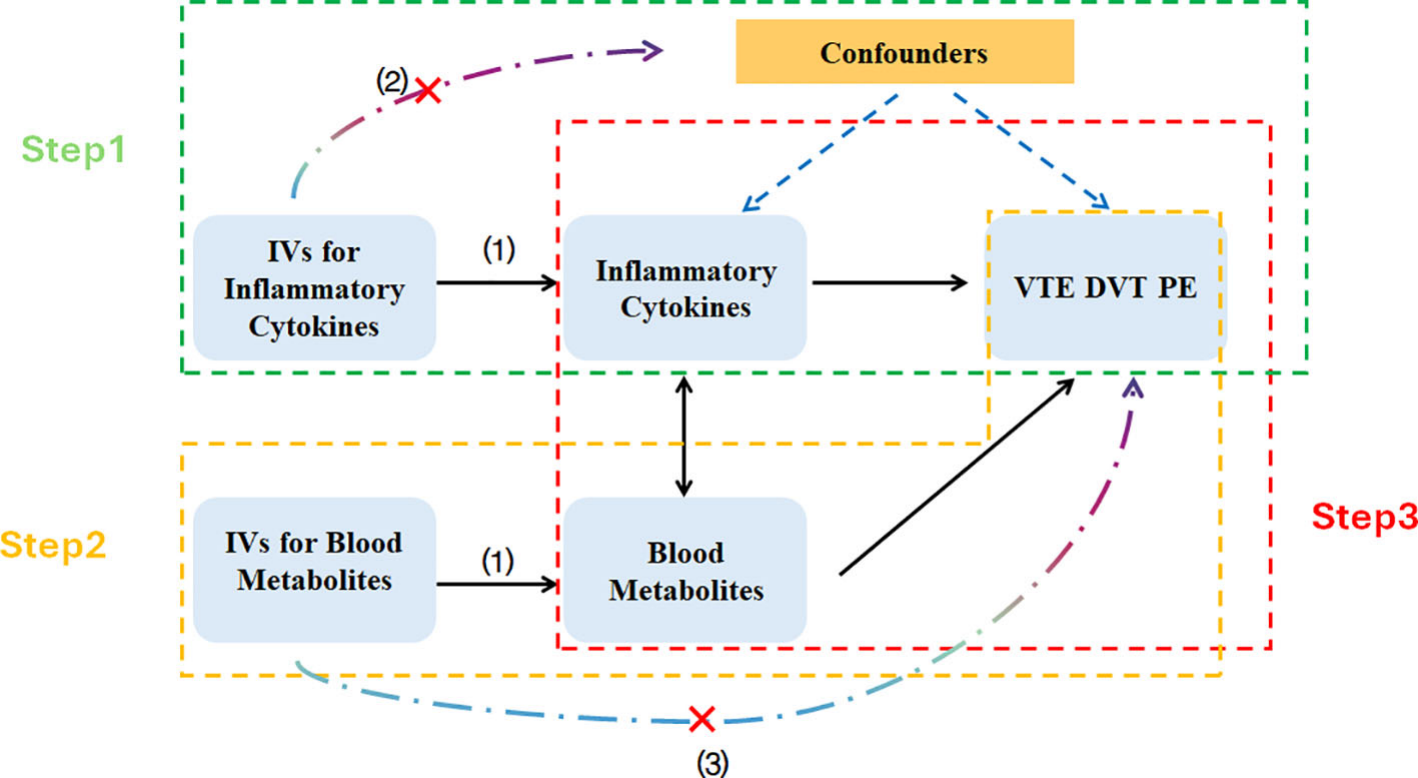
Schematic diagram of the Mendelian randomization analyses. IVs, Instrumental Variables; VTE, Venous Thromboembolism; DVT, Deep Vein Thrombosis; PE, Pulmonary Embolism.

The study comprises three main components: First, analyzing the causal impact of 1,091 blood metabolites and 309 metabolite ratios on VTE (including DVT and PE). Second, examining the causal effects of 41 inflammatory cytokines on VTE (including DVT and PE). Third, conducting mediation analysis using Two-step Mendelian Randomization (TSMR) and Multivariable Mendelian Randomization (MVMR). Before MR analysis, three fundamental assumptions must be addressed: (1) the relevance assumption, which requires genetic variants to exhibit strong and significant associations with the exposure variables; (2) the independence assumption, which mandates that IVs should be unrelated to any confounders; (3) the exclusion restriction assumption, which stipulates that genetic variants should affect the outcome solely through the exposure, without involvement in other pathways (**Figure 1**).

### Data source

For blood metabolites, we sourced GWAS data from research by Yiheng Chen et al., which covered 1,091 metabolites and 309 metabolite ratios, involving 8,299 participants from the Canadian Longitudinal Study on Aging (CLSA) cohort, Of the 1,091 plasma metabolites tested, 850 had known identities across eight super pathways (i.e., lipid, amino acid, xenobiotics, nucleotide, cofactor and vitamins, carbohydrate, peptide, and energy). The remaining 241 were categorized as unknown or “partially” characterized molecules (12). To identify SNPs associated with inflammatory cytokines, we utilized data from the study conducted by Ahola-Olli et al., which included analyses of 41 inflammatory cytokines in a cohort of 8,293 individuals (13). Summary statistics for VTE involved data from 21,021 cases and 391,160 controls. For DVT, the data included 6,501 cases and 357,111 controls; for PE, the figures were 10,046 cases and 401,128 controls. These statistics were sourced from the publicly released GWAS data of the FinnGen consortium (Release 10, available at https://www.finngen.fi/en). In this dataset, the diagnoses of VTE, DVT, and PE must conform to ICD-9 or ICD-10 standards, with the overall median ages of the samples being 60.20 years, 58.86 years, and 65.50 years, respectively. All GWAS summary data focused on European populations.

This study is a secondary analysis of publicly available GWAS summary statistics. Each original GWAS study received ethical approval. As this analysis did not involve individual-level data, no new ethical review board approval was necessary.

### Instrumental variable selection

For blood metabolites, SNPs were chosen with a threshold of p< 1 × 10^-5^ (14). To maximize the number of available SNPs for inflammatory cytokines, we selected those with a significance threshold of p< 5 × 10^-6^ (15). To minimize the impact of linkage disequilibrium (LD) on our analysis, we set threshold parameters at r^2^ = 0.001 and a distance criterion of 10,000 kb. To ensure a robust association between IVs and exposures, we calculated the explained variance (R^2^) and assessed the strength of each IV using the F-statistic, excluding any SNPs with an F-statistic below 10 (16). These calculations were based on several parameters: minor allele frequency (MAF), effect size on exposure (β), standard deviation (SD), sample size (N), and the number of IVs (k).


$$R^{2}=2\times \left(1-\text{MAF}\right)\times \text{MAF}\times \frac{\beta }{\text{SD}}$$

$$F=\frac{N-k-1}{k}\times \frac{R^{2}}{1-R^{2}}$$

To enhance the reliability of our analysis by minimizing biases and confounding, we excluded palindromic SNPs with intermediate allele frequencies that could interfere with genotype-phenotype associations and compromise result accuracy (17). Additionally, we utilized PhenoScanner (www.phenoscanner.medschl.cam.ac.uk) to verify if the exposure SNPs were linked to potential risk factors (18).

### Mendelian randomization analysis

Our study utilized five MR methods to investigate the causal relationships between blood metabolites, inflammatory cytokines, and VTE. These methods included the Inverse Variance Weighting (IVW), Median Weighted, MR-Egger, Weighted Mode, and Simple Mode. The IVW method, our primary analytical approach, if heterogeneity is present, utilize a random effects model. The IVW method assigns weights to each study’s effect estimate based on the inverse of its variance, thereby integrating these weighted estimates to produce a more precise composite effect estimate (19). Causal effects were assessed using Odds Ratios (OR) and their 95% Confidence Intervals (CI). To reduce the risk of false positives, multiple comparisons were adjusted using the Benjamini-Hochberg false discovery rate (FDR). An association is considered statistically significant if the FDR-adjusted p-value is less than 0.05. If the p-value is below 0.05 but higher than the FDR-adjusted p-value, the association is considered suggestive.

### Mediation analysis

In the mediation analysis, we included factors within the blood metabolite -inflammatory cytokine -VTE pathway that demonstrated significant causal effects (p<0.05). The total effect of metabolites on VTE, DVT, and PE was defined as β_EO_. Initially, we explored potential mediation effects through TSMR. The TSMR method assumes no interaction between the exposure and the mediator (20). In this process, we conducted a two-step analysis: the first step was to determine the causal effect of the exposure (metabolites) on the mediator (inflammatory cytokines), denoted as β_EM_; the second step was to determine the causal effect of the mediator on the outcome (VTE/DVT/PE), denoted as β_MO_. Subsequently, we employed MVMR for a more in-depth validation of these relationships to confirm the mediation effects revealed by TSMR. MVMR also assumes no interaction between the exposure and the mediator and is particularly well-suited for studying causal relationships among multiple related exposures (21). In MVMR analysis, the effect of exposure on the outcome after adjusting for the mediator is defined as the direct effect β'_EO_ the effect of the mediator on the outcome after adjusting for exposure is defined as the direct effect β'_MO_ and the causal effect of exposure on the outcome via the mediator is defined as the mediated effect β_EMO_. The formula for calculating the mediated effect is: β_EMO_= β_EM_*β ' _MO_; The formula for calculating the standard error is: $SE_{EMO}=\sqrt{{\left(\beta {'}_{MO}\right)}^{2}*{\left(SE_{EM}\right)}^{2}+{\left(\beta_{EM}\right)}^{2}*{\left(SE{'}_{MO}\right)}^{2}}$ ; The formula for calculating the mediation proportion is: β_EMO/_β_EO_.

### Sensitivity analysis

For the sensitivity analysis, Cochran’s Q test was utilized to evaluate heterogeneity among individual SNPs. A p-value greater than 0.05 indicates no significant heterogeneity. The Mendelian Randomization Pleiotropy Residual Sum and Outlier (MR-PRESSO) test was employed to assess the presence of horizontal pleiotropy. MR-PRESSO not only automatically detects outliers in the IVW linear regression but also removes anomalous SNPs and provides corrected estimates to address the effects of horizontal pleiotropy (22). Additionally, a “leave-one-out” sensitivity analysis was conducted to ascertain whether the results were influenced by any individual SNP (19). All analyses in this study were performed using the “TwoSampleMR” package in R version 4.3.1.

## Results

### Selection of instrumental variables

In the analysis of the 1,091 metabolites and 309 metabolite ratios assessed, the number of SNPs used varied from 7 to 73, with F-statistics from 19.50 to 5308.35 (**Supplementary Table 1**). For the 41 inflammatory cytokines, the number of SNPs used as IVs ranged from 4 to 17, with F-statistics between 20.77 and 782.26 (**Supplementary Table 8**). All SNPs involved had F-statistics greater than 10, suggesting that our results are robust and unlikely to be affected by biases due to weak IVs.

### Causal relationships between blood metabolites and VTE

**Figure 2:**
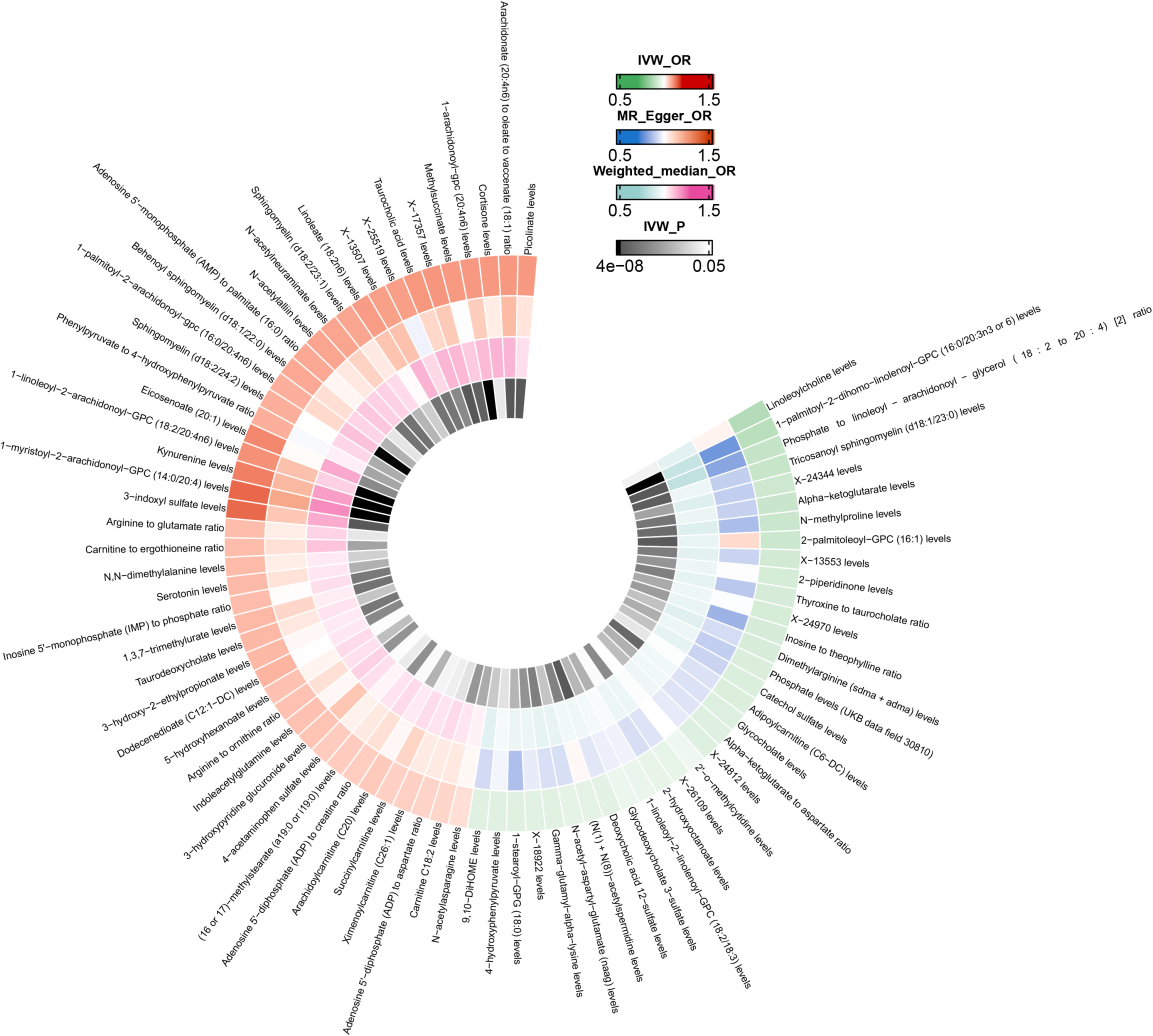
Mendelian randomization analysis of the causal effects of blood metabolites on venous thromboembolism. This figure presents the Odds Ratios estimated using three distinct Mendelian randomization methods: Inverse Variance Weighted, MR-Egger, and Weighted Median. Results have been truncated at P-Value< 0.05 following Inverse Variance Weighted method. IVW, Inverse Variance Weighted; OR, Odds Ratio; P, P-Value; VTE, Venous Thromboembolism.

**Figure 3:**
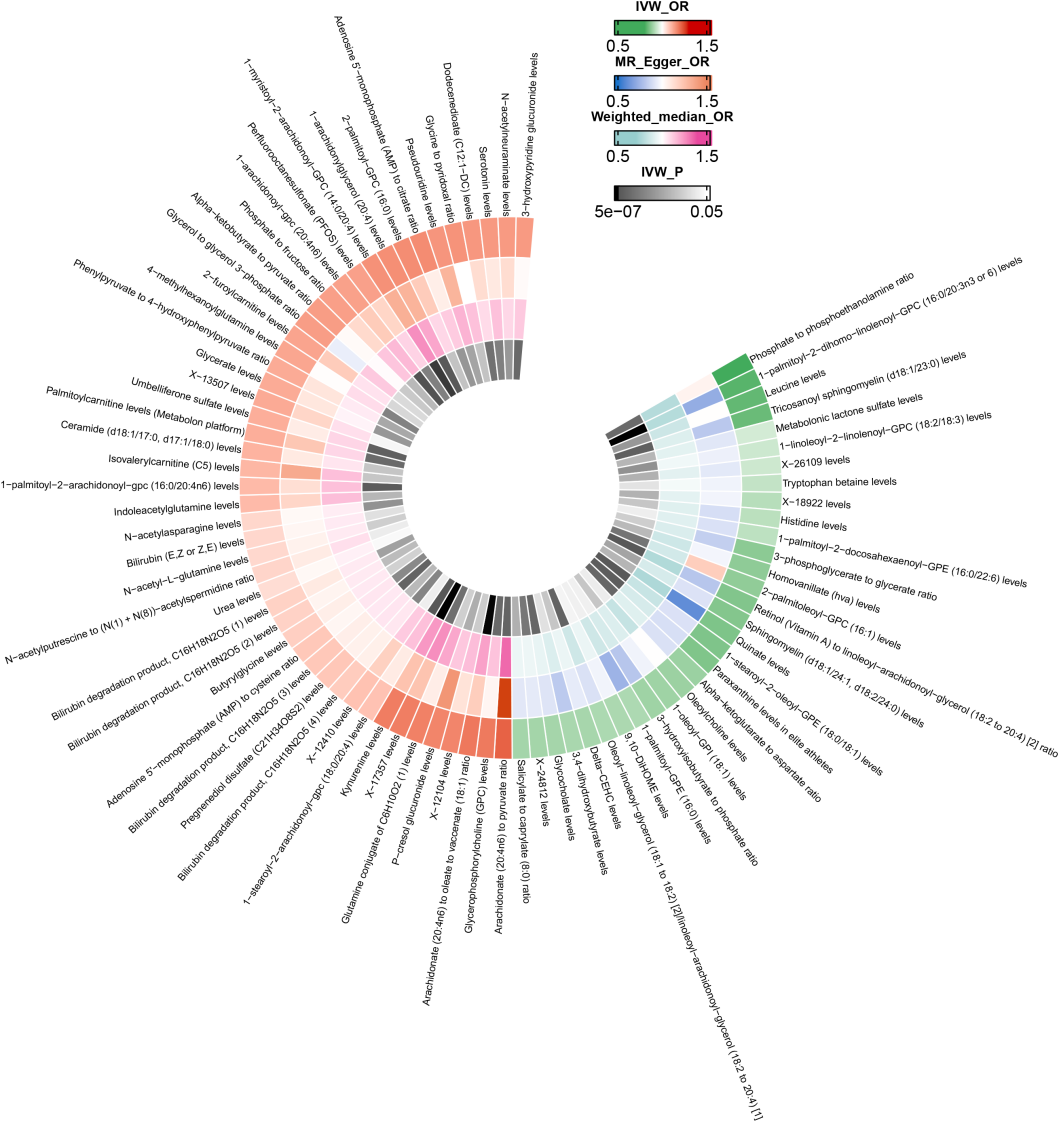
Mendelian randomization analysis of the causal effects of blood metabolites on deep vein thrombosis. This figure presents the Odds Ratios estimated using three distinct Mendelian randomization methods: Inverse Variance Weighted, MR-Egger, and Weighted Median. Results have been truncated at P-Value< 0.05 following Inverse Variance Weighted method. IVW, Inverse Variance Weighted; OR, Odds Ratio; P, P-Value; DVT, Deep Vein Thrombosis.

**Figure 4:**
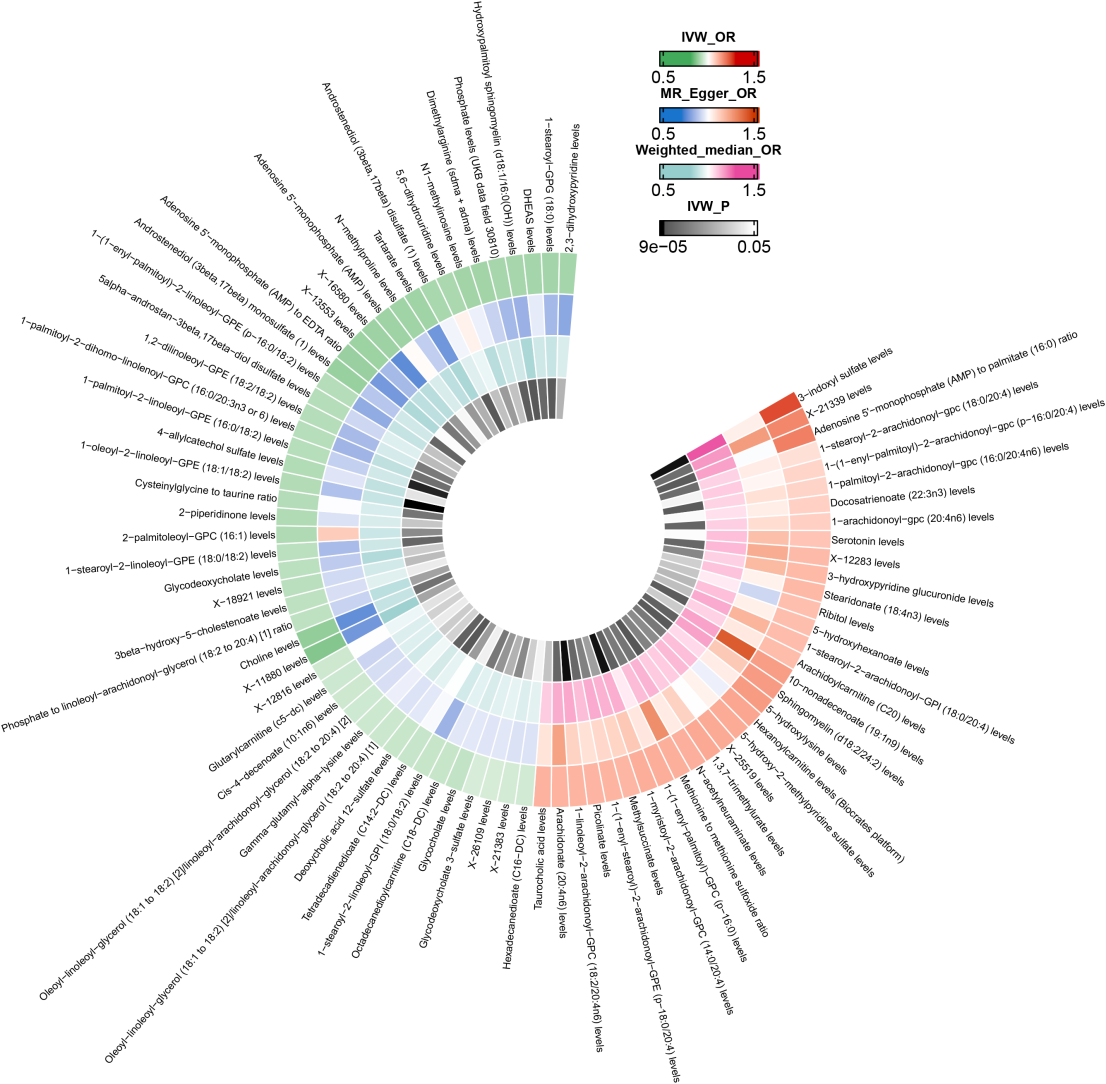
Mendelian randomization analysis of the causal effects of blood metabolites on pulmonary embolism. This figure presents the Odds Ratios estimated using three distinct Mendelian randomization methods: Inverse Variance Weighted, MR-Egger, and Weighted Median. Results have been truncated at P-Value< 0.05 following Inverse Variance Weighted method. IVW, Inverse Variance Weighted; OR, Odds Ratio; P, P-Value; PE, Pulmonary Embolism.

After excluding SNPs closely associated with BMI and smoking, the MR analysis results for the causal impact of 1,091 metabolites and 309 metabolite ratios on VTE (including DVT and PE) are shown in **Supplementary Tables 2–4**. To avoid the influence of pleiotropic SNPs on MR analysis results, metabolites that no longer had a causal association with outcomes (p>0.05) after MR-PRESSO outlier correction were excluded. Initial IVW results indicated that 78 metabolites were associated with VTE, 79 with DVT, and 81 with PE (**Figures 2**–**4** and **Supplementary Tables 5–7**). After FDR correction, 9 metabolites remained significantly associated with VTE risk, 5 with DVT, but no significant associations were found with PE (**Table 1**). Among these, only the levels of 1-palmitoyl-2-dihomo-linolenoyl-GPC (16:0/20:3n3 or 6) were negatively correlated with the risks of VTE (OR: 0.882; 95%CI: 0.839-0.928; p=9.61E-07) and DVT (OR: 0.820; 95%CI: 0.759-0.886; p=5.19E-07), while others showed a positive correlation with the risk of VTE and DVT. Cochran’s Q test indicated no significant heterogeneity in these associations (p > 0.05), and MR-PRESSO did not identify any outliers. Leave-one-out analysis, scatter plots, funnel plots, and forest plots of the MR analysis for the association between relevant metabolites and VTE and DVT are shown in **Supplementary Figures 2–15**.

**Table 1:** Mendelian randomization analysis of the causal effects of blood metabolites on venous thromboembolism, deep vein thrombosis and pulmonary embolism after P-value adjusted for false discovery rate.

Exposures	Metabolic Pathway	Outcomes	Method	nSNP	Beta	SE	P	OR (95% CI)	P_FDR	Heterogeneity (MR-Egger)	Heterogeneity (IVW)	Pleiotropy (MR-PRESSO)
1-myristoyl-2-arachidonoyl-GPC (14:0/20:4) levels	Phosphatidylcholine	VTE	Inverse variance weighted	22	0.132	0.024	4.82E-08	1.141 (1.088, 1.196)	6.74E-05	0.341	0.127	0.110
1-arachidonoyl-gpc (20:4n6) levels	Lysophospholipid		Inverse variance weighted	20	0.092	0.018	2.49E-07	1.096 (1.059, 1.135)	1.75E-04	0.272	0.161	0.215
Kynurenine levels	Tryptophan Metabolism		Inverse variance weighted	23	0.114	0.023	8.11E-07	1.120 (1.071, 1.172)	3.78E-04	0.197	0.095	0.099
1-palmitoyl-2-dihomo-linolenoyl-GPC (16:0/20:3n3 or 6) levels	Phosphatidylcholine		Inverse variance weighted	21	-0.125	0.026	9.61E-07	0.882 (0.839, 0.928)	3.36E-04	0.844	0.176	0.109
1-linoleoyl-2-arachidonoyl-GPC (18:2/20:4n6) levels	Phosphatidylcholine		Inverse variance weighted	23	0.099	0.021	1.83E-06	1.104 (1.060, 1.150)	5.13E-04	0.353	0.249	0.103
1-palmitoyl-2-arachidonoyl-gpc (16:0/20:4n6) levels	Phosphatidylcholine		Inverse variance weighted	26	0.073	0.016	4.38E-06	1.076 (1.043, 1.111)	1.02E-03	0.120	0.105	0.127
X-17357 levels	Unknown		Inverse variance weighted	25	0.095	0.027	4.17E-04	1.099 (1.043, 1.158)	4.49E-02	0.461	0.501	0.522
Arachidonate (20:4n6) to oleate to vaccenate (18:1) ratio			Inverse variance weighted	19	0.092	0.026	4.40E-04	1.097 (1.042, 1.155)	4.40E-02	0.071	0.011	0.082
3-indoxyl sulfate levels	Tryptophan Metabolism		Inverse variance weighted	15	0.132	0.038	5.47E-04	1.141 (1.059, 1.229)	4.79E-02	0.359	0.434	0.469
1-arachidonoyl-gpc (20:4n6) levels	Lysophospholipid	DVT	Inverse variance weighted	20	0.126	0.032	9.59E-05	1.134 (1.064, 1.208)	2.69E-02	0.290	0.116	0.201
1-palmitoyl-2-dihomo-linolenoyl-GPC (16:0/20:3n3 or 6) levels	Phosphatidylcholine		Inverse variance weighted	21	-0.199	0.040	5.19E-07	0.820 (0.759, 0.886)	7.27E-04	0.995	0.931	0.833
1-myristoyl-2-arachidonoyl-GPC (14:0/20:4) levels	Phosphatidylcholine		Inverse variance weighted	22	0.145	0.036	6.67E-05	1.156 (1.076, 1.241)	2.33E-02	0.931	0.574	0.318
Arachidonate (20:4n6) to oleate to vaccenate (18:1) ratio			Inverse variance weighted	19	0.174	0.038	4.17E-06	1.190 (1.105, 1.281)	1.94E-03	0.146	0.184	0.248
Kynurenine levels	Tryptophan Metabolism		Inverse variance weighted	23	0.168	0.034	9.98E-07	1.183 (1.106, 1.265)	6.99E-04	0.466	0.434	0.402

VTE, Venous Thromboembolism; DVT, Deep Vein Thrombosis; nSNP, the number of Single Nucleotide Polymorphisms; SE, Standard Error; P, P-Value; OR, Odds Ratio; CI, Confidence Interval; P_FDR, P-value adjusted for False Discovery Rate; IVW, Inverse Variance Weighted; MR-PRESSO, Mendelian Randomization Pleiotropy RESidual Sum and Outlier.

### Causal relationships between inflammatory cytokines and VTE

**Figure 5:**
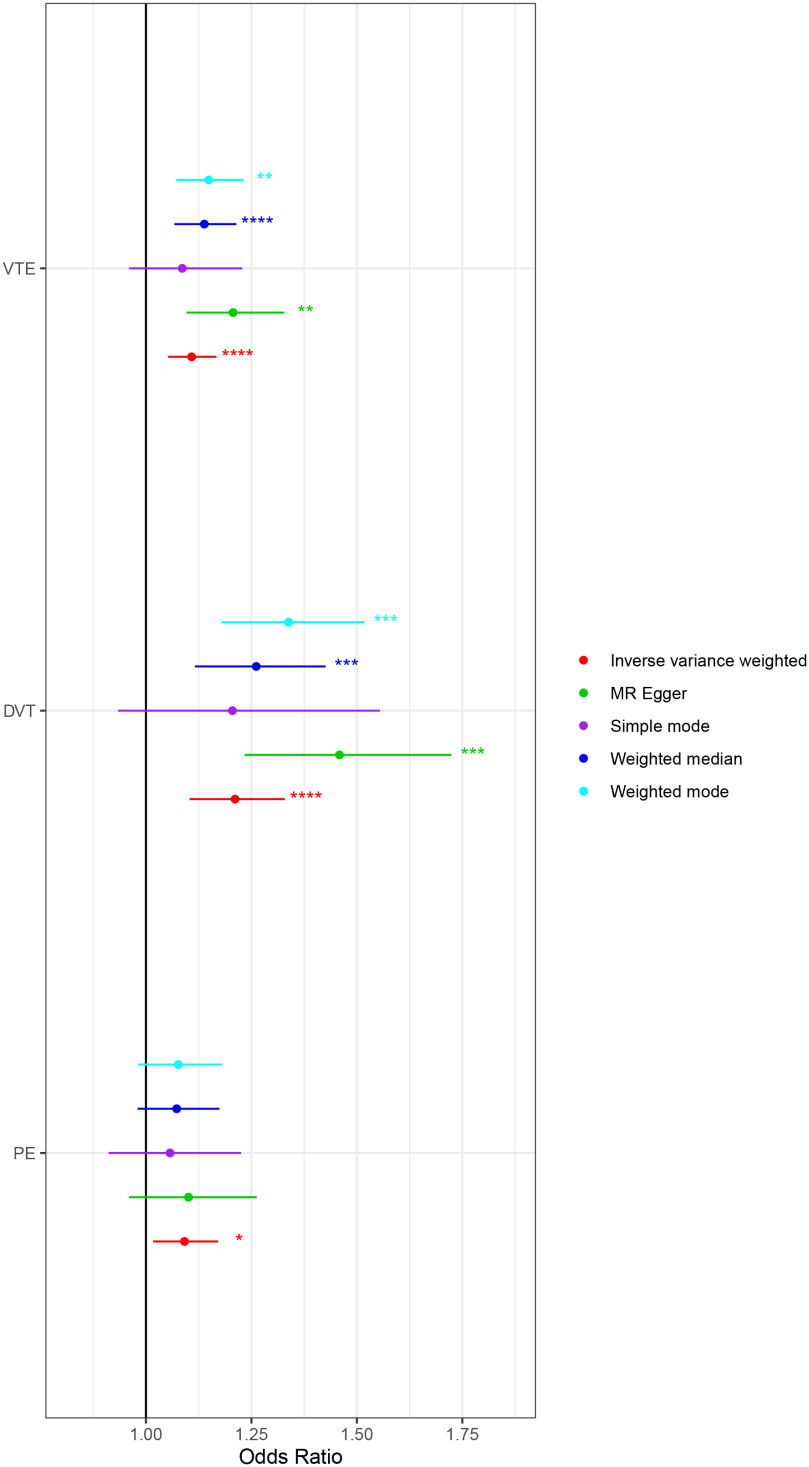
Mendelian randomization analysis of the causal effects of PDGF-BB on venous thromboembolism, deep vein thrombosis and pulmonary embolism. VTE, Venous Thromboembolism; DVT, Deep Vein Thrombosis; PE, Pulmonary Embolism. ‘*’ indicates P-value ≤ 0.05, ‘**’ indicates P-value ≤ 0.01, ‘***’ indicates P-value ≤ 0.001, ‘****’ indicates P-value ≤ 0.0001.

The MR analysis evaluating the effect of 41 inflammatory cytokines on VTE (including DVT and PE) is detailed in **Supplementary Figure 1** and **Supplementary Tables 9–11**. Utilizing the IVW method, elevated PDGF-BB levels were positively correlated with increased risks for VTE (OR: 1.108; 95% CI: 1.053-1.167; p=9.29E-05), DVT (OR: 1.211; 95% CI: 1.103-1.330; p=5.66E-05), and PE (OR: 1.091; 95% CI: 1.017-1.171; p=0.015). After adjustment for FDR, the p-value for VTE and DVT were revised to 0.004 and 0.002, respectively, indicating significant associations. However, the FDR q-value for PE was adjusted to 0.627, signifying a lack of statistical significance, all the results are presented in **Table 2**. Cochran’s Q test indicated no significant heterogeneity among these associations (p > 0.05), and MR-PRESSO identified no outliers. The findings from five distinct MR methods investigating the causal relationships between PDGF-BB and VTE (including DVT and PE) are illustrated in **Figure 5**. Further analyses, including leave-one-out, scatter plots, funnel plots, and forest plots, are presented in **Supplementary Figures 16–18**.

**Table 2:** The impact of inflammatory cytokines on venous thromboembolism, deep vein thrombosis, and pulmonary embolism in Mendelian randomization analyses (Cutoff at p-value<0.05).

Exposure	Outcome	Method	nSNP	Beta	SE	P	OR (95% CI)	P_FDR	Heterogeneity (MR-Egger)	Heterogeneity (IVW)	Pleiotropy (MR-PRESSO)
PDGF-BB	VTE	Inverse variance weighted	12	0.103	0.026	9.29E-05	1.108 (1.053-1.167)	0.004	0.645	0.369	0.338
PDGF-BB	DVT	Inverse variance weighted	12	0.192	0.048	5.66E-05	1.211 (1.103-1.330)	0.002	0.788	0.308	0.222
PDGF-BB	PE	Inverse variance weighted	12	0.087	0.036	0.015	1.091 (1.017-1.171)	0.627	0.652	0.732	0.792

PDGF-BB, Platelet-Derived Growth Factor BB; VTE, Venous Thromboembolism; DVT, Deep Vein Thrombosis; PE, Pulmonary Embolism; nSNP, the number of Single Nucleotide Polymorphisms; SE, Standard Error; P, P-Value; OR, Odds Ratio; CI, Confidence Interval; P_FDR, P-value adjusted for False Discovery Rate; IVW, Inverse Variance Weighted; MR-PRESSO, Mendelian Randomization Pleiotropy RESidual Sum and Outlier.

### Mediation analysis

To investigate whether PDGF-BB plays a mediating role in the associations between metabolites and VTE (including DVT and PE), 78 metabolites associated with VTE, 79 with DVT, and 81 with PE were used as exposure factors to explore their causal relationships with PDGF-BB. The results of the MR analysis are presented in **Supplementary Tables 14–16**. In the TSMR Analysis, the IVW results show that glycocholate levels mediate the association through PDGF-BB with VTE, as well as DVT and PE; dodecanedioate (C12:1-DC) levels mediate the association through PDGF-BB with VTE and DVT (**Table 3**). Further MVMR analysis reveals that after adjusting for glycocholate levels, the mediating role of PDGF-BB in the causal associations between glycocholate levels and VTE, DVT, and PE remains significant. The mediation effect for VTE is -0.009 (95%CI: -0.016, -0.001; p=2.84E-04), representing 14.538% of the mediation proportion; for DVT, it is -0.018 (95%CI: -0.034, -0.002; p=3.64E-05), representing 17.101% of the mediation proportion; for PE, it is -0.007 (95%CI: -0.015, 0.001; p=1.39E-02), representing 10.437% of the mediation proportion. After adjusting for dodecanedioate (C12:1-DC) levels, PDGF-BB’s mediating role in the causal associations between dodecanedioate levels and VTE and DVT remains significant. The mediation effect for VTE is 0.009 (95%CI: -0.001, 0.018; p=6.10E-04), representing 12.787% of the mediation proportion; for DVT, it is 0.017 (95%CI: -0.002, 0.035; p=2.13E-04), representing 12.170% of the mediation proportion (**Table 4**).

**Table 3:** Two-step Mendelian randomization analyses of the causal effects between blood metabolites, inflammatory cytokines on venous thromboembolism, including deep vein thrombosis and pulmonary embolism.

Exposures	Mediator	Outcomes	Exposure to Mediator			Mediator to Outcome			Exposure to Outcome		
			β_EM_(95%CI)	SE_EM_	P_EM_	β_MO_ (95%CI)	SE_MO_	P_MO_	β_EO_ (95%CI)	SE_EO_	P_EO_
Glycocholate levels	PDGF-BB	VTE	-0.097 (-0.168, -0.026)	0.036	7.44E-03	0.103 (0.051, 0.154)	0.026	9.29E-05	-0.059 (-0.101, -0.017)	0.021	5.60E-03
Dodecanedioate (C12:1-DC) levels	PDGF-BB	VTE	0.096 (0.002, 0.189)	0.048	4.49E-02	0.103 (0.051, 0.154)	0.026	9.29E-05	0.068 (0.013, 0.123)	0.028	1.61E-02
Glycocholate levels	PDGF-BB	DVT	-0.097 (-0.168, -0.026)	0.036	7.44E-03	0.192 (0.098, 0.285)	0.048	5.66E-05	-0.106 (-0.180, -0.032)	0.038	4.90E-03
Dodecanedioate (C12:1-DC) levels	PDGF-BB	DVT	0.096 (0.002, 0.189)	0.048	4.49E-02	0.192 (0.098, 0.285)	0.048	5.66E-05	0.137 (0.035, 0.240)	0.052	8.60E-03
Glycocholate levels	PDGF-BB	PE	-0.097 (-0.168, -0.026)	0.036	7.44E-03	0.087 (0.017, 0.158)	0.036	1.53E-02	-0.071 (-0.130, -0.011)	0.030	2.03E-02

PDGF-BB, Platelet-Derived Growth Factor BB; GWAS, Genome-Wide Association Study; VTE, Venous Thromboembolism; DVT, Deep Vein Thrombosis; PE, Pulmonary Embolism; CI, Confidence Interval; β_EM_, The effect of exposure on mediator; SE_EM_, The standard error of exposure on mediator; P_EM_, The P-Value of exposure on mediator; β_MO_, The effect of the mediator on the outcome; SE_MO_, The standard error of mediator on outcome; P_MO_, The P-Value of mediator on outcome; β_EO_, The effect of the exposure on the outcome; SE_EO_, The standard error of exposure on outcome; P_EO_, The P-Value of exposure on outcome.

**Table 4:** Multivariable Mendelian randomization analyses of the causal effects between blood metabolites, inflammatory cytokines on venous thromboembolism, including deep vein thrombosis and pulmonary embolism.

Exposures	Mediator	Outcomes	Direct effect			Total effect	Mediation effect			Mediated proportion	Mediated proportion (%)
			β_EM_ (95%CI)	β’_MO_ (95%CI)	β’_EO_ (95%CI)	β_EO_ (95%CI)	β_EMO_ (95%CI)	SE_EMO_	P_EMO_		
Glycocholate levels	PDGF-BB	VTE	-0.097 (-0.168, -0.026)	0.089 (0.041, 0.136)	-0.026 (-0.069, 0.017)	-0.059 (-0.101, -0.017)	-0.009 (-0.016, -0.001)	0.004	2.84E-04	0.145	14.538
Dodecanedioate (C12:1-DC) levels	PDGF-BB	VTE	0.096 (0.002, 0.189)	0.090 (0.039, 0.142)	-0.019 (-0.080, 0.042)	0.068 (0.013, 0.123)	0.009 (-0.001, 0.018)	0.005	6.10E-04	0.128	12.787
Glycocholate levels	PDGF-BB	DVT	-0.097 (-0.168, -0.026)	0.187 (0.098, 0.276)	0.003 (-0.078, 0.083)	-0.106 (-0.180, -0.032)	-0.018 (-0.034, -0.002)	0.008	3.64E-05	0.171	17.101
Dodecanedioate (C12:1-DC) levels	PDGF-BB	DVT	0.096 (0.002, 0.189)	0.174 (0.082, 0.266)	-0.008 (-0.118, 0.102)	0.137 (0.035, 0.240)	0.017 (-0.002, 0.035)	0.009	2.13E-04	0.122	12.170
Glycocholate levels	PDGF-BB	PE	-0.097 (-0.168, -0.026)	0.076 (0.015, 0.136)	-0.048 (-0.102, 0.006)	-0.071 (-0.130, -0.011)	-0.007 (-0.015, 0.001)	0.004	1.39E-02	0.104	10.437

PDGF-BB, Platelet-Derived Growth Factor BB; GWAS, Genome-Wide Association Study; VTE, Venous Thromboembolism; DVT, Deep Vein Thrombosis; PE, Pulmonary Embolism; CI, Confidence Interval; β_EM_, The effect of exposure on mediator; β’_MO_, The effect of mediator on the outcome after adjusting for the exposure; β’_EO_, The effect of exposure on the outcome after adjusting for the mediator; β_EO_, The total effect of the exposure on the outcome; β_EMO_, The effect of the exposure on the outcome through the mediator, representing the mediation effect; SE_EMO_, The standard error of the exposure on the outcome through the mediator; P_EMO_, The P-Value of the exposure on the outcome through the mediator. β_EMO_= β_EM_*β’_MO_; $SE_{EMO}=\sqrt{{\left(\beta {'}_{MO}\right)}^{2}*{\left(SE_{EM}\right)}^{2}+{\left(\beta_{EM}\right)}^{2}*{\left(SE{'}_{MO}\right)}^{2}}$ ; 95%CI: β_EMO_ **±** 1.96*SE_EMO_; Mediated proportion: β_EMO/_β_EO_; Mediated proportion (%): (β_EMO/_β_EO_) *100%.

## Discussion

Traditional observational studies often face limitations in establishing causal relationships due to confounding factors and reverse causation. While randomized controlled trials (RCTs) are considered the gold standard for validating epidemiological hypotheses, their strict design and high costs often limit their feasibility (23). In recent years, MR studies have emerged as a valuable alternative to RCTs by leveraging the randomness of genetic variations and the independence of IVs to enhance causal inference accuracy (24). Using MR studies, we identified 78, 79, and 81 blood metabolites associated with the risks of VTE, DVT, and PE, respectively. Among these, 9 metabolites exhibited significant associations with VTE risk, while 5 metabolites showed significant associations with DVT risk. These associations primarily involved the phosphatidylcholine and tryptophan metabolism pathways. However, no significant associations were found between blood metabolites and PE risk. Among the 41 inflammatory cytokines analyzed, circulating levels of PDGF-BB were significantly positively correlated with the risks of VTE and DVT and suggestively positively correlated with the risk of PE. Further mechanistic exploration suggested that glycocholate levels might reduce the risks of VTE, DVT, and PE by decreasing circulating PDGF-BB levels, whereas dodecanedioate (C12:1-DC) levels might increase the risks of VTE and DVT by elevating circulating PDGF-BB levels.

Our research indicates that elevated levels of certain arachidonic acid-containing phosphatidylcholines are associated with an increased risk of VTE and its subtypes. In contrast, higher levels of metabolites containing linolenic acid residues are associated with a reduced risk of VTE and its subtypes. We infer that this is mainly due to differences in the length of their fatty acid chains, the number of double bonds, and their positions. The former contains arachidonic acid (20:4), a representative member of omega-6 fatty acids, which may increase the risk of thrombosis through pro-inflammatory and platelet aggregation-promoting effects. The latter contains dihomo-γ-linolenic acid (20:3), a member of the omega-3 fatty acids, which may reduce the risk of thrombosis through anti-inflammatory and platelet aggregation-inhibiting effects (25, 26). A recent MR analysis indicated that phosphatidylcholine acyl-alkyl C40:4 (PC ae C40:4) was negatively associated with VTE, whereas phosphatidylcholine diacyl C42:6 (PC aa C42:6) and phosphatidylcholine acyl-alkyl C36:4 (PC ae C36:4) were positively associated with PE. The causal relationship of phosphatidylcholine with different carbon chain lengths and numbers of double bonds on VTE and PE may vary in both positive and negative directions (27). Studies have shown that genetically predicted increased levels of alpha-linolenic acid (ALA) and linoleic acid (LA), as well as decreased levels of arachidonic acid, are associated with a reduced risk of VTE and DVT. Additionally, arachidonic acid is considered an independent marker for VTE (28, 29). Similarly, we have found that when these fatty acids are attached to larger molecular structures, they sometimes exhibit characteristics similar to the complete fatty acid molecules. Our analysis indicates that genetically predicted levels of kynurenine and 3-indoxyl sulfate, both metabolites in the tryptophan metabolism pathway, are significantly associated with an increased risk of VTE. A study found that elevated levels of kynurenine and indoxyl sulfate in murine blood are associated with an increased risk of cancer associated VTE. These tryptophan metabolites may function as ligands for the aryl hydrocarbon receptor (AHR), leading to the activation of the AHR-TF/PAI-1 axis, which upregulates the expression of tissue factor (TF) and plasminogen activator inhibitor-1 (PAI-1), thereby promoting thrombus formation and stabilization (30).

To explore whether inflammatory cytokines mediate the relationship between blood metabolites and VTE, we selected 41 effective genetic variations of inflammatory cytokines and used GWAS to investigate their causal relationships with VTE, DVT, and PE. Among all the inflammatory cytokines included, PDGF-BB was the only one found to have a causal relationship with VTE, DVT, and PE. Bruzelius et al. (31)conducted a high-throughput affinity plasma proteomics screening of approximately 400 proteins associated with VTE risk in 88 patients and 85 healthy controls from Sweden, identifying a significant association between the occurrence of VTE and plasma levels of PDGF-BB. Their findings were subsequently validated in an independent French sample repository (named FARIVE) comprising 580 cases and 589 controls. In a case-control study involving 40 DVT patients, researchers found significantly elevated PDGF-BB expression in both acute and chronic DVT patients compared to healthy controls. Furthermore, PDGF-BB exhibited higher specificity in detecting DVT compared to traditional D-dimer testing (32). Hu et al. (33) recently employed MR to investigate the causal relationships between 41 inflammatory cytokines and VTE. Their analysis indicated a suggestive association between stromal cell-derived factor-1α (SDF-1α) and a reduced risk of VTE and DVT, as well as a suggestive association between granulocyte colony-stimulating factor (G-CSF) and PE. While these suggestive associations demonstrate some level of statistical significance, they do not completely rule out the possibility of false positives. Therefore, larger-scale or more rigorous follow-up studies are needed to validate these findings. Likewise, we conducted an expanded MR analysis using the R10 data from FinnGen, which significantly increased the sample size. Our analysis revealed that PDGF-BB is associated with an increased risk of VTE, DVT, and PE. Notably, even after applying the most stringent Bonferroni correction, the associations between PDGF-BB and both VTE and DVT remained significant. To further validate the robustness of our research findings, this study utilized data from FinnGen R11 (https://www.finngen.fi/en/access_results) and the GWAS Catalog (https://www.ebi.ac.uk/gwas/home), employing five univariable MR methods for external data verification. All results were consistent with our analyses (**Supplementary Table 12**). Numerous observational studies have reported independent associations between VTE and some inflammatory cytokines, such as IL-4, IL-6, IL-8, and monocyte chemoattractant protein-1(MCP-1), which may contribute to a prothrombotic state by stimulating tissue factor expression. We conducted a reverse MR analysis of the causal relationships between inflammatory cytokines and VTE, DVT, and PE (**Supplementary Table 13**). The results indicate that VTE, DVT, and PE lead to alterations in the levels of various inflammatory cytokines, such as CTACK, IL-4, and IL-6. These alterations are likely downstream consequences of disease formation and progression, rather than risk factors that initiate the disease. Consequently, therapies targeting these cytokines may not be effective in halting disease progression at the early stages.

While our research has established a causal relationship between cytokines and VTE at a macro level, the detailed pathogenic mechanisms still require further investigation. The association between inflammatory cytokines and VTE may involve complex interactions among multiple genes, pathways, and immune cell types. The PDGFB gene encodes the PDGF-BB protein, which plays a role in angiogenesis and vascular remodeling. Its receptor, encoded by the PDGFRB gene, activates multiple downstream pathways such as PI3K/AKT and MAPK/ERK pathways upon ligand binding (34). These pathways promote cell survival, proliferation, and migration, potentially affecting vascular integrity and increasing the risk of thrombosis. Key immune cells, like macrophages, are recruited and activated by PDGF-BB to release pro-inflammatory cytokines, promoting thrombus formation and resolution. The function of T lymphocytes is also modulated by PDGF-BB during the inflammatory response. Platelets store PDGF-BB in their alpha granules and release it upon activation, directly participating in thrombosis (35).

To understand the complex interactions between genetic risk genes, inflammatory cytokines, and functional immune cell subgroups and their connection to diseases, Ma et al. (36) (37)introduced an innovative scPagwas method. This method combines single-cell RNA sequencing (scRNA-seq) data with GWAS summary statistics to precisely identify cell types associated with specific traits or diseases. Specifically, it utilizes pathway activation information from scRNA-seq and genetic signals from GWAS to uncover which cells play crucial roles in diseases at the molecular level. The integration of scRNA-seq with GWAS provides a new research approach for unveiling the associations between genes and diseases. By analyzing gene expression differences at the single-cell level, researchers can discover functional differences between cells, thereby better revealing the pathogenic mechanisms of specific diseases.

We discovered that glycocholate reduces the risk of VTE, DVT, and PE by decreasing the levels of PDGF-BB. glycocholate, the anionic form of glycocholic acid, is commonly found in the bile of mammals in the form of its sodium salt. This compound plays a critical role in emulsifying fats and facilitating their digestion and absorption. glycocholic acid is a bile acid synthesized through the conjugation of cholic acid and glycine (38). Previous research has demonstrated that sodium glycocholate can inhibit adenosine diphosphate (ADP) or collagen-induced platelet aggregation *in vitro* (39). Despite limited studies on the interplay among blood metabolites, inflammatory cytokines, and VTE, recent research has increasingly recognized the correlations between cytokines and metabolites. A study focusing on drug-induced liver injury revealed that the levels of numerous metabolites related to primary bile acid biosynthesis, α-linolenic acid metabolism, and phospholipid metabolism pathways were negatively correlated with the concentrations of pro-inflammatory cytokines (PDGF-BB, TNF-α, IP-10, and MIP-1b) and the anti-inflammatory cytokine (IL-1Rα) (40). These findings provide robust evidence for the interaction between metabolic pathways and immune status, offering insightful perspectives on the potential associations between blood metabolites, inflammatory cytokines, and thrombotic diseases.

Our study offers several advantages. Firstly, this is the inaugural comprehensive MR study that explores the relationships between blood metabolites, inflammatory cytokines, and VTE (including DVT and PE). It further probes the potential associations among them through mediation analysis. Secondly, it minimizes confounding factors, thereby establishing a more reliable causative relationship compared to observational studies. Thirdly, it utilizes the latest publicly available GWAS databases, providing a robust data foundation. Lastly, compared to time-consuming RCTs, it is more cost-effective, requiring fewer resources and less time.

However, this study also has certain limitations. Firstly, in the validation of mediation effects using MVMR, the 95% confidence interval for the association between PDGF-BB-mediated dodecanedioate (C12:1-DC) levels and VTE or DVT includes zero, it indicates that we do not have sufficient evidence to reject the hypothesis that the mediation effect is zero. Consequently, more data or further research is required to confirm this effect. Secondly, our study preliminarily explored the causal relationship between metabolites, inflammatory cytokines, and VTE. Although MR studies theoretically provide strong causal inference, there are currently relatively few laboratory and clinical studies on this association. Therefore, these preliminary findings still need to be validated through further clinical trials and mechanistic studies. Thirdly, this study primarily focuses on populations of European descent, which may introduce racial bias and limit the broad applicability of the research findings. Therefore, caution should be exercised when applying these findings to other ethnic groups.

## Data Availability

The original contributions presented in the study are included in the article/**Supplementary Material**. Further inquiries can be directed to the corresponding author.
