# Supplementary material for: Potential causal relationships between blood metabolites, inflammatory cytokines, and venous thromboembolism
Source: Front Immunol. 2024 Sep 30;15:1445790. doi: 10.3389/fimmu.2024.1445790 (PMC11471515; doi:10.3389/fimmu.2024.1445790)
Supplement: Supplementary file 1 [file DataSheet1.zip › Supplementary Figures.docx]

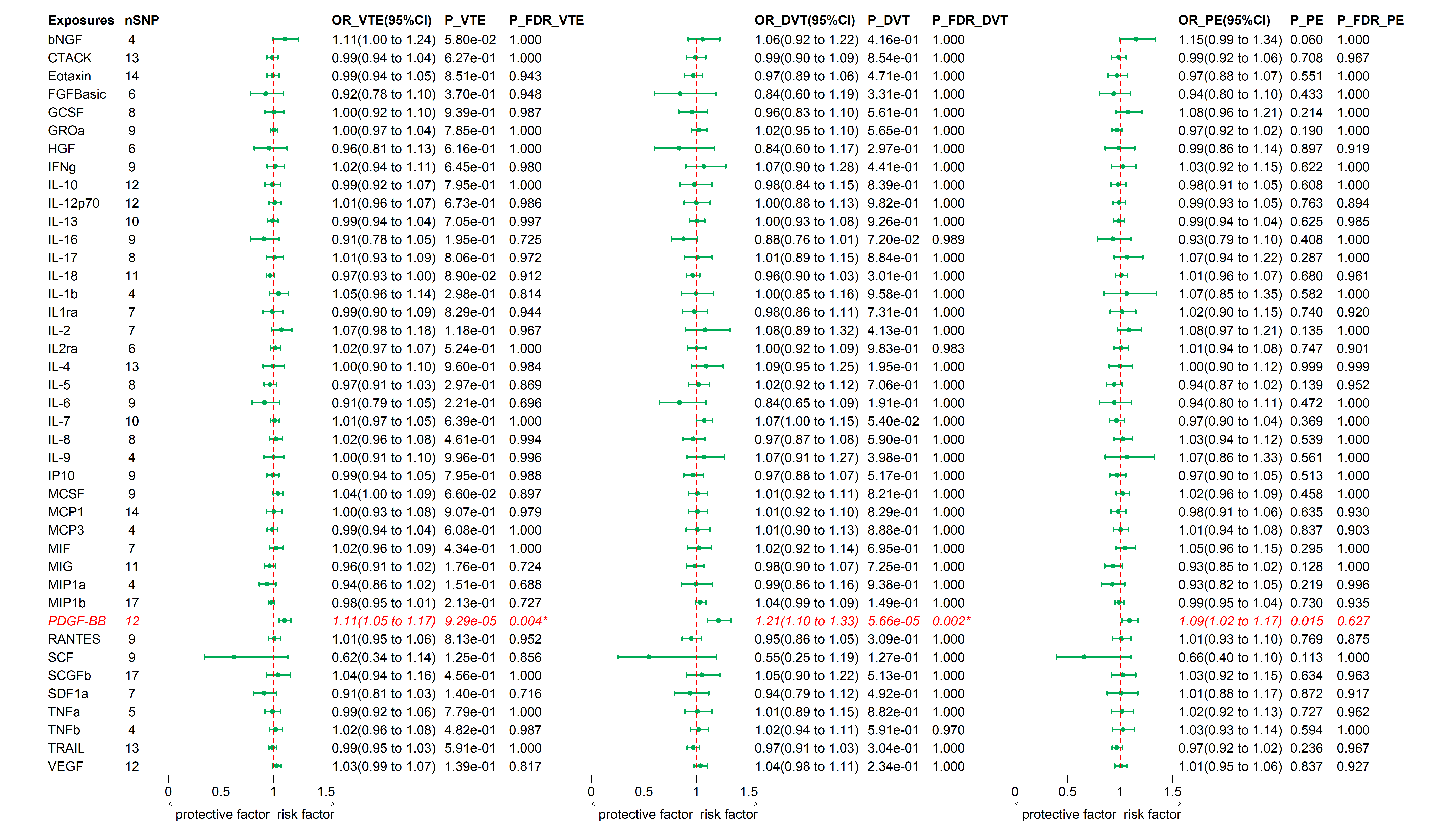


**Supplementary Figure 1: Causal Relationships of 41 Inflammatory Cytokines with Venous Thromboembolism, Deep Vein Thrombosis, and Pulmonary Embolism.**

The red color indicates cytokines with a causal effect (P-Value < 0.05) on the outcome. bNGF, Beta Nerve Growth Factor; CTACK, Cutaneous T Cell-Attracting Chemokine; FGFBasic, Basic Fibroblast Growth Factor; GCSF, Granulocyte Colony-Stimulating Factor; GROa, Growth-Regulated Oncogene-α; HGF, Hepatocyte Growth Factor; IFNg, Interferon Gamma; IL, Interleukin; IP10, Interferon Gamma-Induced Protein 10; MCP1, Monocyte Chemotactic Protein-1; MCP3, Monocyte Specific Chemokine 3; MCSF, Macrophage Colony-Stimulating Factor; MIF, Macrophage Migration Inhibitory Factor; MIG, Monokine Induced by Interferon Gamma; MIP1a, Macrophage Inflammatory Protein-1α; MIP1b, Macrophage Inflammatory Protein-1β; PDGF-BB, Platelet Derived Growth Factor BB; RANTES, Regulated Upon Activation, Normal T Cell Expressed and Secreted Factor; SCF, Stem Cell Factor; SCGFb, Stem Cell Growth Factor Beta; SDF1a, Stromal Cell-Derived Factor-1 Alpha; TNFa, Tumor Necrosis Factor Alpha; TNFb, Tumor Necrosis Factor Beta; TRAIL, TNF-Related Apoptosis Inducing Ligand; VEGF, Vascular Endothelial Growth Factor; SNP, number of Single Nucleotide Polymorphisms; OR, Odds Ratio; P, P-value; P_FDR, P-value adjusted for False Discovery Rate; VTE, Venous Thromboembolism; DVT, Deep Vein Thrombosis; PE, Pulmonary Embolism.

| **A** | **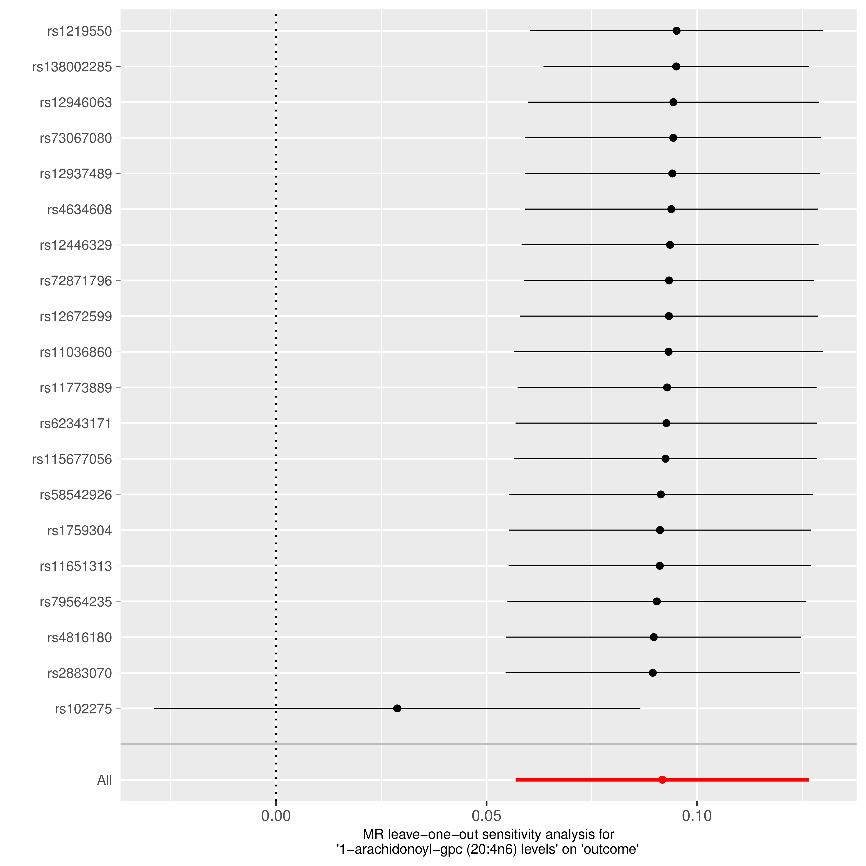** | **B** | **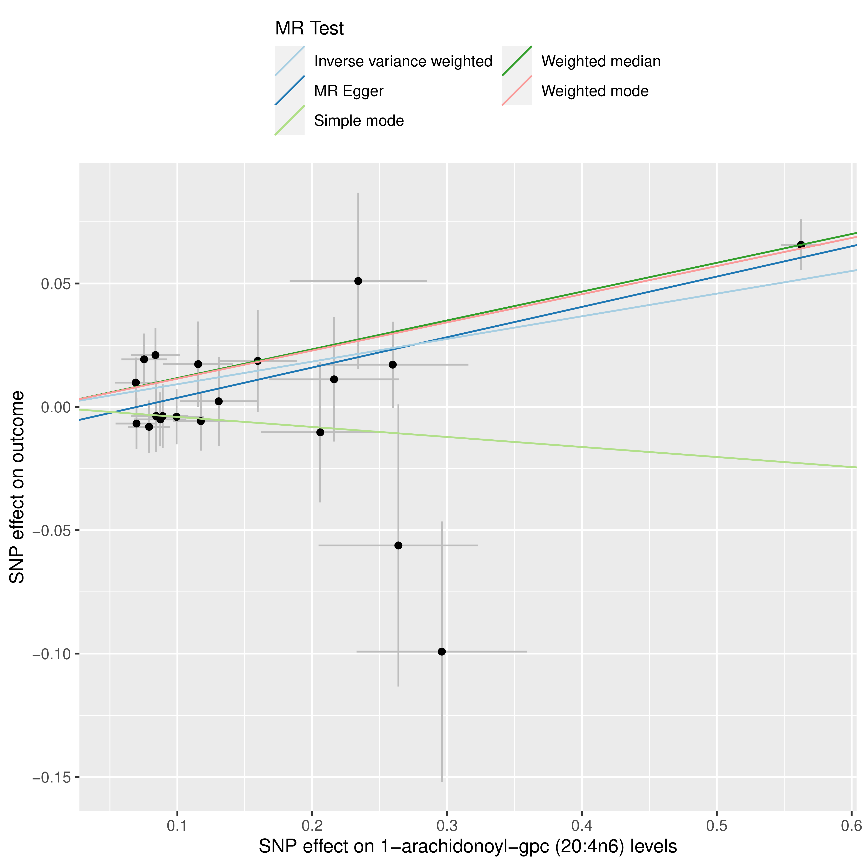** |
| --- | --- | --- | --- |
| **C** | **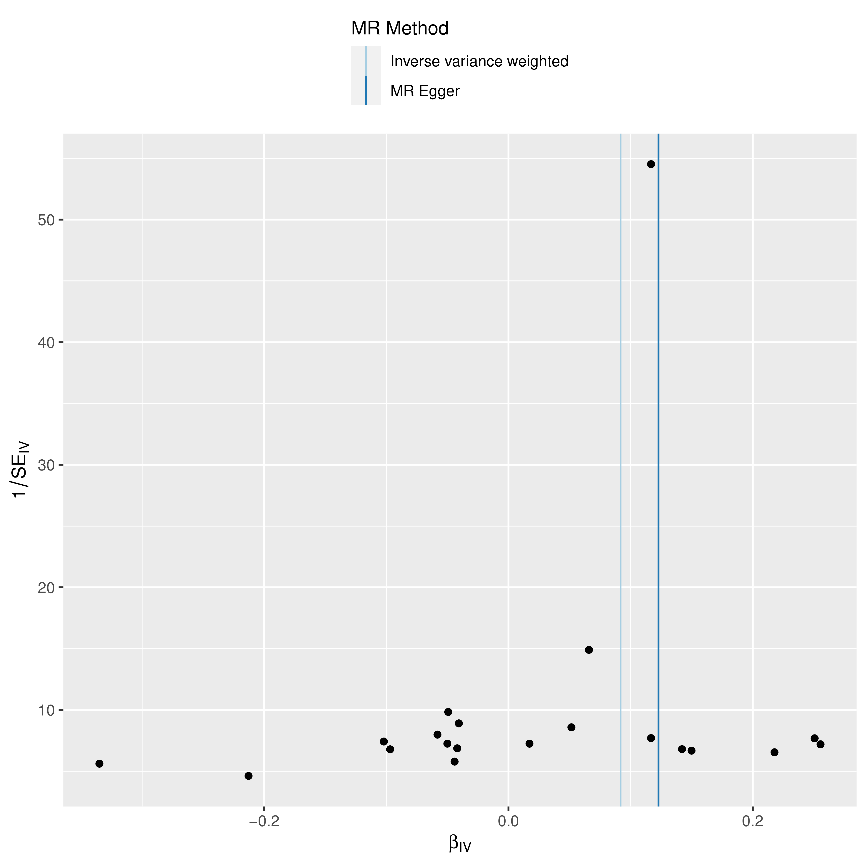** | **D** | **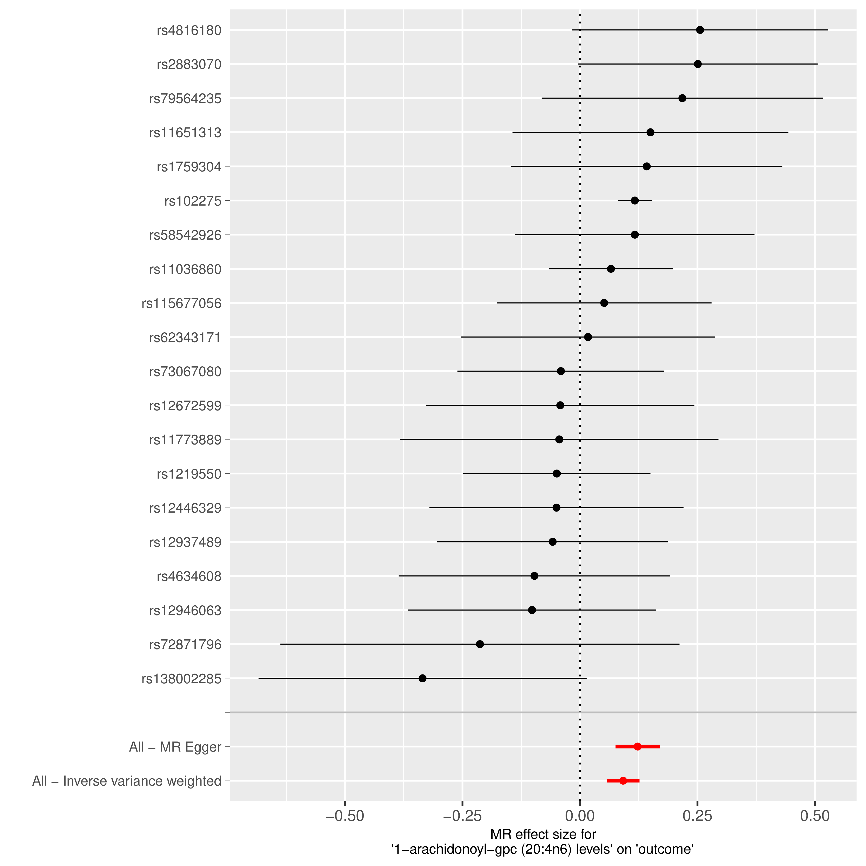** |

**Supplementary Figure 2: Analyses of the Association Between 1-Arachidonoyl-GPC (20:4n6) Levels and** **Venous Thromboembolism Risk.** (A) Leave-One-Out Analysis: Assessment of the influence of individual SNPs associated with 1-Arachidonoyl-GPC (20:4n6) Levels on Venous Thromboembolism risk. (B) Scatter Plot: Visualization of the causal relationship between individual SNPs within 1-Arachidonoyl-GPC (20:4n6) Levels and Venous Thromboembolism risk. (C) Funnel Plot: Examination of the overall heterogeneity in Mendelian Randomization estimates for the impact of 1-Arachidonoyl-GPC (20:4n6) Levels on Venous Thromboembolism risk. (D) Forest Plot: Evaluation of the causal effects of individual SNPs on Venous Thromboembolism risk. MR, Mendelian Randomization; SNP, Single Nucleotide Polymorphism.

| **A** | **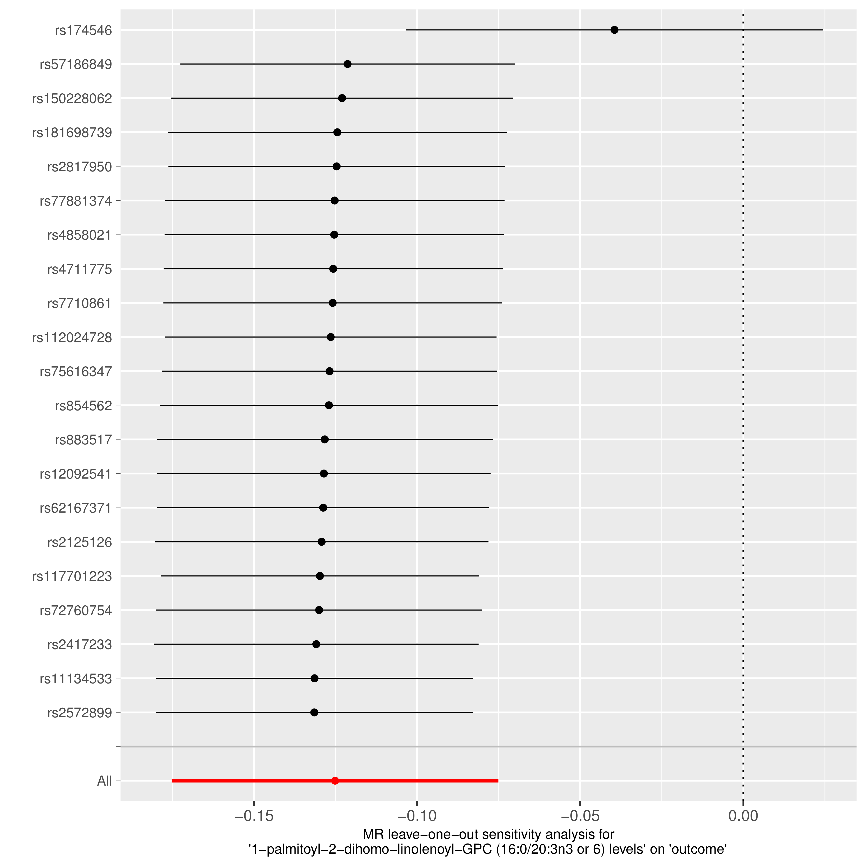** | **B** | **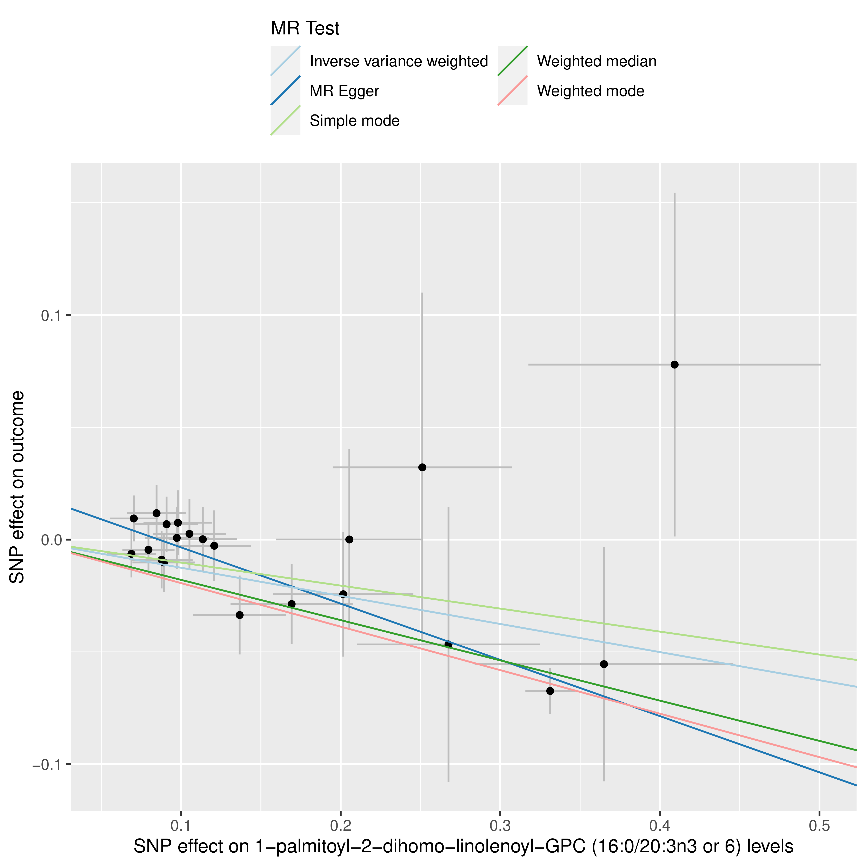** |
| --- | --- | --- | --- |
| **C** | **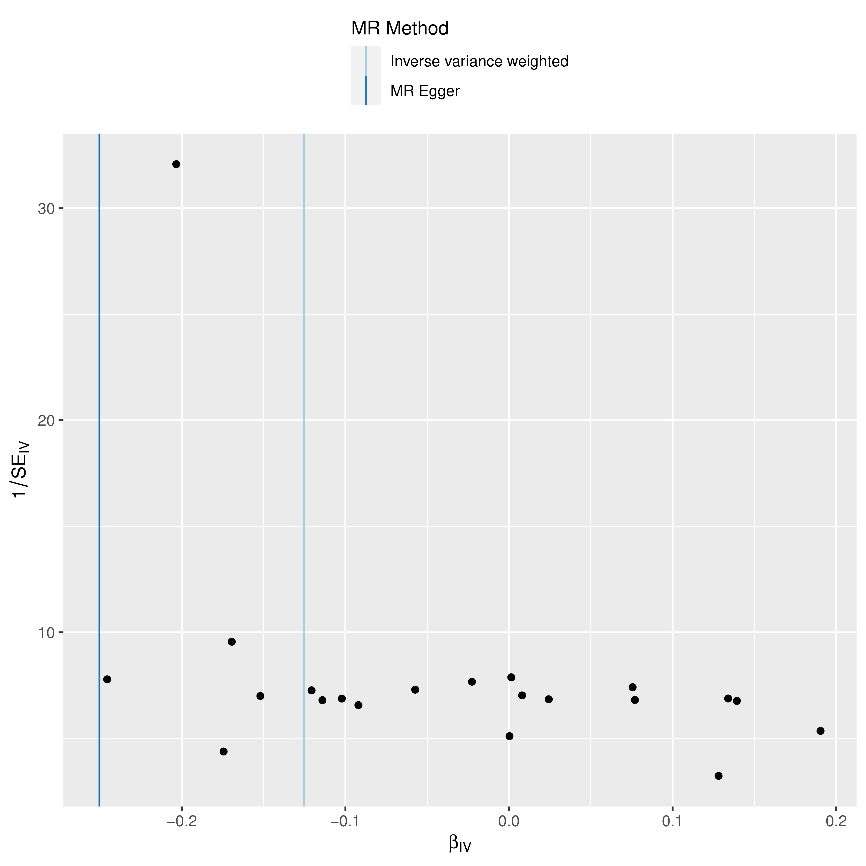** | **D** | **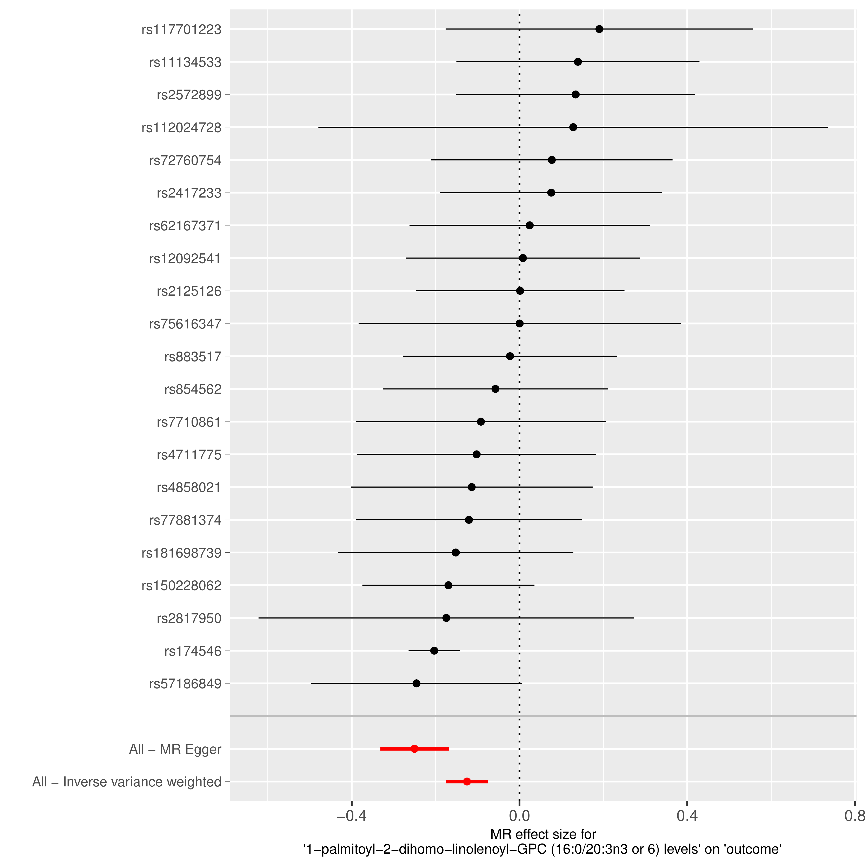** |

**Supplementary Figure 3: Analyses of the Association Between 1-palmitoyl-2-dihomo-linolenoyl-GPC (16:0/20:3n3 or 6) Levels and** **Venous Thromboembolism Risk.** (A) Leave-One-Out Analysis: Assessment of the influence of individual SNPs associated with 1-palmitoyl-2-dihomo-linolenoyl-GPC (16:0/20:3n3 or 6) levels on Venous Thromboembolism risk. (B) Scatter Plot: Visualization of the causal relationship between individual SNPs within 1-palmitoyl-2-dihomo-linolenoyl-GPC (16:0/20:3n3 or 6) levels and Venous Thromboembolism risk. (C) Funnel Plot: Examination of the overall heterogeneity in MR estimates for the impact of 1-palmitoyl-2-dihomo-linolenoyl-GPC (16:0/20:3n3 or 6) levels on Venous Thromboembolism risk. (D) Forest Plot: Evaluation of the causal effects of individual SNPs on Venous Thromboembolism risk. MR, Mendelian Randomization; SNP, Single Nucleotide Polymorphism.

| **A** | **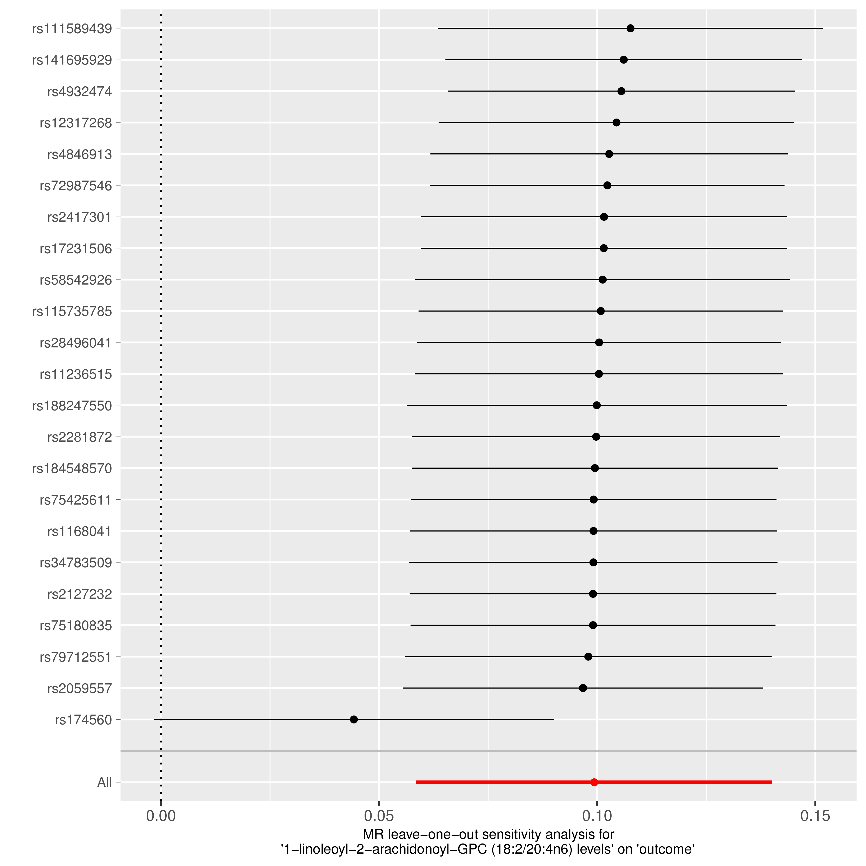** | **B** | **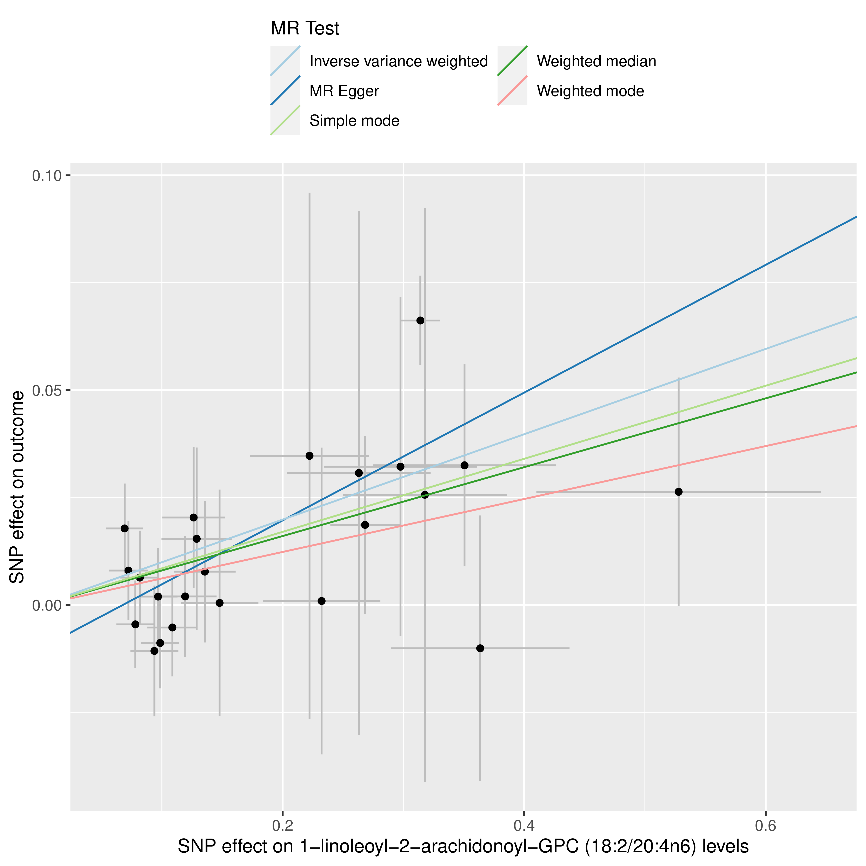** |
| --- | --- | --- | --- |
| **C** | **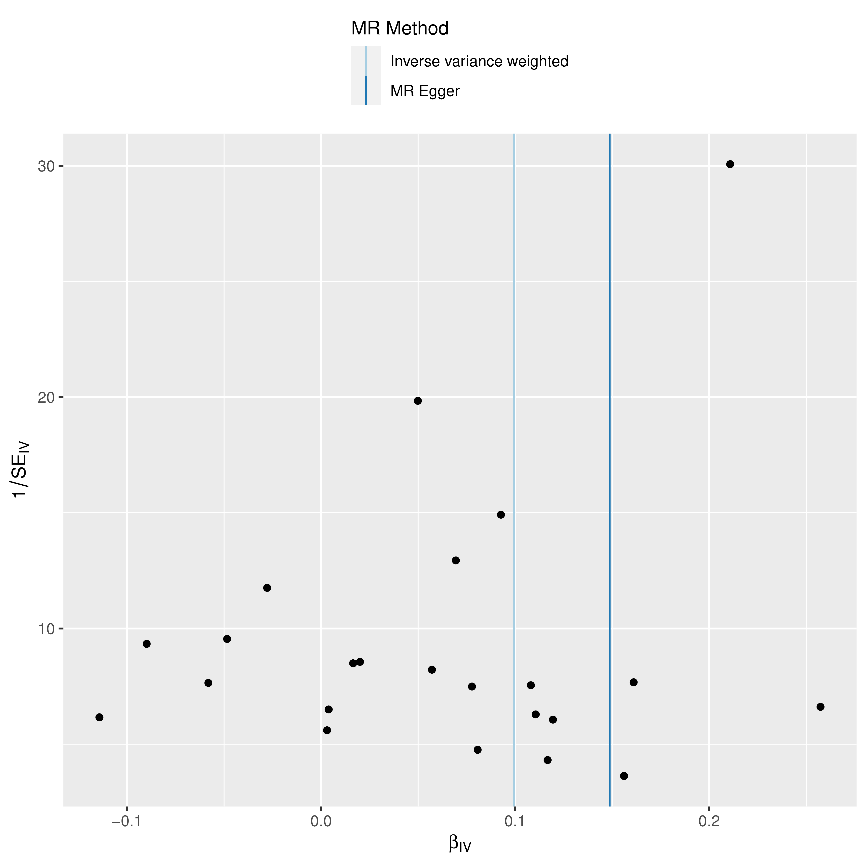** | **D** | **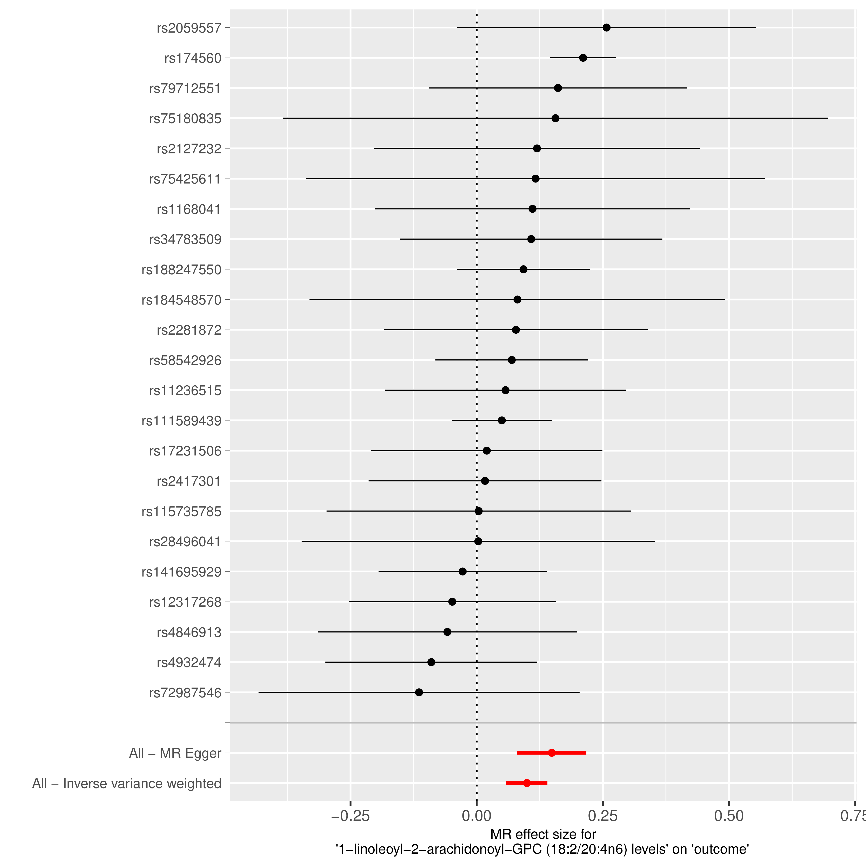** |

**Supplementary Figure 4: Analyses of the Association Between 1-linoleoyl-2-arachidonoyl-GPC (18:2/20:4n6) Levels and** **Venous Thromboembolism Risk.** (A) Leave-One-Out Analysis: Assessment of the influence of individual SNPs associated with 1-linoleoyl-2-arachidonoyl-GPC (18:2/20:4n6) levels on Venous Thromboembolism risk. (B) Scatter Plot: Visualization of the causal relationship between individual SNPs within 1-linoleoyl-2-arachidonoyl-GPC (18:2/20:4n6) levels and Venous Thromboembolism risk. (C) Funnel Plot: Examination of the overall heterogeneity in MR estimates for the impact of 1-linoleoyl-2-arachidonoyl-GPC (18:2/20:4n6) levels on Venous Thromboembolism risk. (D) Forest Plot: Evaluation of the causal effects of individual SNPs on Venous Thromboembolism risk. MR, Mendelian Randomization; SNP, Single Nucleotide Polymorphism.

| **A** | **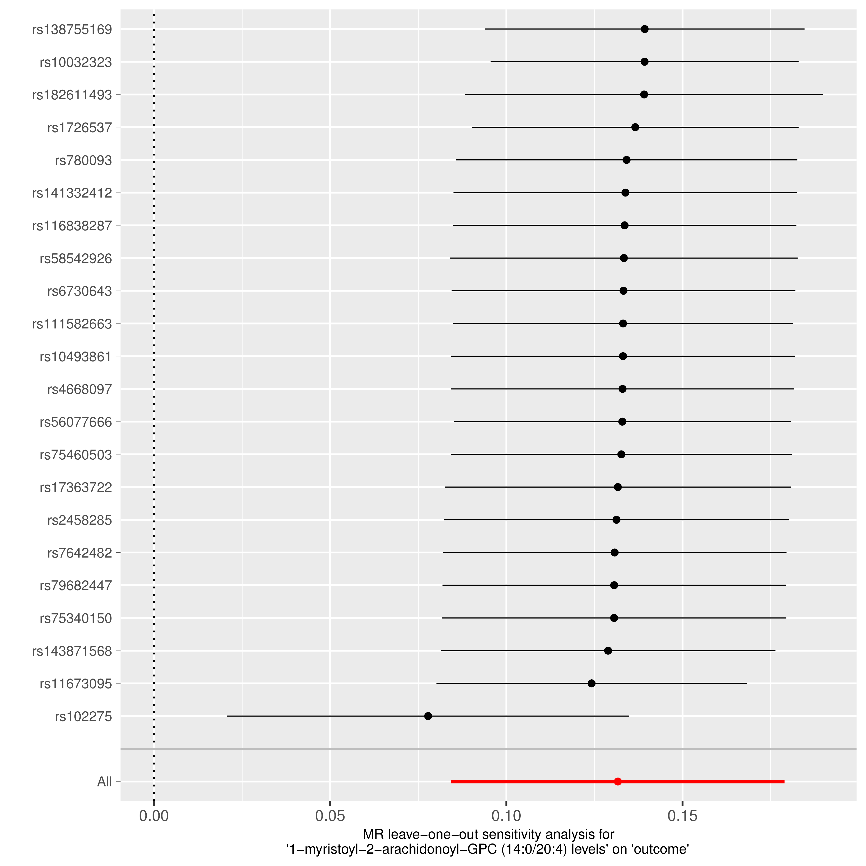** | **B** | **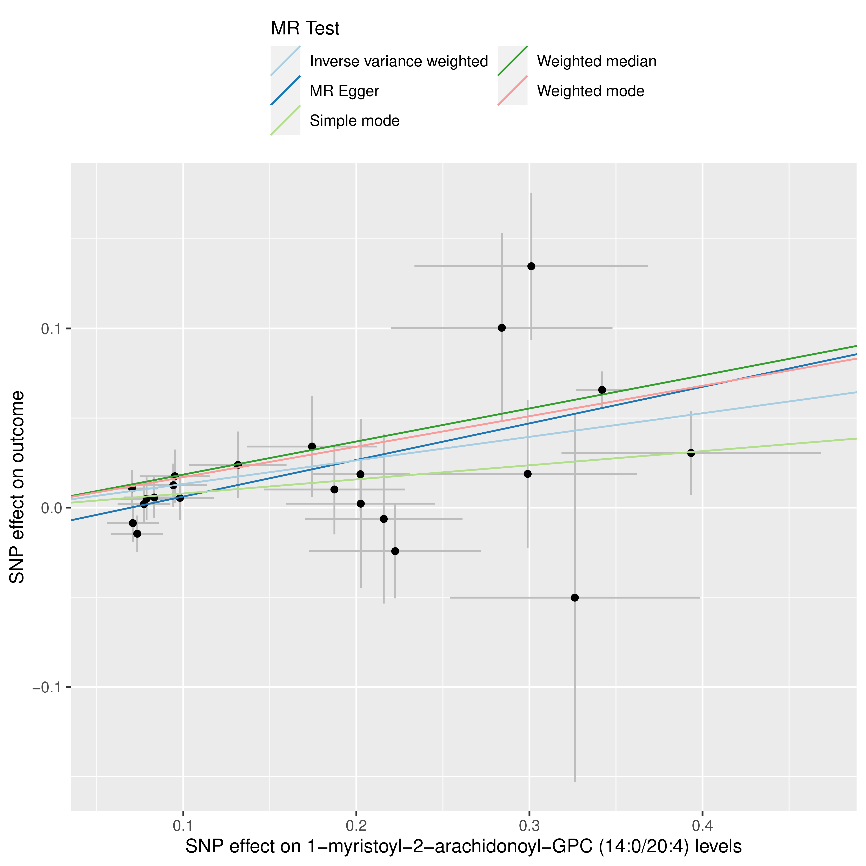** |
| --- | --- | --- | --- |
| **C** | **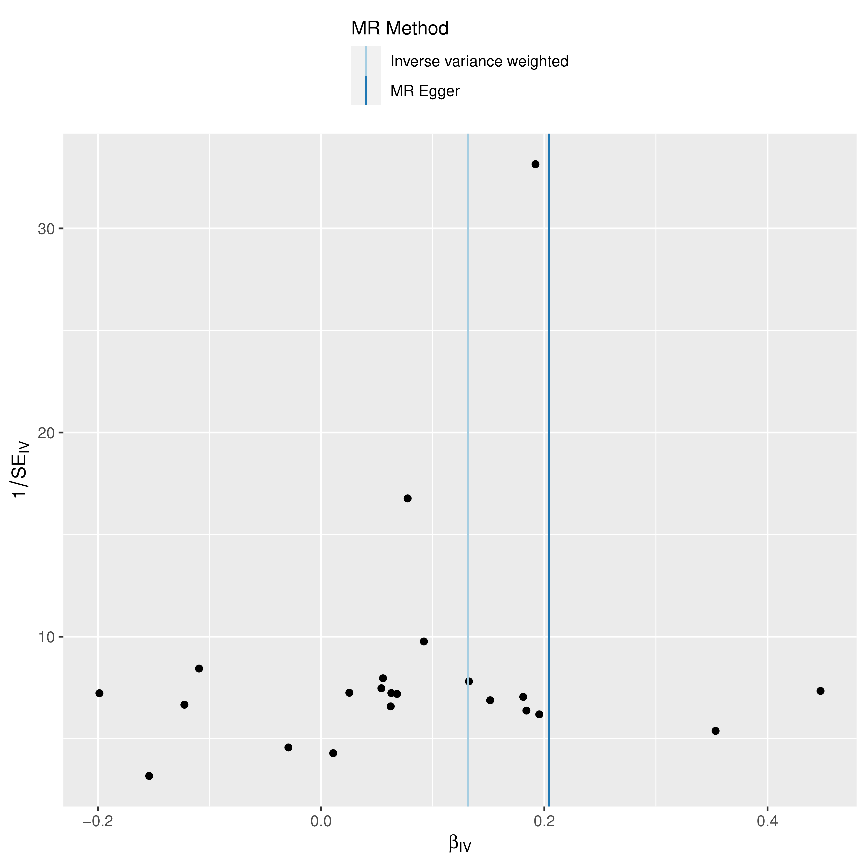** | **D** | **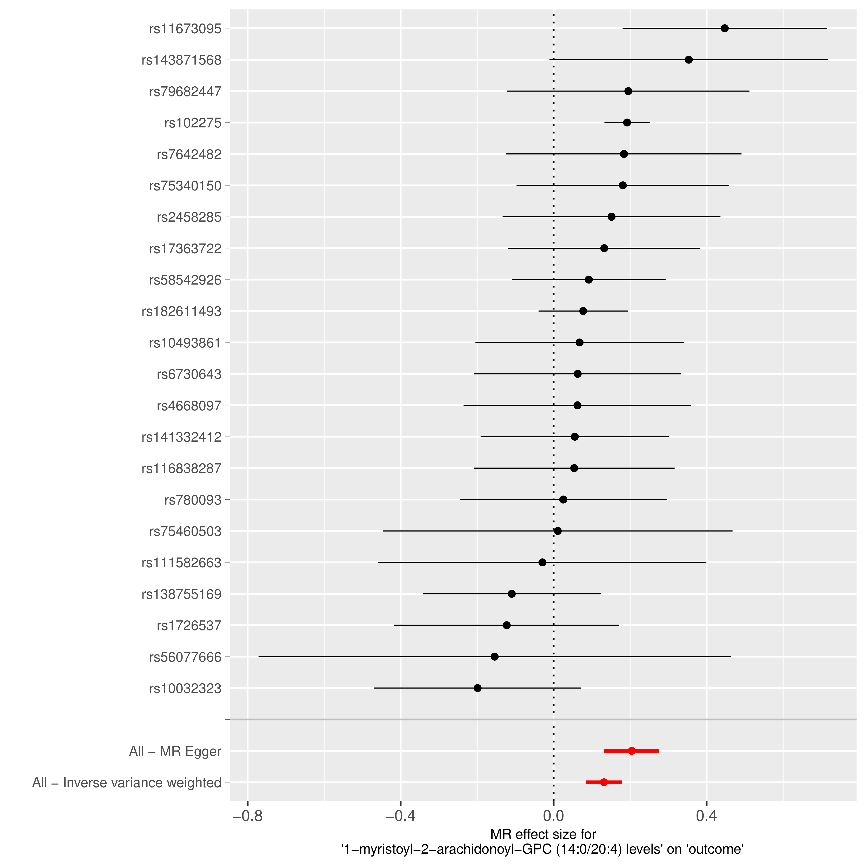** |

**Supplementary Figure 5: Analyses of the Association Between 1-myristoyl-2-arachidonoyl-GPC (14:0/20:4) Levels and Venous Thromboembolism Risk.** (A) Leave-One-Out Analysis: Assessment of the influence of individual SNPs associated with 1-myristoyl-2-arachidonoyl-GPC (14:0/20:4) levels on Venous Thromboembolism risk. (B) Scatter Plot: Visualization of the causal relationship between individual SNPs within 1-myristoyl-2-arachidonoyl-GPC (14:0/20:4) levels and Venous Thromboembolism risk. (C) Funnel Plot: Examination of the overall heterogeneity in MR estimates for the impact of 1-myristoyl-2-arachidonoyl-GPC (14:0/20:4) levels on Venous Thromboembolism risk. (D) Forest Plot: Evaluation of the causal effects of individual SNPs on Venous Thromboembolism risk. MR, Mendelian Randomization; SNP, Single Nucleotide Polymorphism.

| **A** | **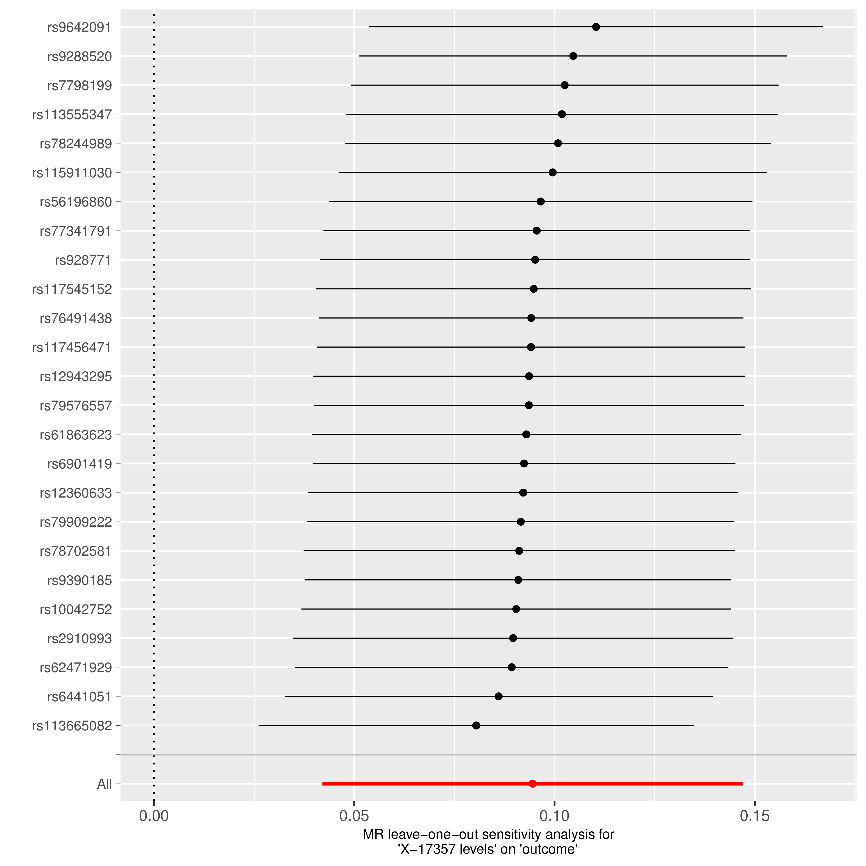** | **B** | **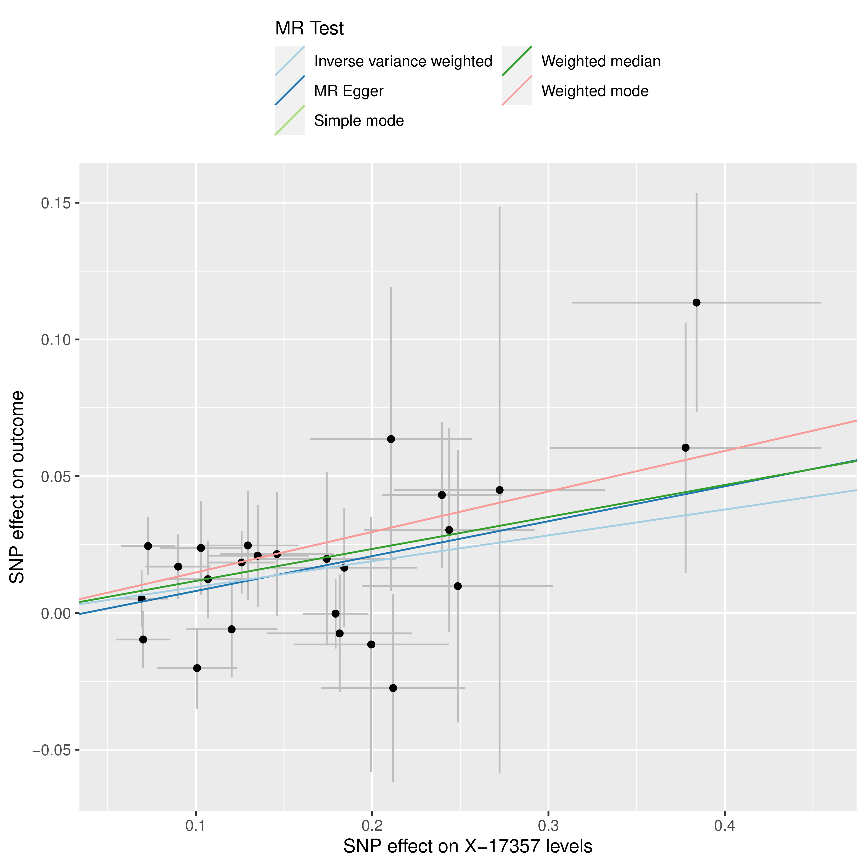** |
| --- | --- | --- | --- |
| **C** | **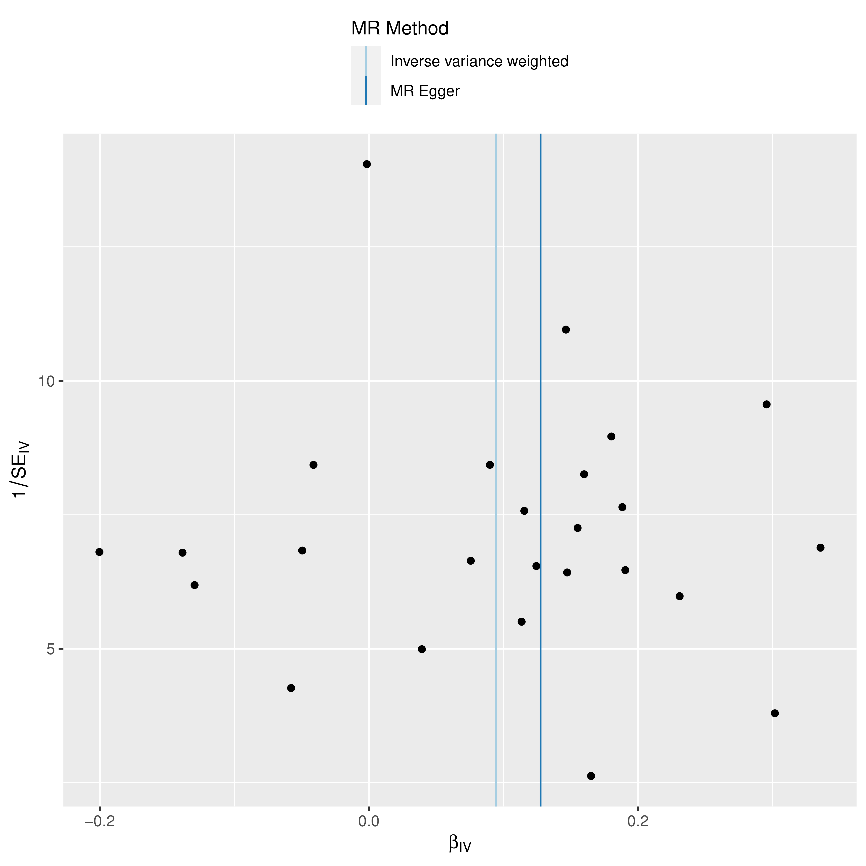** | **D** | **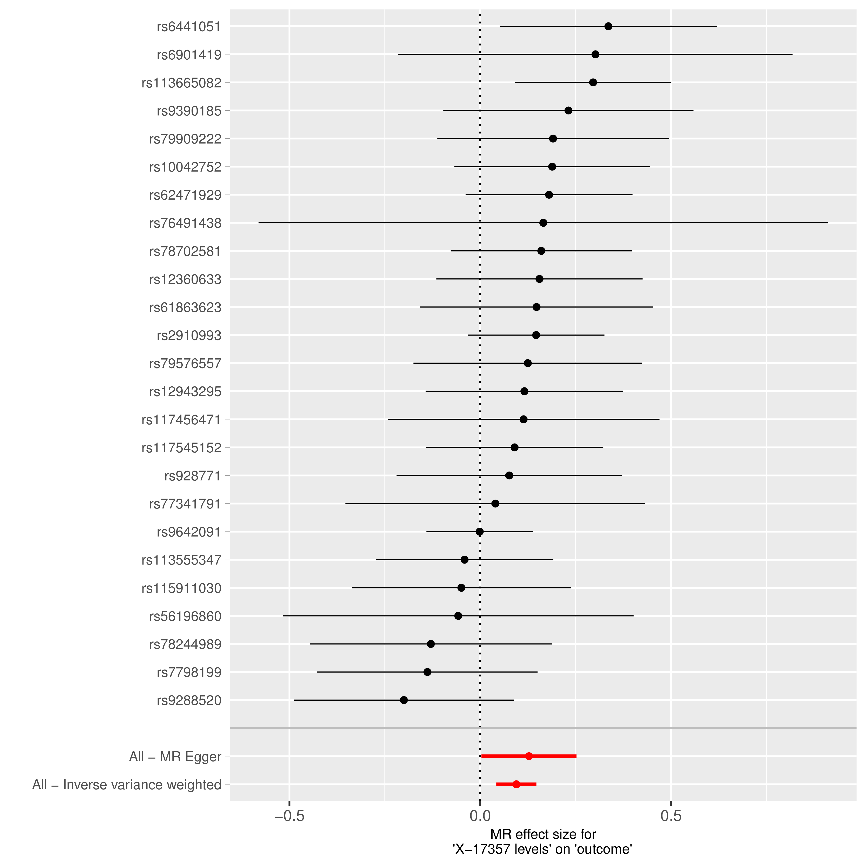** |

**Supplementary Figure 6: Analyses of the Association Between X-17357 Levels and Venous Thromboembolism Risk.** (A) Leave-One-Out Analysis: Assessment of the influence of individual SNPs associated with X-17357 levels on Venous Thromboembolism risk. (B) Scatter Plot: Visualization of the causal relationship between individual SNPs within X-17357 levels and Venous Thromboembolism risk. (C) Funnel Plot: Examination of the overall heterogeneity in MR estimates for the impact of X-17357 levels on Venous Thromboembolism risk. (D) Forest Plot: Evaluation of the causal effects of individual SNPs on Venous Thromboembolism risk. MR, Mendelian Randomization; SNP, Single Nucleotide Polymorphism.

| **A** | **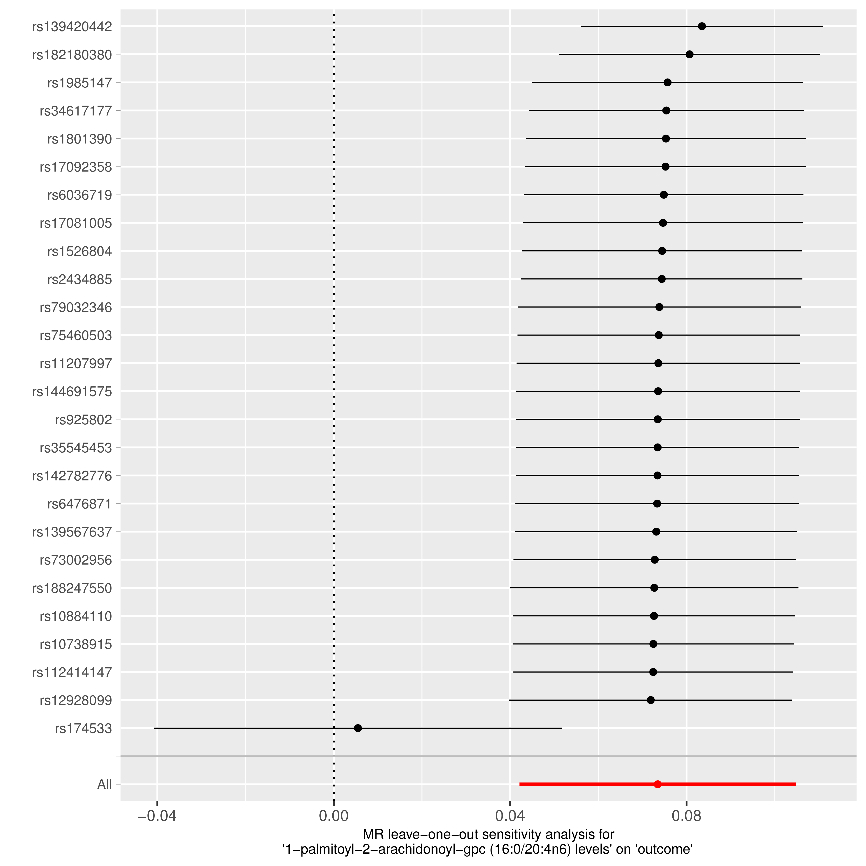** | **B** | **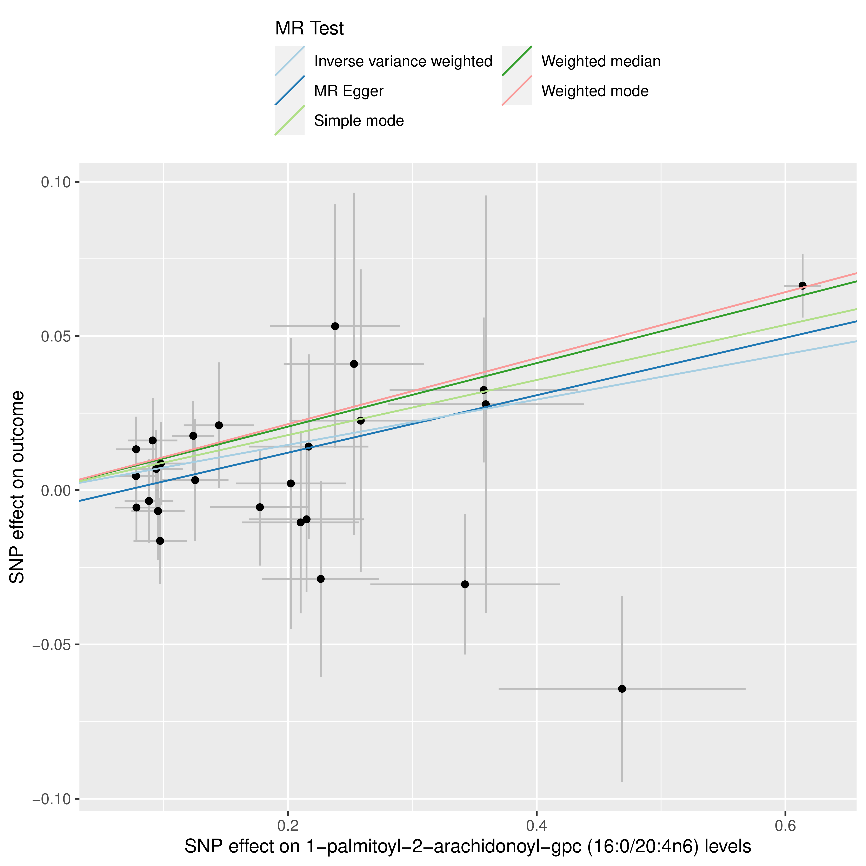** |
| --- | --- | --- | --- |
| **C** | **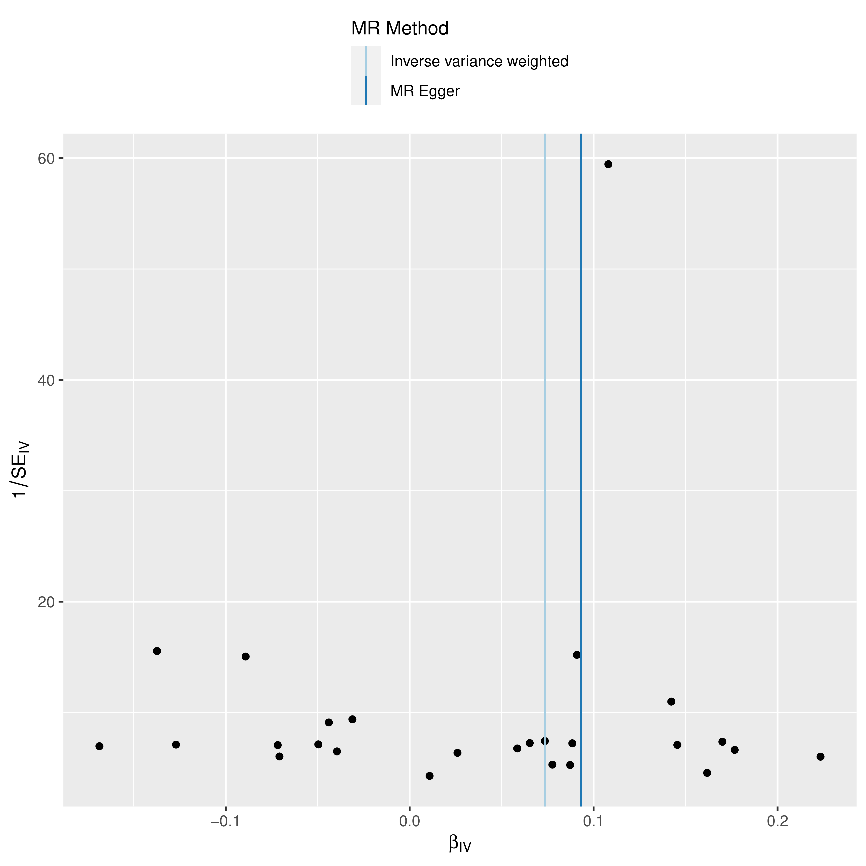** | **D** | **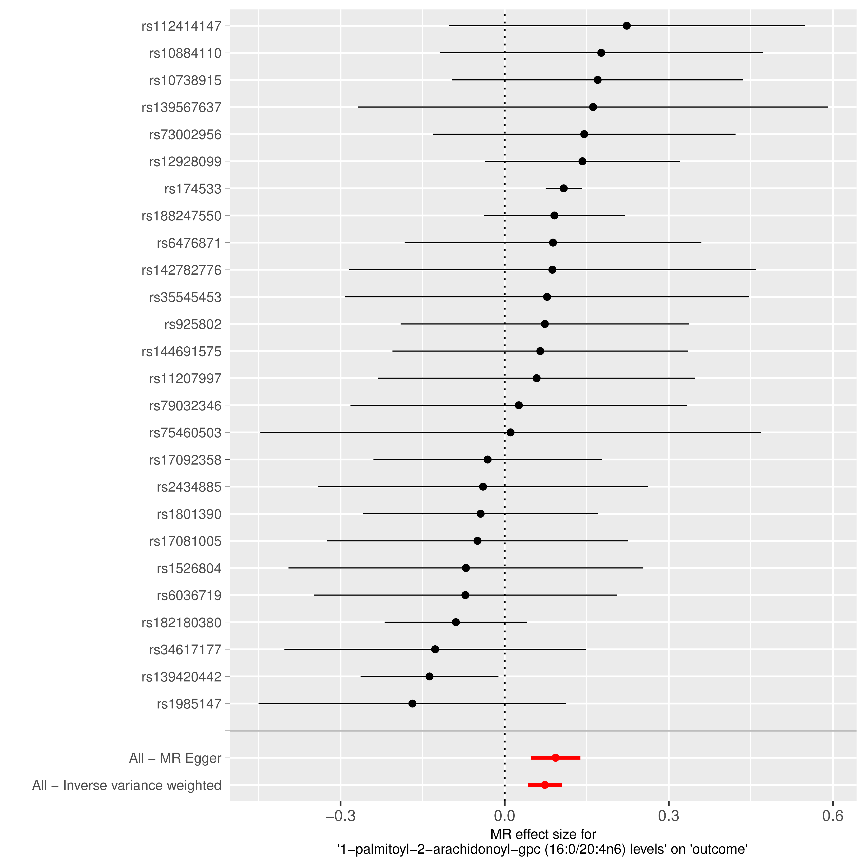** |

**Supplementary Figure 7: Analyses of the Association Between 1-palmitoyl-2-arachidonoyl-gpc (16:0/20:4n6) Levels and Venous Thromboembolism Risk.** (A) Leave-One-Out Analysis: Assessment of the influence of individual SNPs associated with 1-palmitoyl-2-arachidonoyl-gpc (16:0/20:4n6) levels on Venous Thromboembolism risk. (B) Scatter Plot: Visualization of the causal relationship between individual SNPs within 1-palmitoyl-2-arachidonoyl-gpc (16:0/20:4n6) levels and Venous Thromboembolism risk. (C) Funnel Plot: Examination of the overall heterogeneity in MR estimates for the impact of 1-palmitoyl-2-arachidonoyl-gpc (16:0/20:4n6) levels on Venous Thromboembolism risk. (D) Forest Plot: Evaluation of the causal effects of individual SNPs on Venous Thromboembolism risk. MR, Mendelian Randomization; SNP, Single Nucleotide Polymorphism.

| **A** | **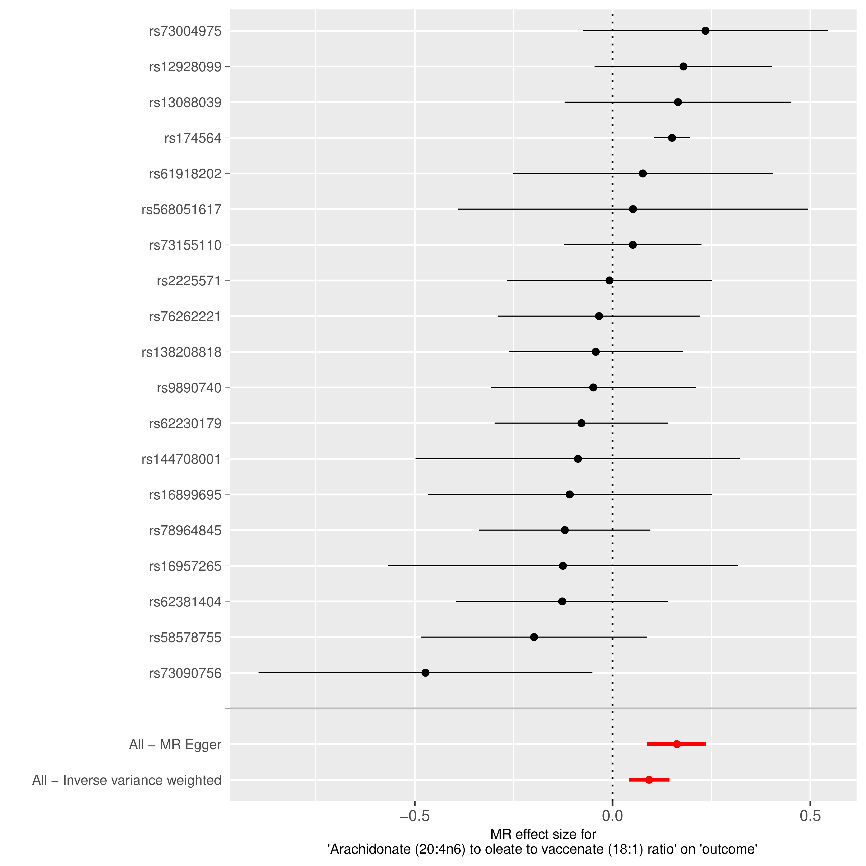** | **B** | **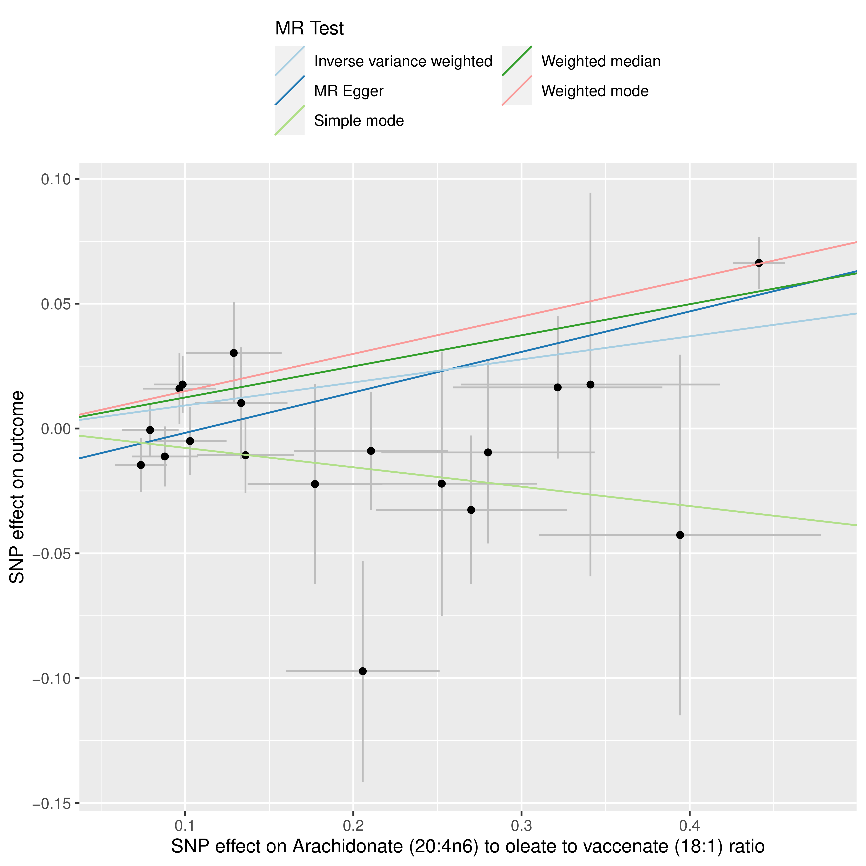** |
| --- | --- | --- | --- |
| **C** | **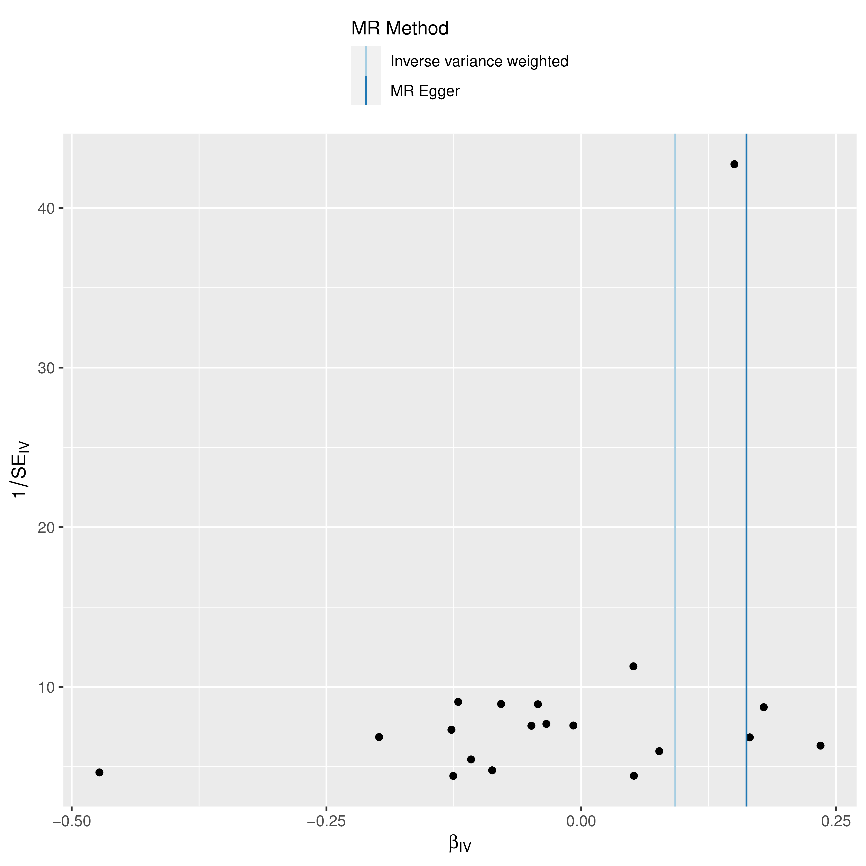** | **D** | **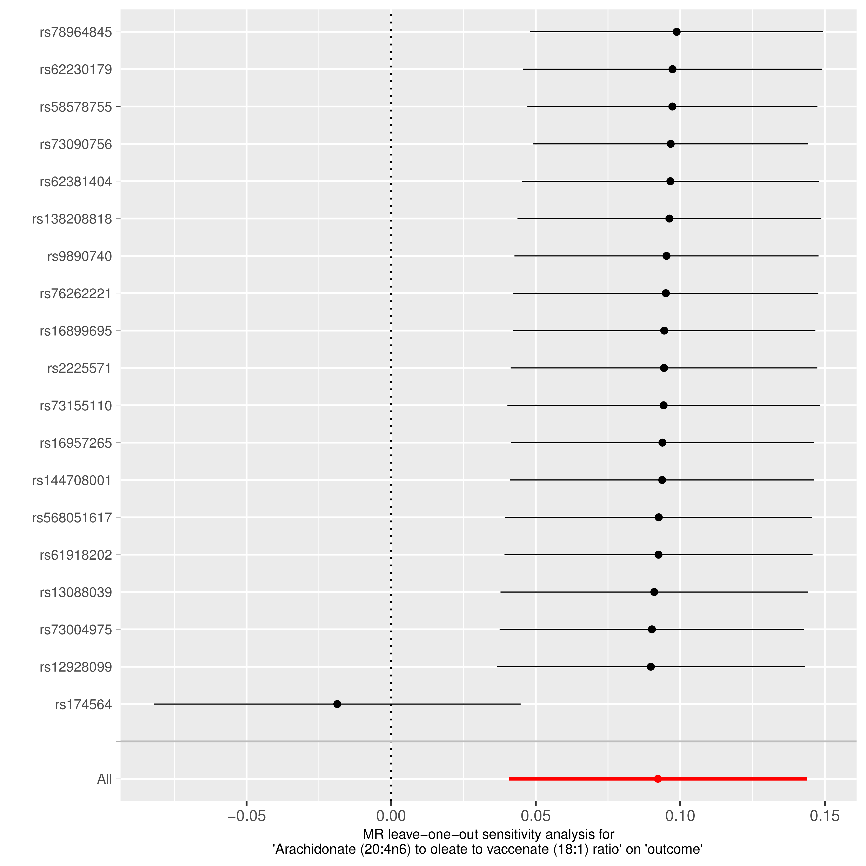** |

**Supplementary Figure 8: Analyses of the Association Between Arachidonate (20:4n6) to oleate to vaccenate (18:1) ratio and Venous Thromboembolism Risk**. (A) Leave-One-Out Analysis: Assessment of the influence of individual SNPs associated with Arachidonate (20:4n6) to oleate to vaccenate (18:1) ratio on Venous Thromboembolism risk. (B) Scatter Plot: Visualization of the causal relationship between individual SNPs within Arachidonate (20:4n6) to oleate to vaccenate (18:1) ratio and Venous Thromboembolism risk. (C) Funnel Plot: Examination of the overall heterogeneity in MR estimates for the impact of Arachidonate (20:4n6) to oleate to vaccenate (18:1) ratio on Venous Thromboembolism risk. (D) Forest Plot: Evaluation of the causal effects of individual SNPs on Venous Thromboembolism risk. MR, Mendelian Randomization; SNP, Single Nucleotide Polymorphism.

| **A** | **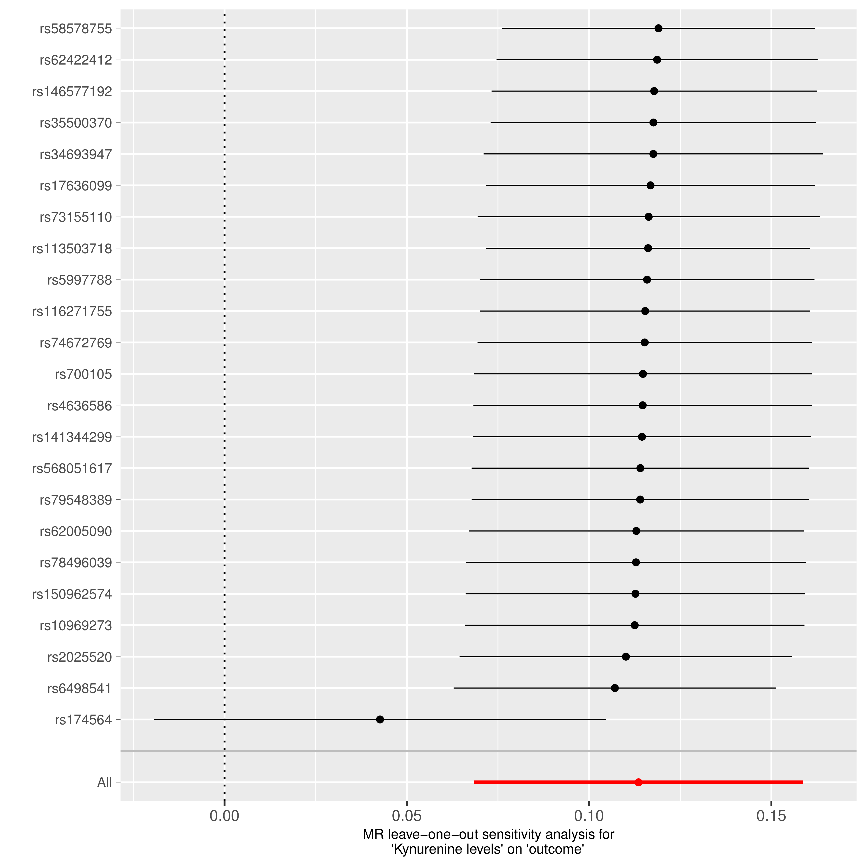** | **B** | **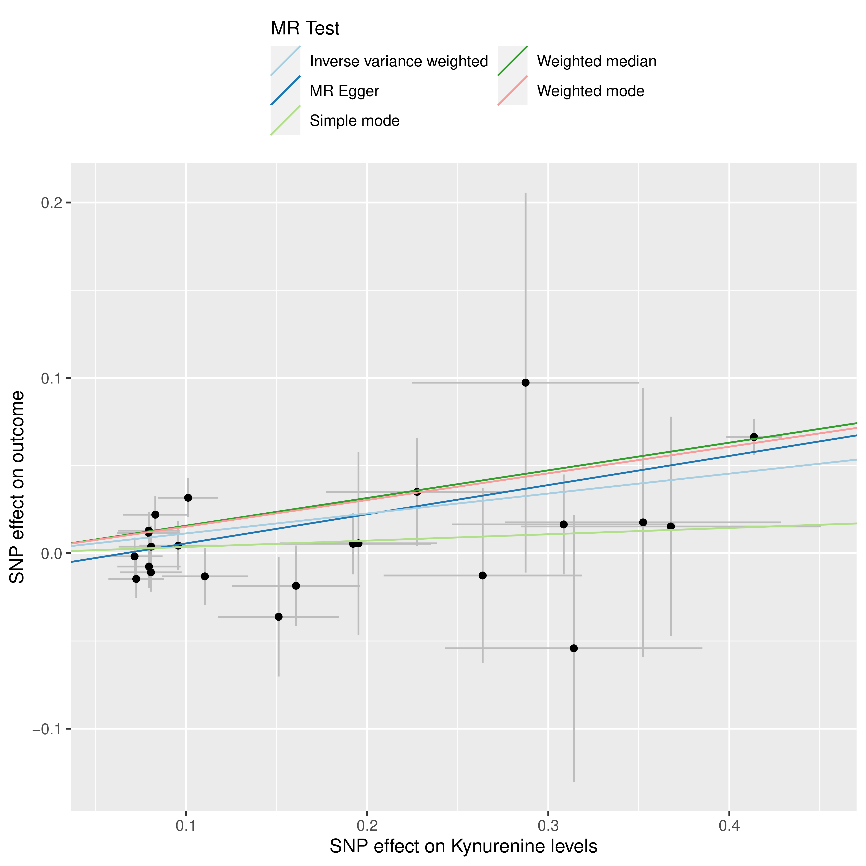** |
| --- | --- | --- | --- |
| **C** | **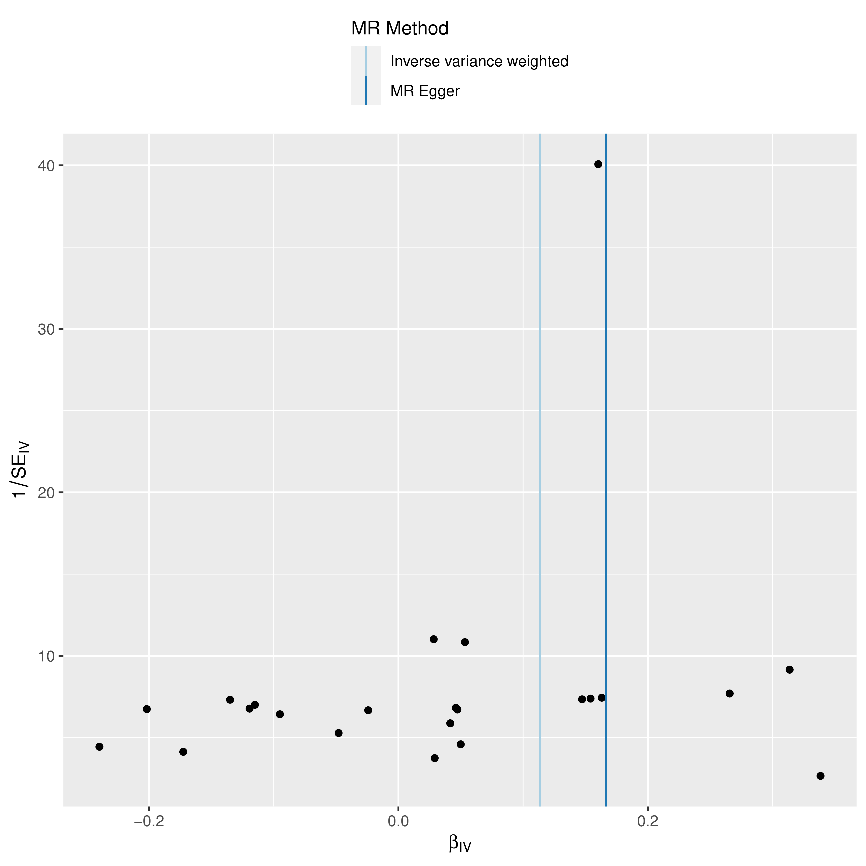** | **D** | **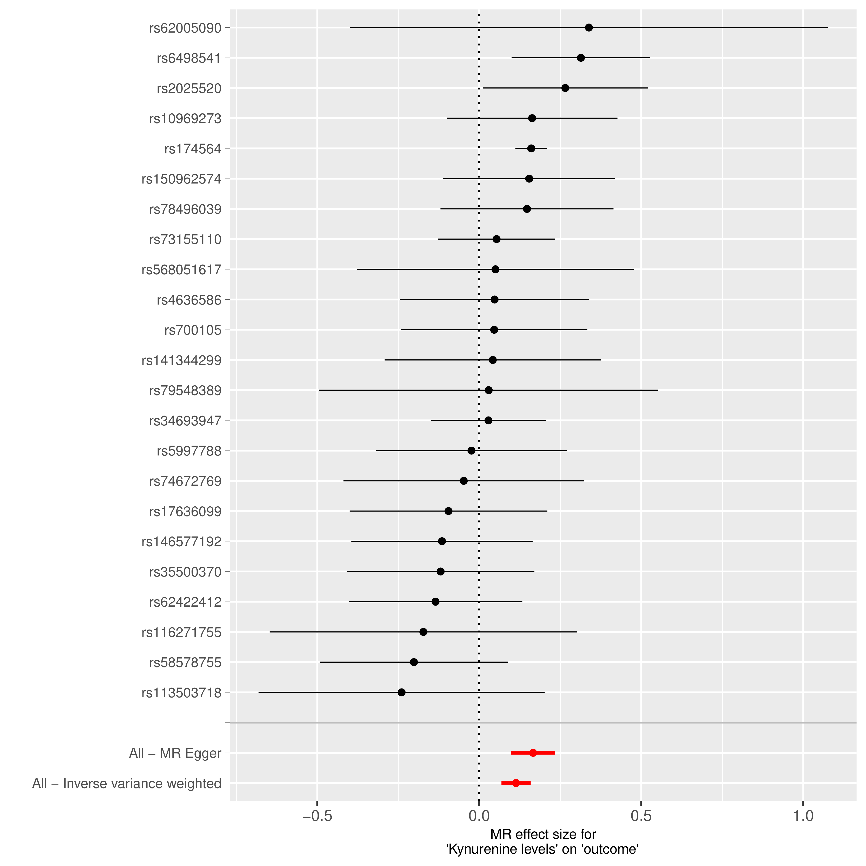** |

**Supplementary Figure 9: Analyses of the Association Between Kynurenine Levels and Venous Thromboembolism Risk.** (A) Leave-One-Out Analysis: Assessment of the influence of individual SNPs associated with Kynurenine levels on Venous Thromboembolism risk. (B) Scatter Plot: Visualization of the causal relationship between individual SNPs within Kynurenine levels and Venous Thromboembolism risk. (C) Funnel Plot: Examination of the overall heterogeneity in MR estimates for the impact of Kynurenine levels on Venous Thromboembolism risk. (D) Forest Plot: Evaluation of the causal effects of individual SNPs on Venous Thromboembolism risk. MR, Mendelian Randomization; SNP, Single Nucleotide Polymorphism.

.

| **A** | **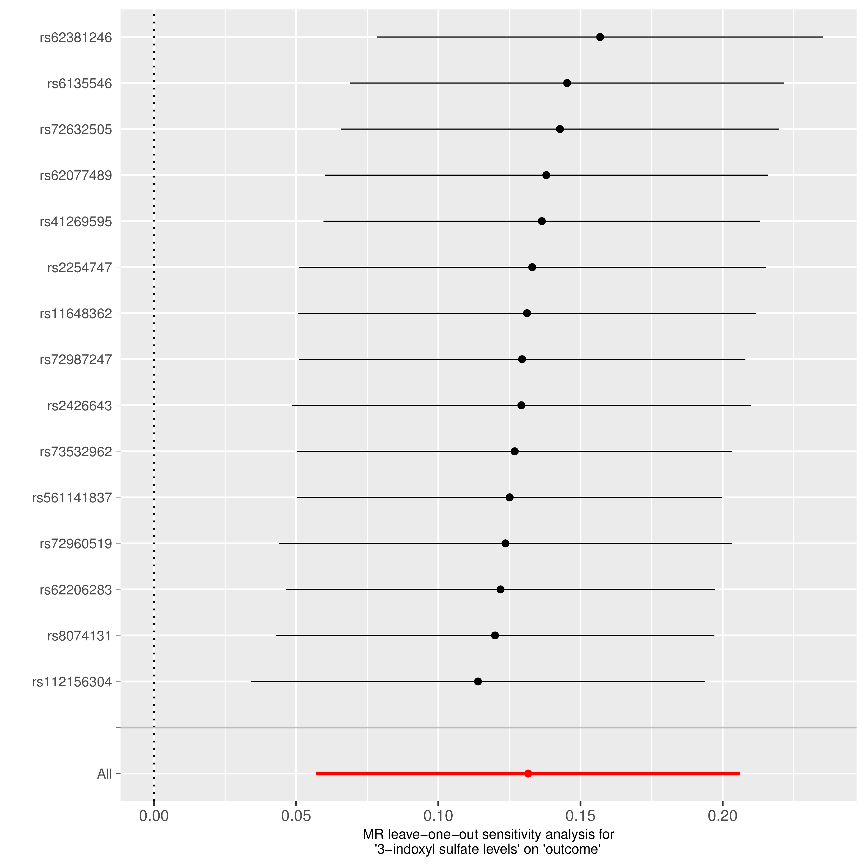** | **B** | **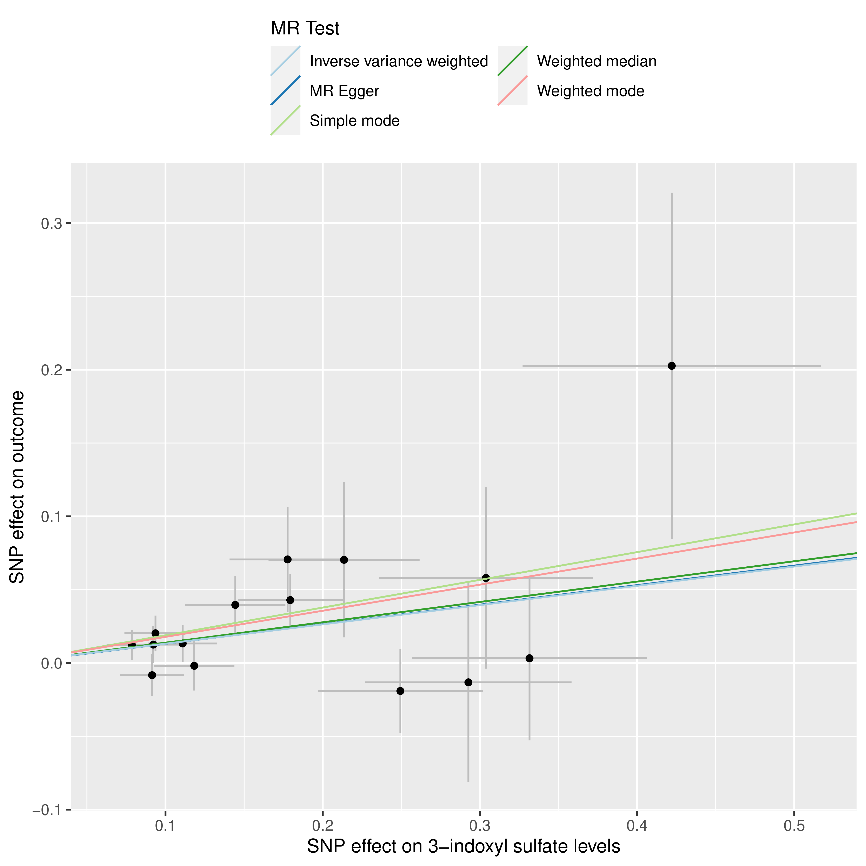** |
| --- | --- | --- | --- |
| **C** | **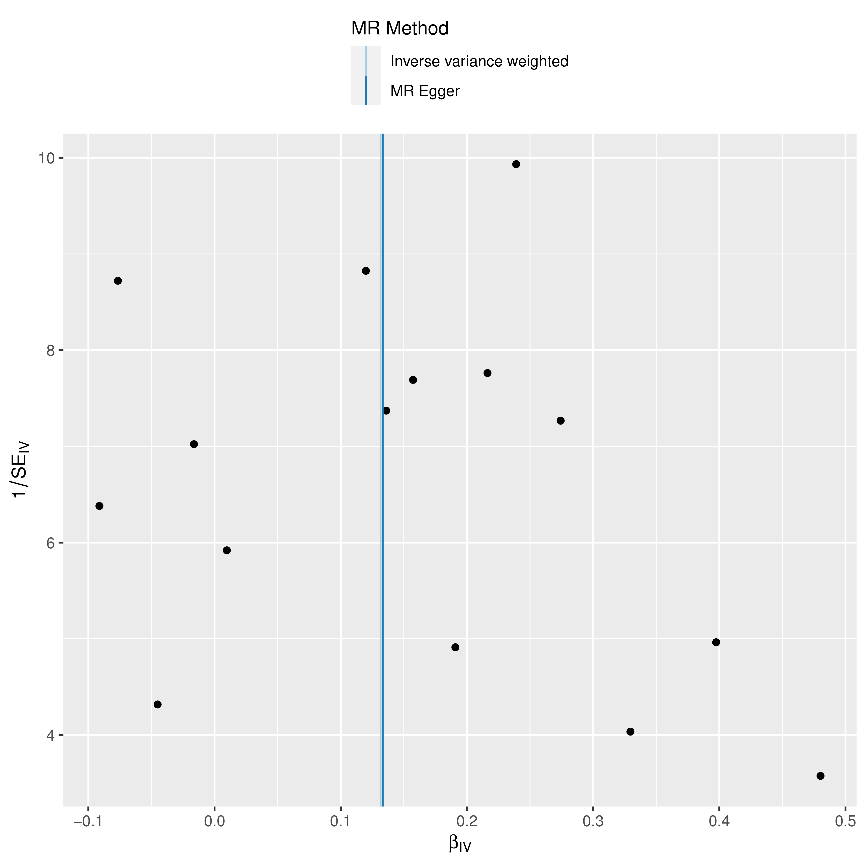** | **D** | **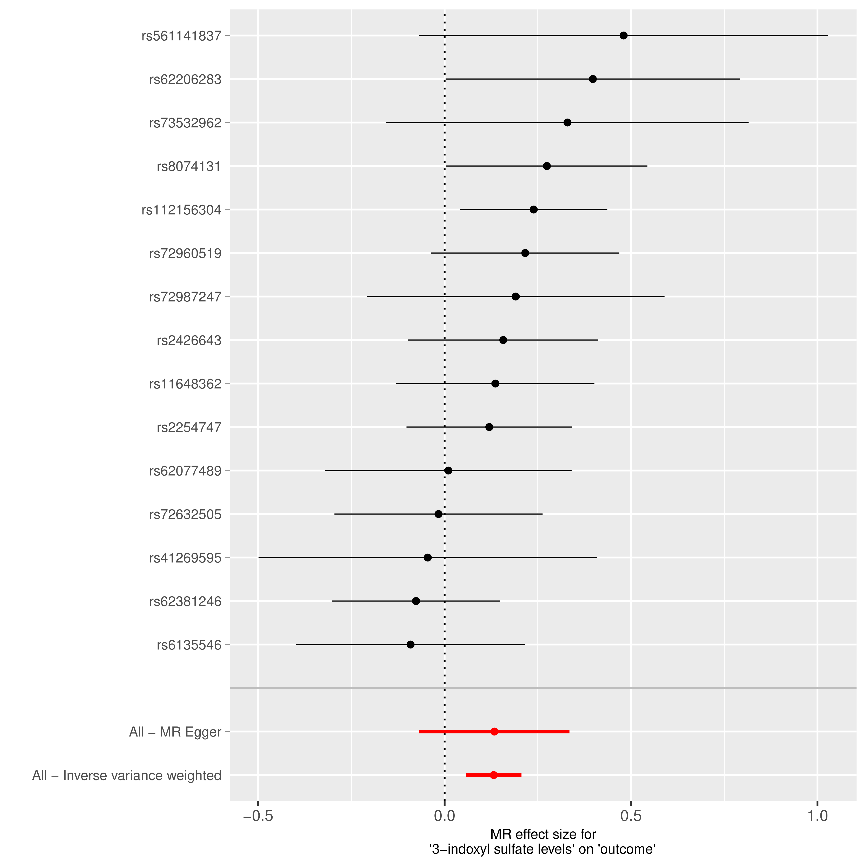** |

**Supplementary Figure 10: Analyses of the Association Between 3-indoxyl sulfate Levels and Venous Thromboembolism Risk.** (A) Leave-One-Out Analysis: Assessment of the influence of individual SNPs associated with 3-indoxyl sulfate levels on Venous Thromboembolism risk. (B) Scatter Plot: Visualization of the causal relationship between individual SNPs within 3-indoxyl sulfate levels and Venous Thromboembolism risk. (C) Funnel Plot: Examination of the overall heterogeneity in MR estimates for the impact of 3-indoxyl sulfate levels on Venous Thromboembolism risk. (D) Forest Plot: Evaluation of the causal effects of individual SNPs on Venous Thromboembolism risk. MR, Mendelian Randomization; SNP, Single Nucleotide Polymorphism.

| **A** | **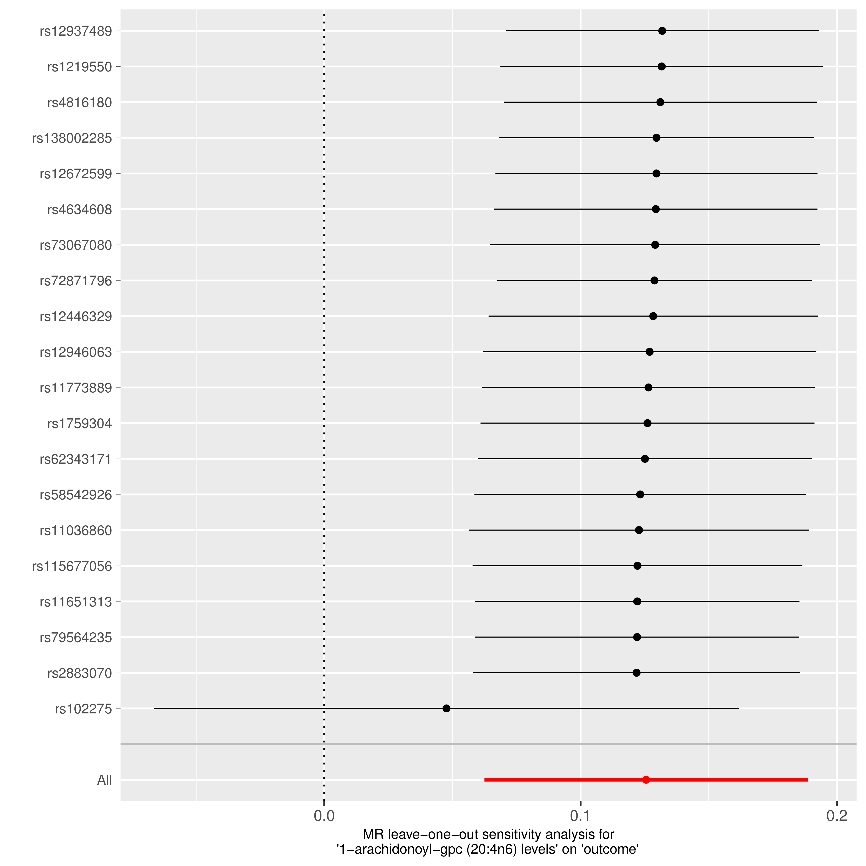** | **B** | **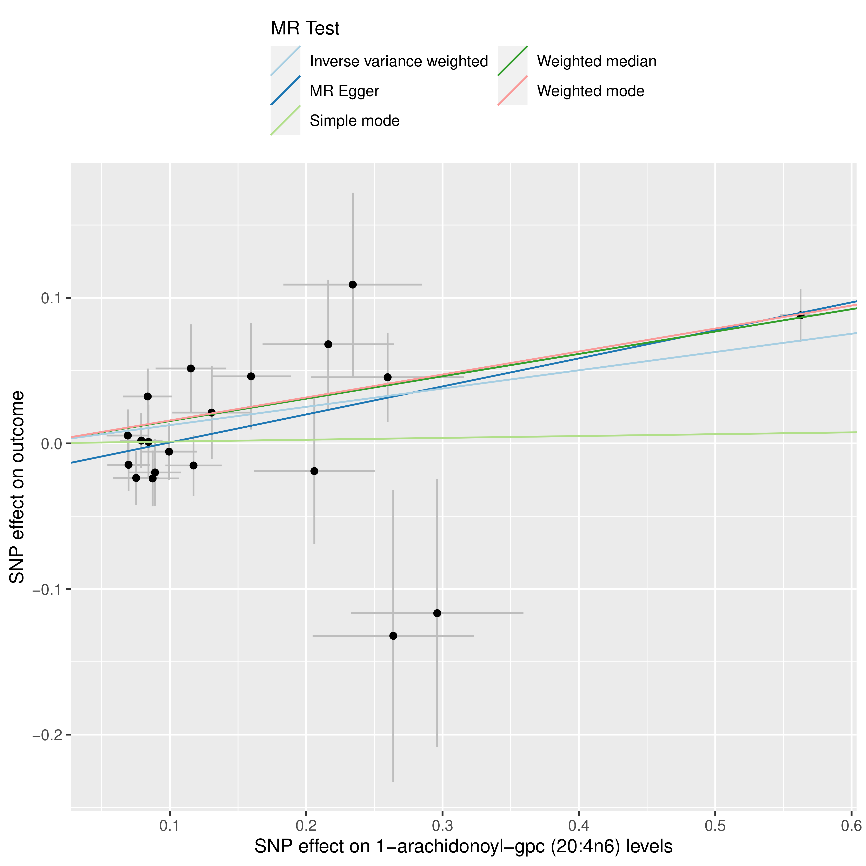** |
| --- | --- | --- | --- |
| **C** | **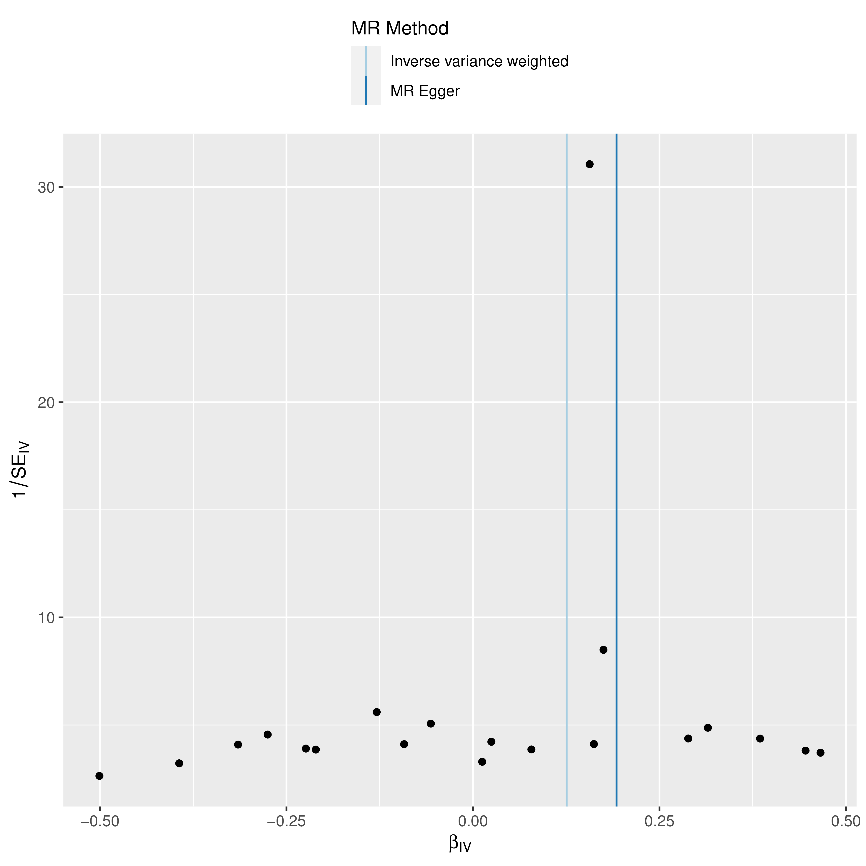** | **D** | **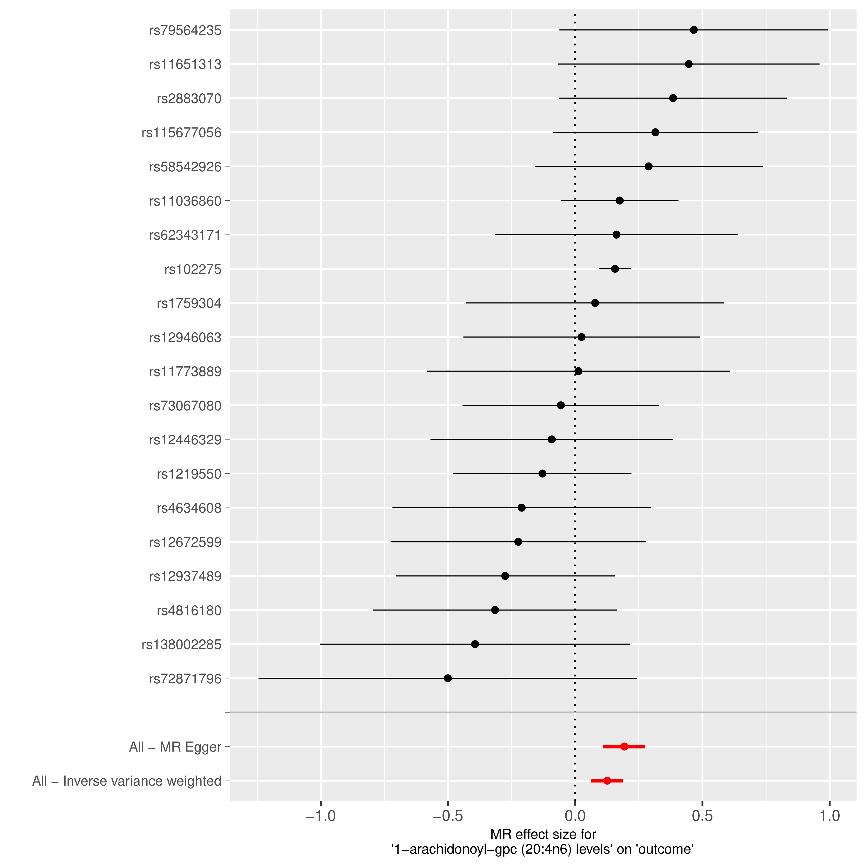** |

**Supplementary Figure 11: Analyses of the Association Between 1-arachidonoyl-gpc (20:4n6) Levels and Deep Vein Thrombosis Risk.** (A) Leave-One-Out Analysis: Assessment of the influence of individual SNPs associated with 1-arachidonoyl-gpc (20:4n6) levels on Deep Vein Thrombosis risk. (B) Scatter Plot: Visualization of the causal relationship between individual SNPs within 1-arachidonoyl-gpc (20:4n6) levels and Deep Vein Thrombosis risk. (C) Funnel Plot: Examination of the overall heterogeneity in MR estimates for the impact of 1-arachidonoyl-gpc (20:4n6) levels on Deep Vein Thrombosis risk. (D) Forest Plot: Evaluation of the causal effects of individual SNPs on Deep Vein Thrombosis risk. MR, Mendelian Randomization; SNP, Single Nucleotide Polymorphism.

| **A** | **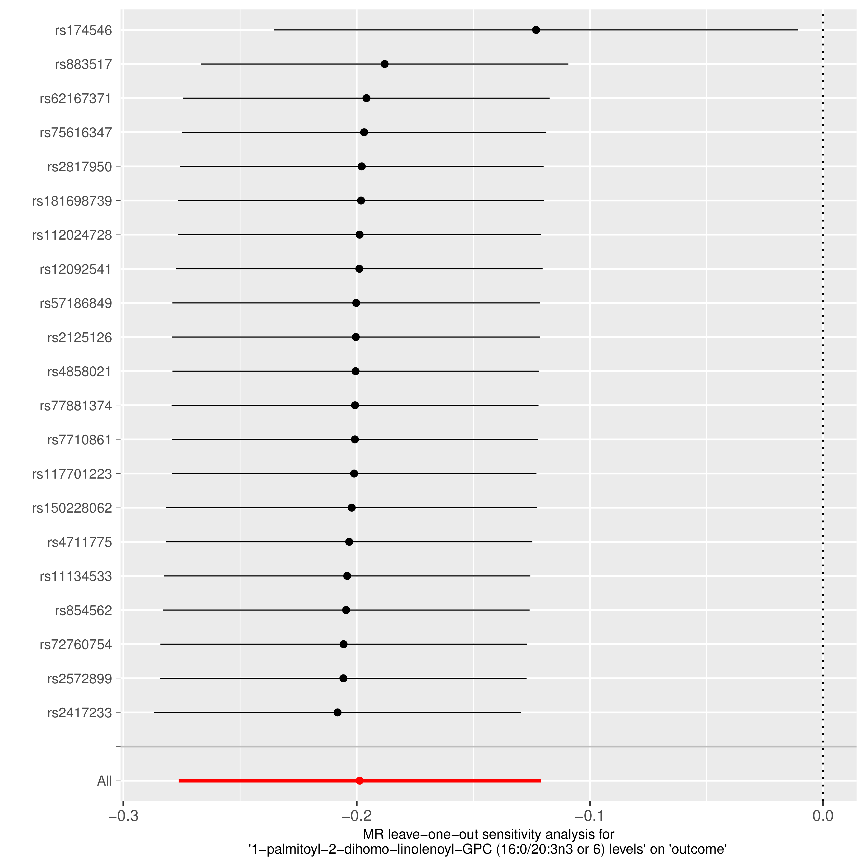** | **B** | **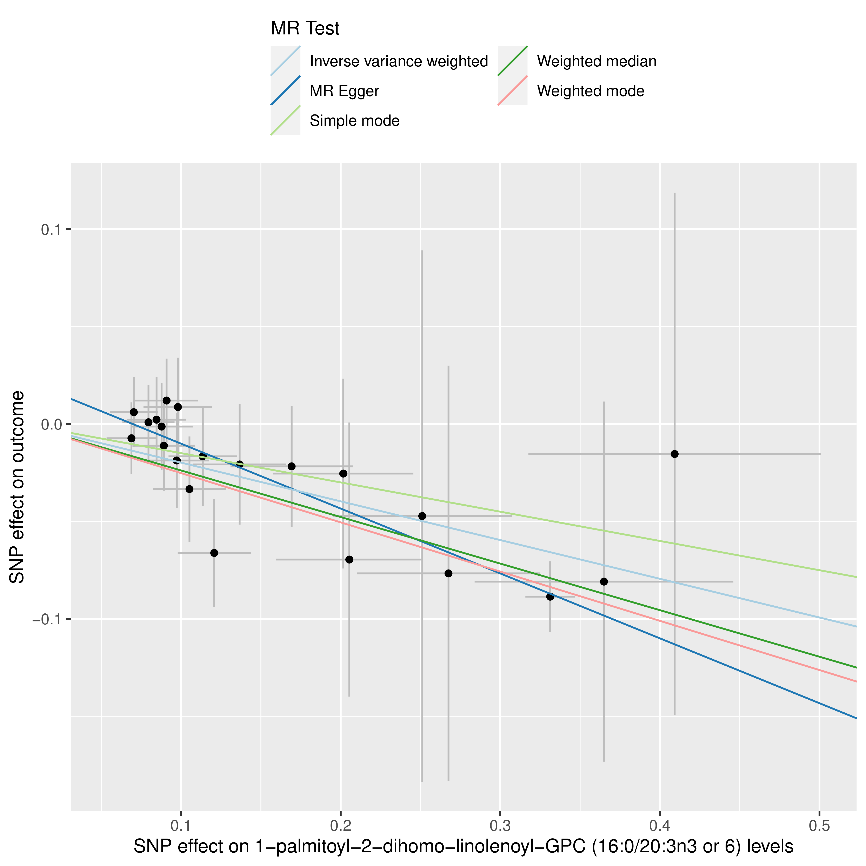** |
| --- | --- | --- | --- |
| **C** | **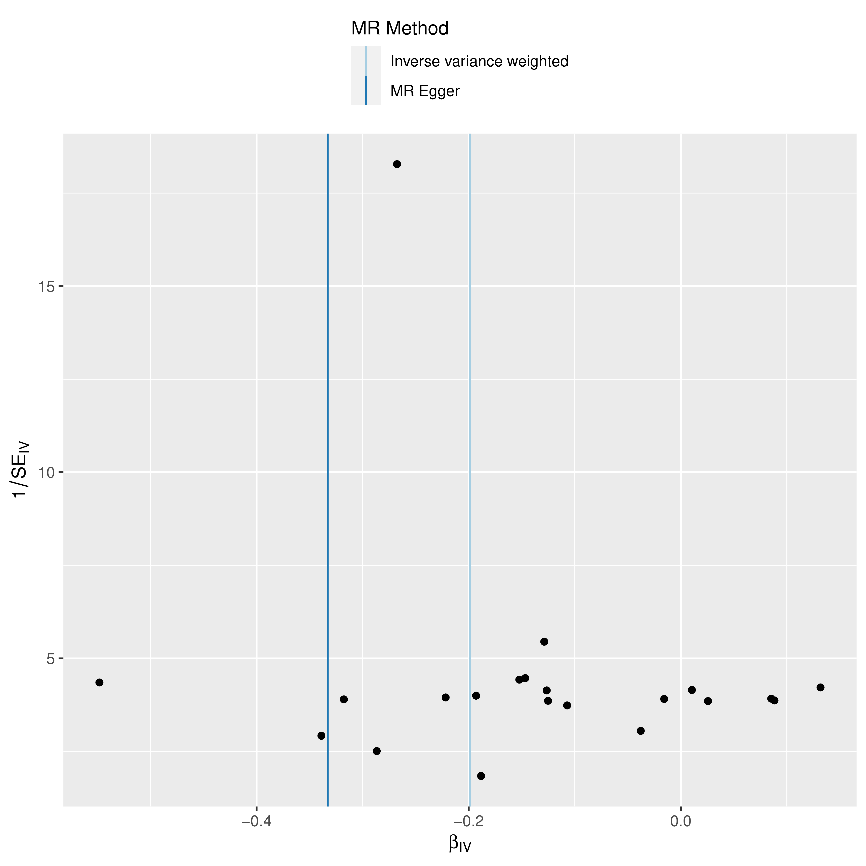** | **D** | **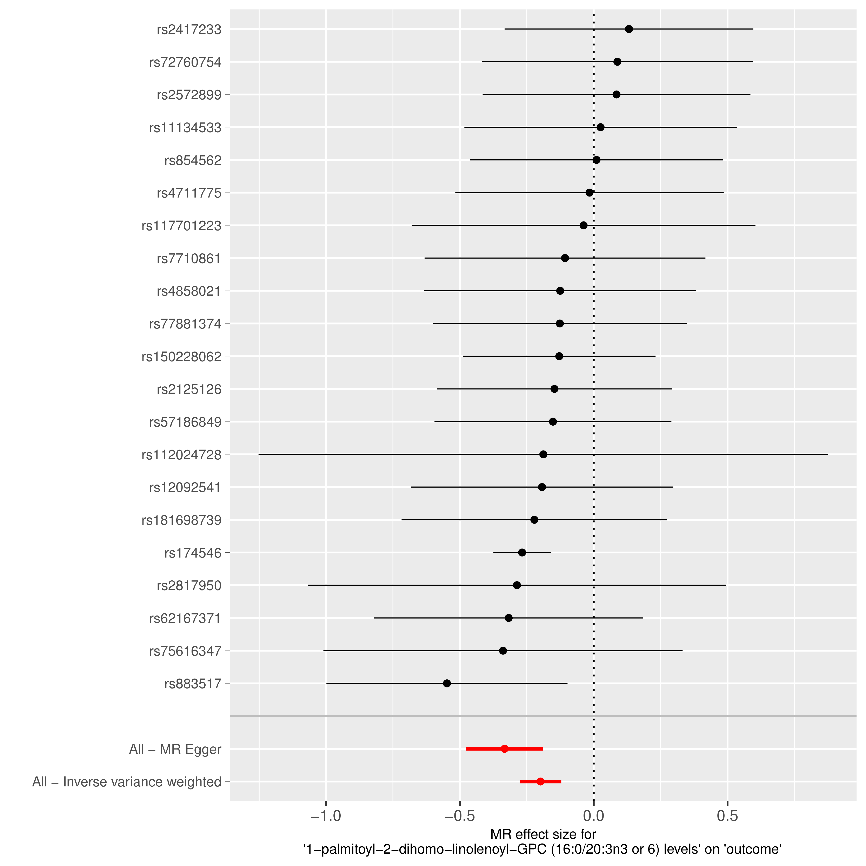** |

**Supplementary Figure 12: Analyses of the Association Between 1-palmitoyl-2-dihomo-linolenoyl-GPC (16:0/20:3n3 or 6) Levels and Deep Vein Thrombosis Risk.** (A) Leave-One-Out Analysis: Assessment of the influence of individual SNPs associated with **1-**palmitoyl-2-dihomo-linolenoyl-GPC (16:0/20:3n3 or 6) levels on Deep Vein Thrombosis risk. (B) Scatter Plot: Visualization of the causal relationship between individual SNPs within 1-palmitoyl-2-dihomo-linolenoyl-GPC (16:0/20:3n3 or 6) levels and Deep Vein Thrombosis risk. (C) Funnel Plot: Examination of the overall heterogeneity in MR estimates for the impact of 1-palmitoyl-2-dihomo-linolenoyl-GPC (16:0/20:3n3 or 6) levels on Deep Vein Thrombosis risk. (D) Forest Plot: Evaluation of the causal effects of individual SNPs on Deep Vein Thrombosis risk. MR, Mendelian Randomization; SNP, Single Nucleotide Polymorphism.

| **A** | **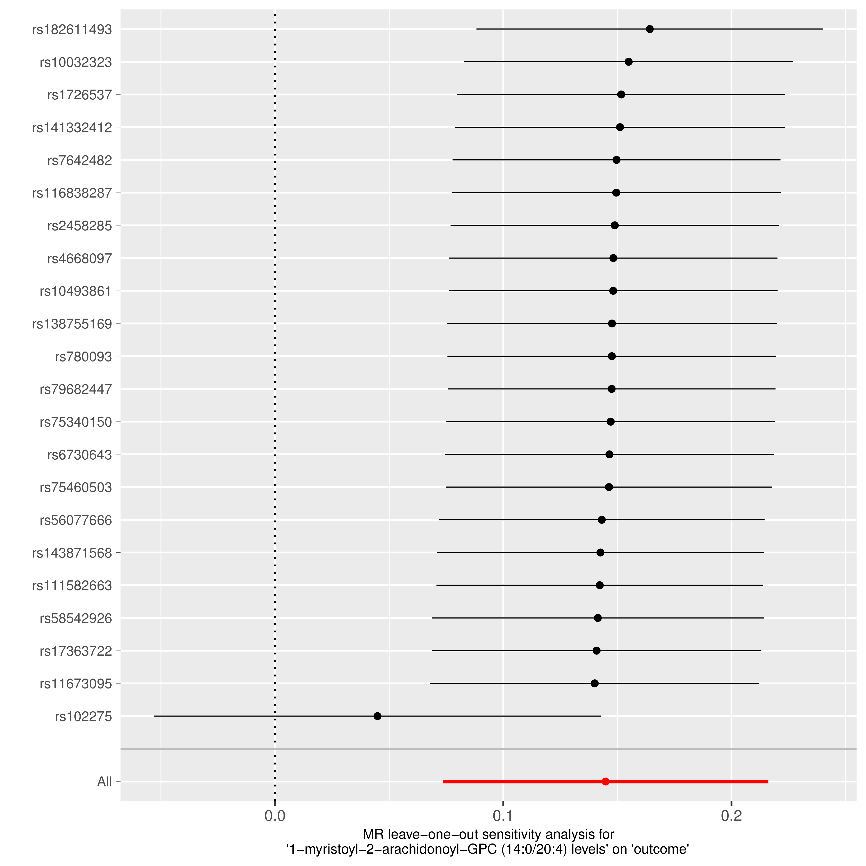** | **B** | **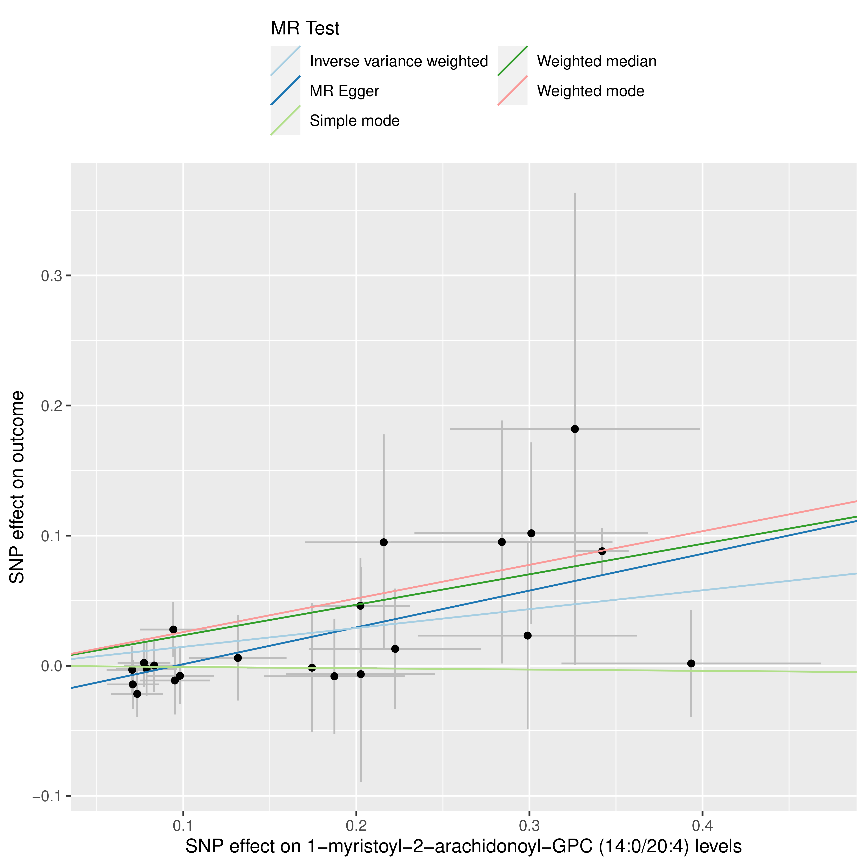** |
| --- | --- | --- | --- |
| **C** | **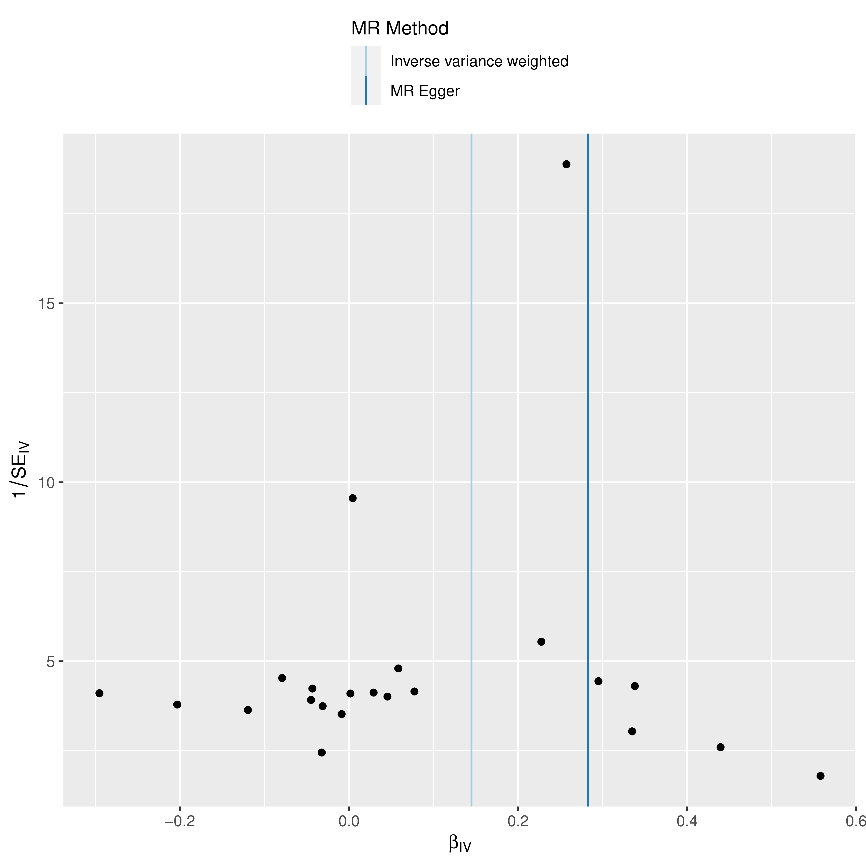** | **D** | **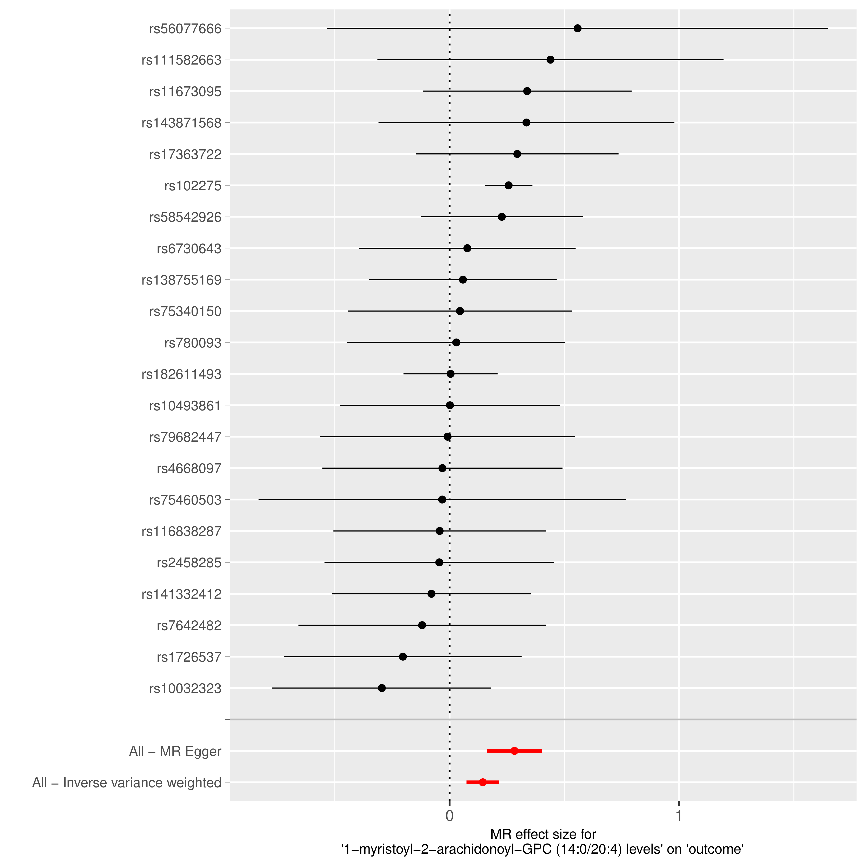** |

**Supplementary Figure 13: Analyses of the Association Between 1-myristoyl-2-arachidonoyl-GPC (14:0/20:4) Levels and Deep Vein Thrombosis Risk.** (A) Leave-One-Out Analysis: Assessment of the influence of individual SNPs associated with 1-myristoyl-2-arachidonoyl-GPC (14:0/20:4) levels on Deep Vein Thrombosis risk. (B) Scatter Plot: Visualization of the causal relationship between individual SNPs within 1-myristoyl-2-arachidonoyl-GPC (14:0/20:4) levels and Deep Vein Thrombosis risk. (C) Funnel Plot: Examination of the overall heterogeneity in MR estimates for the impact of 1-myristoyl-2-arachidonoyl-GPC (14:0/20:4) levels on Deep Vein Thrombosis risk. (D) Forest Plot: Evaluation of the causal effects of individual SNPs on Deep Vein Thrombosis risk. MR, Mendelian Randomization; SNP, Single Nucleotide Polymorphism.

| **A** | **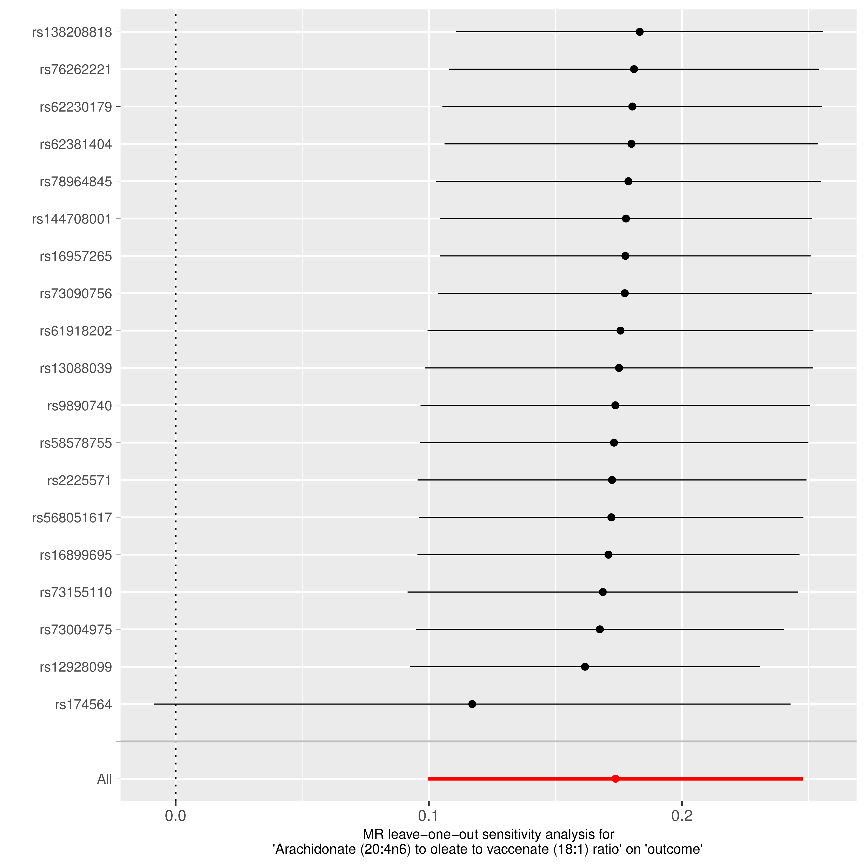** | **B** | **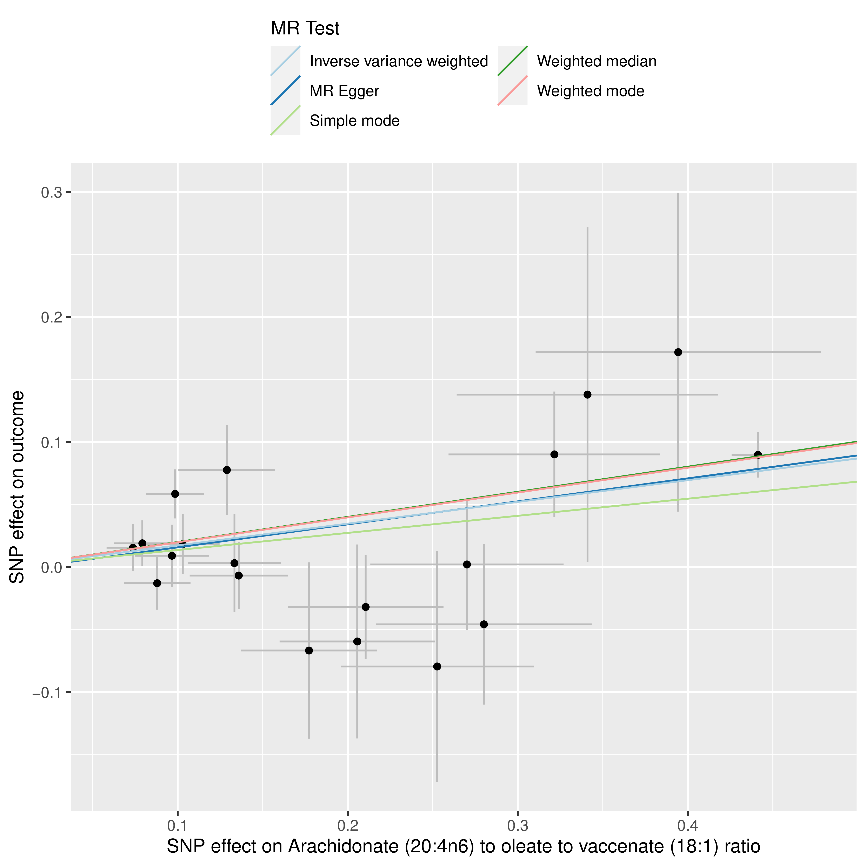** |
| --- | --- | --- | --- |
| **C** | **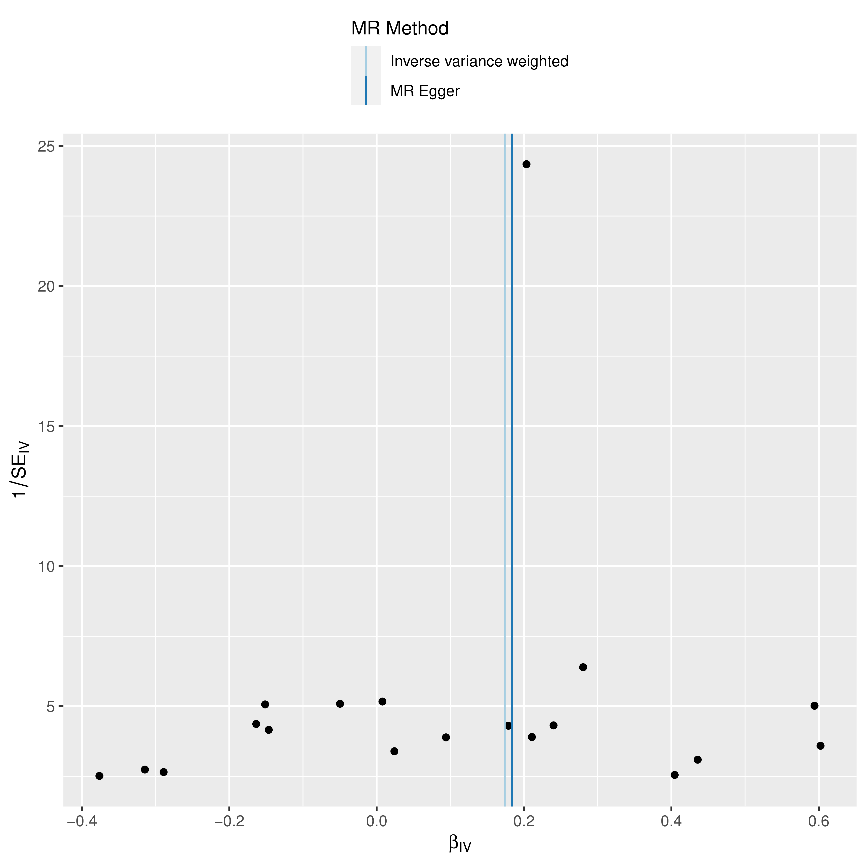** | **D** | **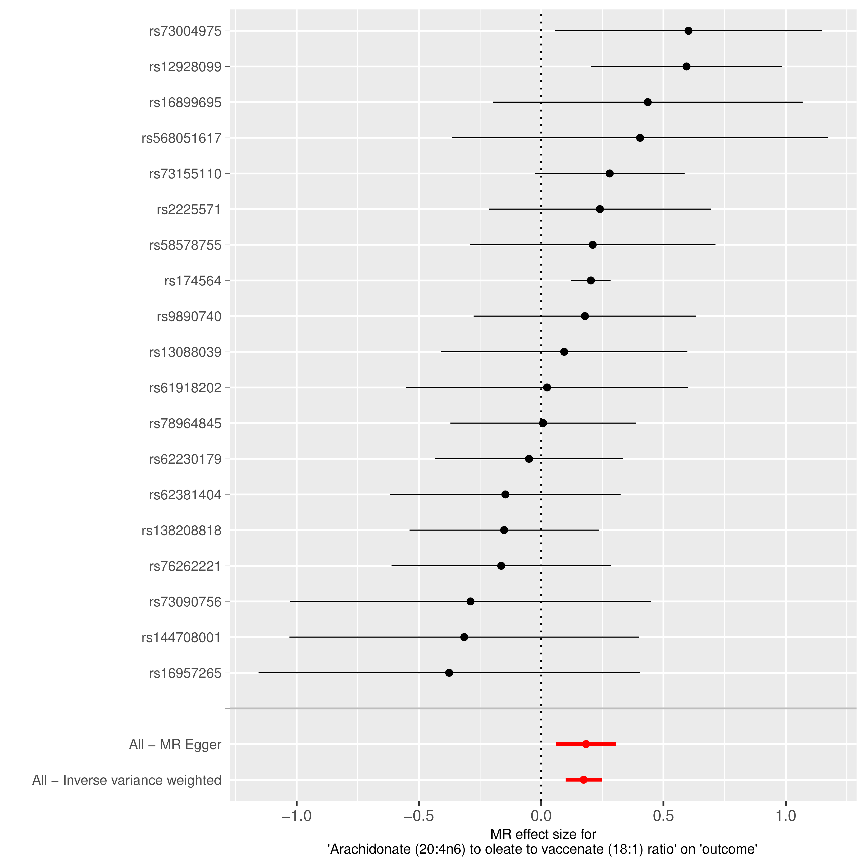** |

**Supplementary Figure 14: Analyses of the Association Between Arachidonate (20:4n6) to oleate to vaccenate (18:1) ratio and Deep Vein Thrombosis Risk.** (A) Leave-One-Out Analysis: Assessment of the influence of individual SNPs associated with Arachidonate (20:4n6) to oleate to vaccenate (18:1) ratio on Deep Vein Thrombosis risk. (B) Scatter Plot: Visualization of the causal relationship between individual SNPs within Arachidonate (20:4n6) to oleate to vaccenate (18:1) ratio and Deep Vein Thrombosis risk. (C) Funnel Plot: Examination of the overall heterogeneity in MR estimates for the impact of Arachidonate (20:4n6) to oleate to vaccenate (18:1) ratio on Deep Vein Thrombosis risk. (D) Forest Plot: Evaluation of the causal effects of individual SNPs on Deep Vein Thrombosis risk. MR, Mendelian Randomization; SNP, Single Nucleotide Polymorphism.

| **A** | **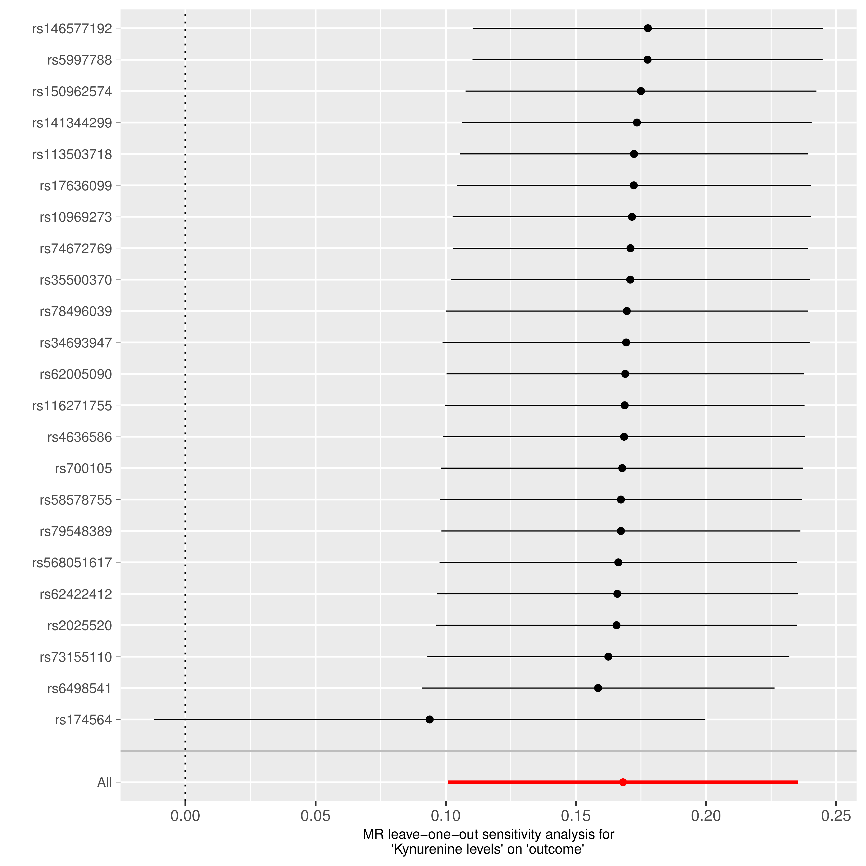** | **B** | **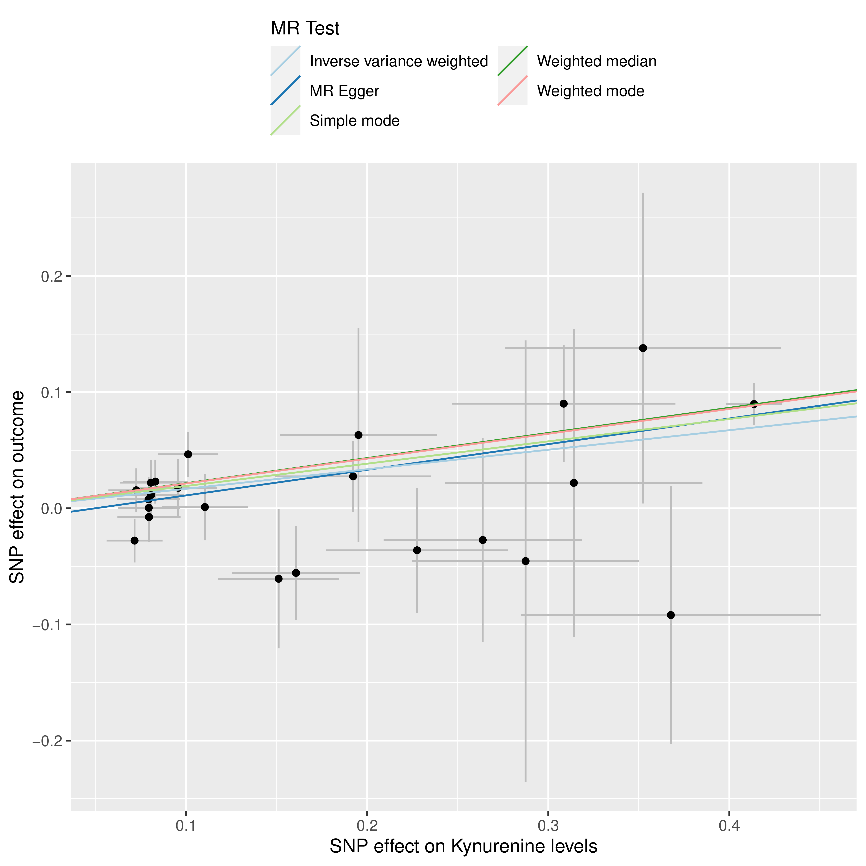** |
| --- | --- | --- | --- |
| **C** | **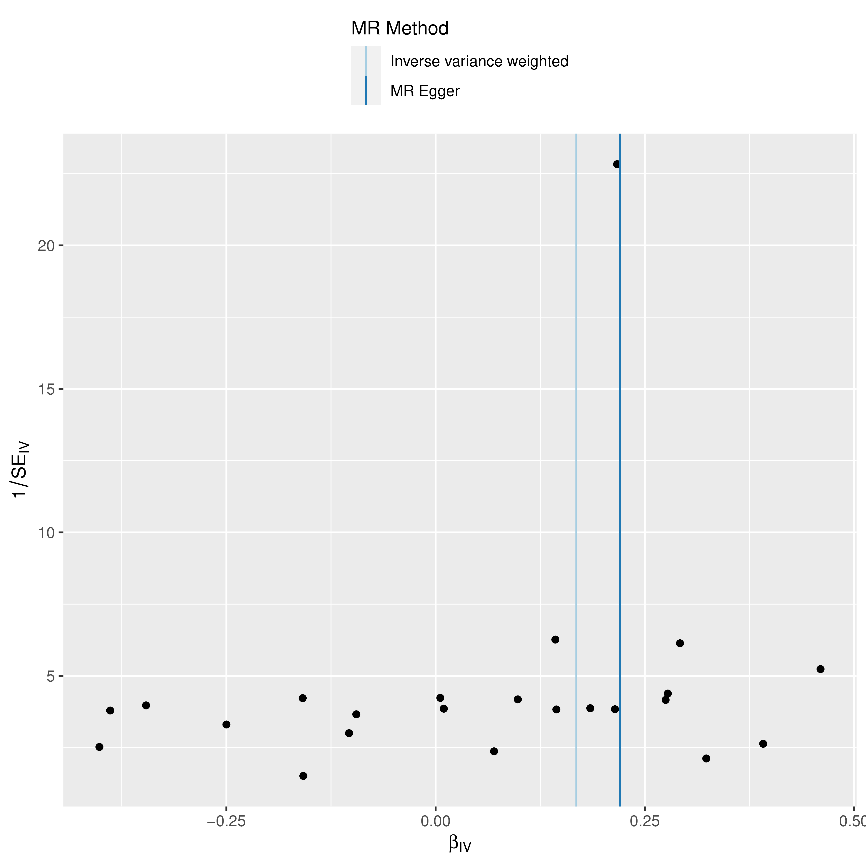** | **D** | **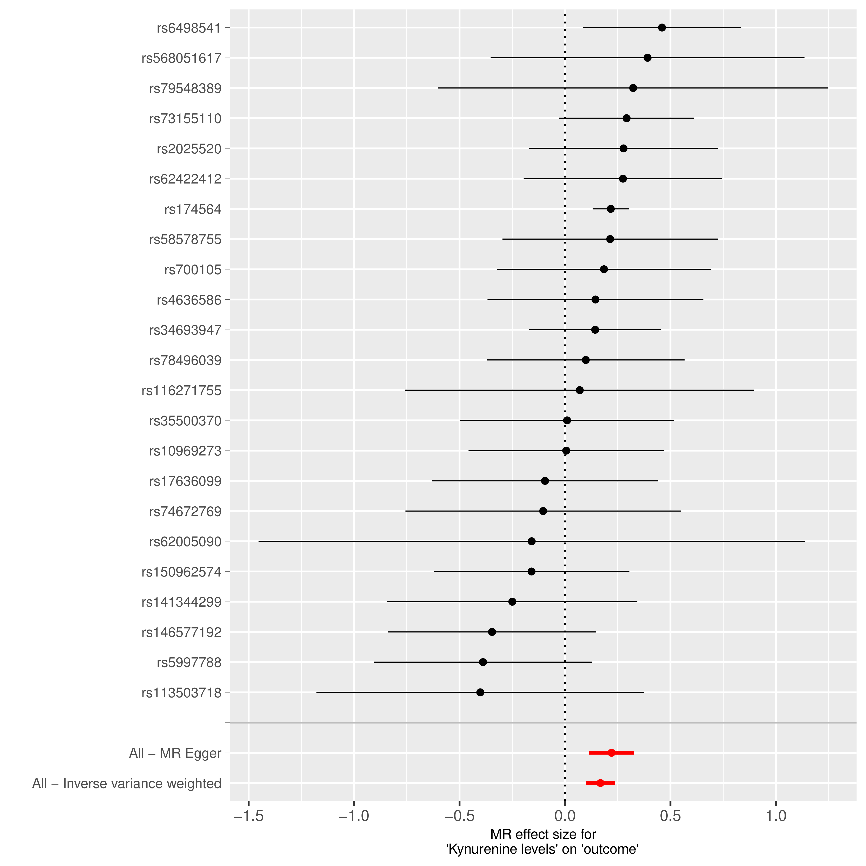** |

**Supplementary Figure 15: Analyses of the Association Between Kynurenine Levels and Deep Vein Thrombosis Risk.** (A) Leave-One-Out Analysis: Assessment of the influence of individual SNPs associated with Kynurenine levels on Deep Vein Thrombosis risk. (B) Scatter Plot: Visualization of the causal relationship between individual SNPs within Kynurenine levels and Deep Vein Thrombosis risk. (C) Funnel Plot: Examination of the overall heterogeneity in MR estimates for the impact of Kynurenine levels on Deep Vein Thrombosis risk. (D) Forest Plot: Evaluation of the causal effects of individual SNPs on Deep Vein Thrombosis risk. MR, Mendelian Randomization; SNP, Single Nucleotide Polymorphism.

| **A** | 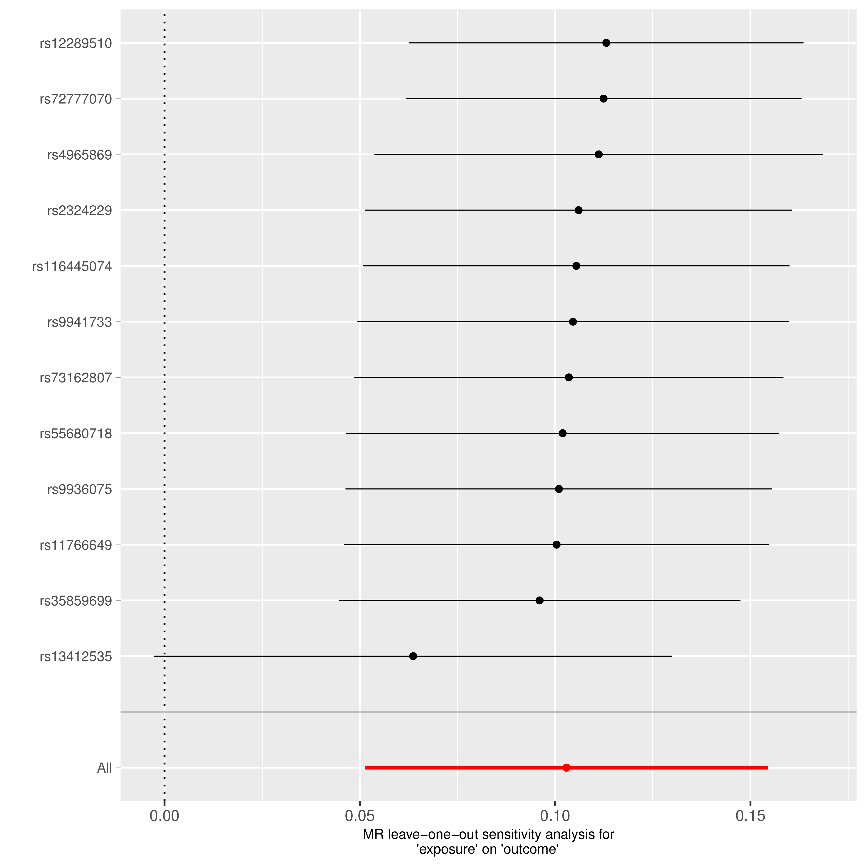 | **B** | 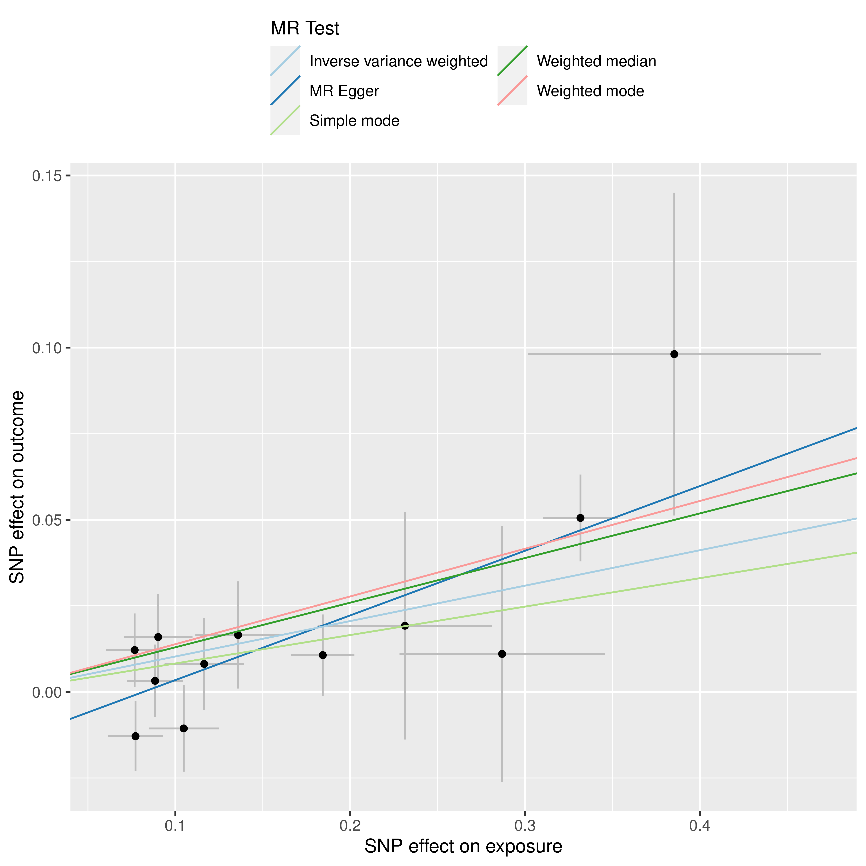 |
| --- | --- | --- | --- |
| **C** | 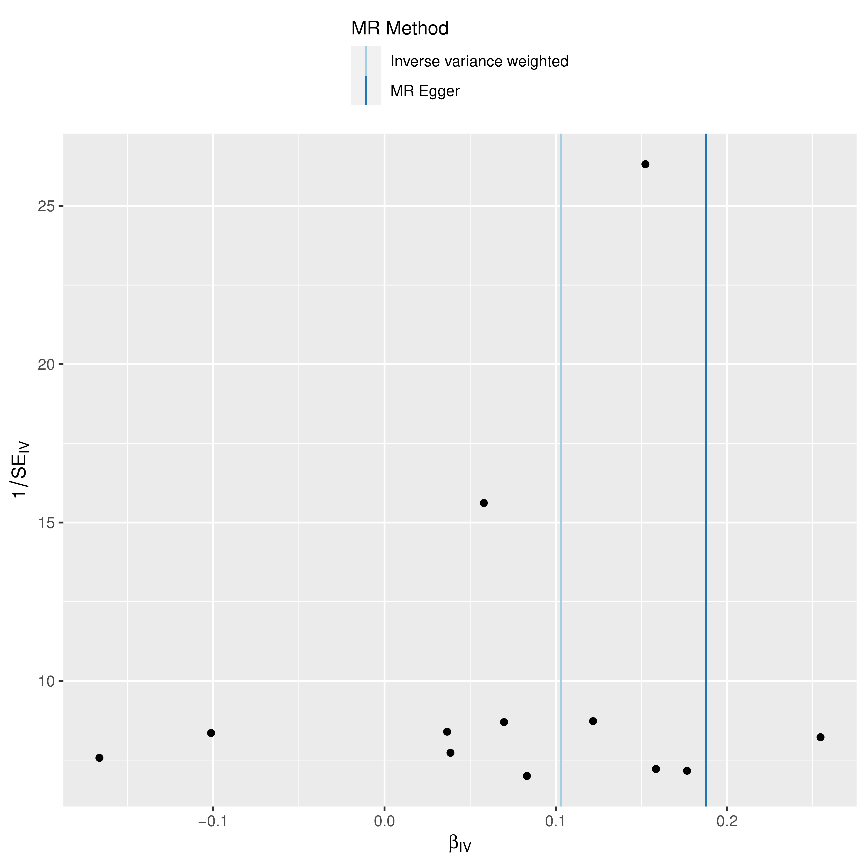 | **D** | 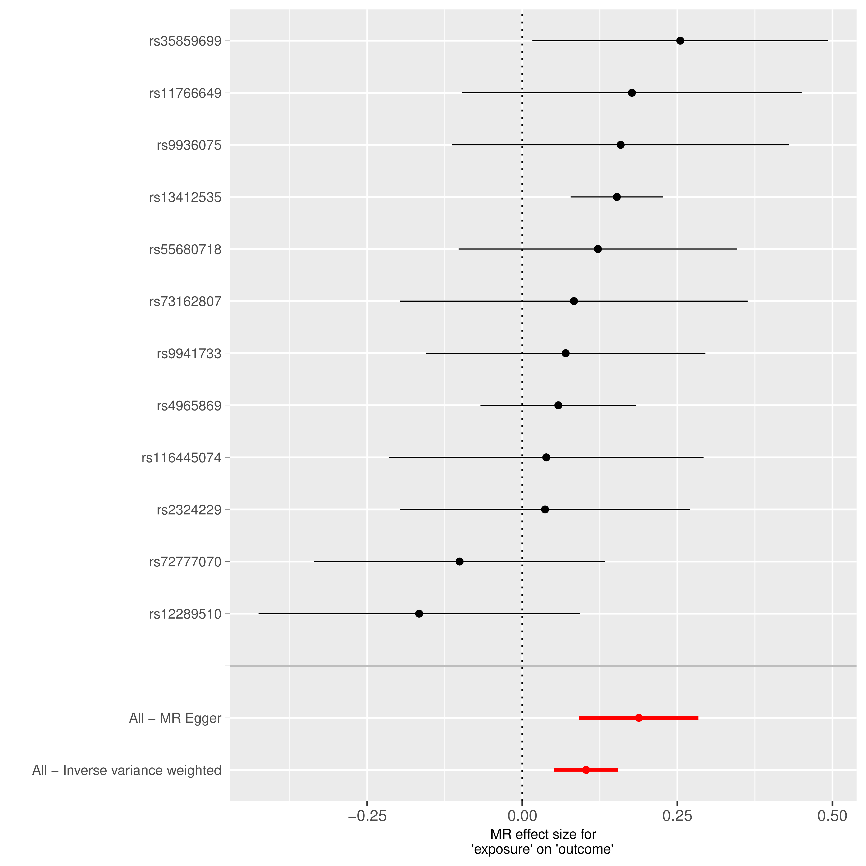 |

**Supplementary Figure 16: Analyses of the Association Between PDGF-BB and Venous Thromboembolism Risk.** (A) Leave-One-Out Analysis: Assessment of the influence of individual SNPs associated with PDGF-BB on Venous Thromboembolism risk. (B) Scatter Plot: Visualization of the causal relationship between individual SNPs within PDGF-BB and Venous Thromboembolism risk. (C) Funnel Plot: Examination of the overall heterogeneity in MR estimates for the impact of PDGF-BB on Venous Thromboembolism risk. (D) Forest Plot: Evaluation of the causal effects of individual SNPs on Venous Thromboembolism risk. PDGF-BB, Platelet-Derived Growth Factor bb; MR, Mendelian Randomization; SNP, Single Nucleotide Polymorphism.

| **A** | **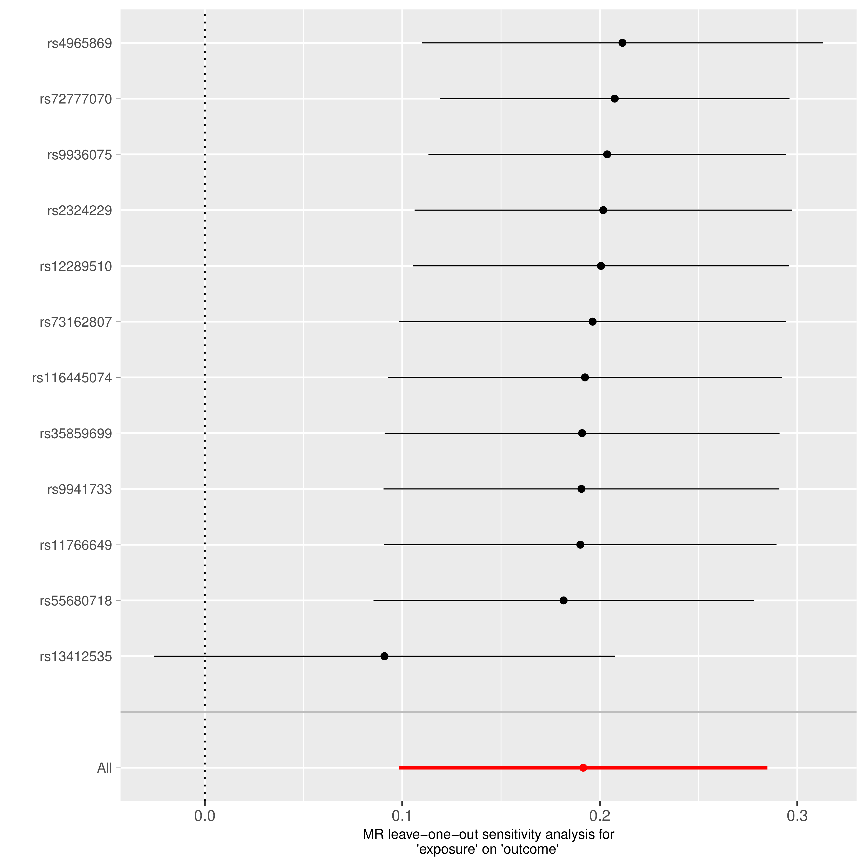** | **B** | **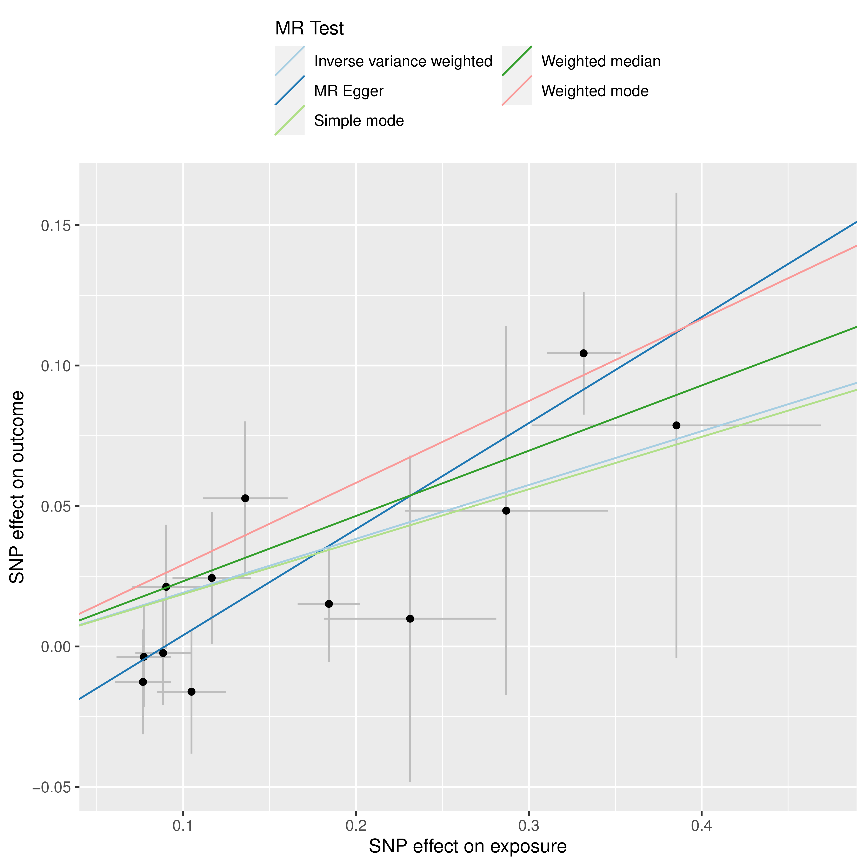** |
| --- | --- | --- | --- |
| **C** | **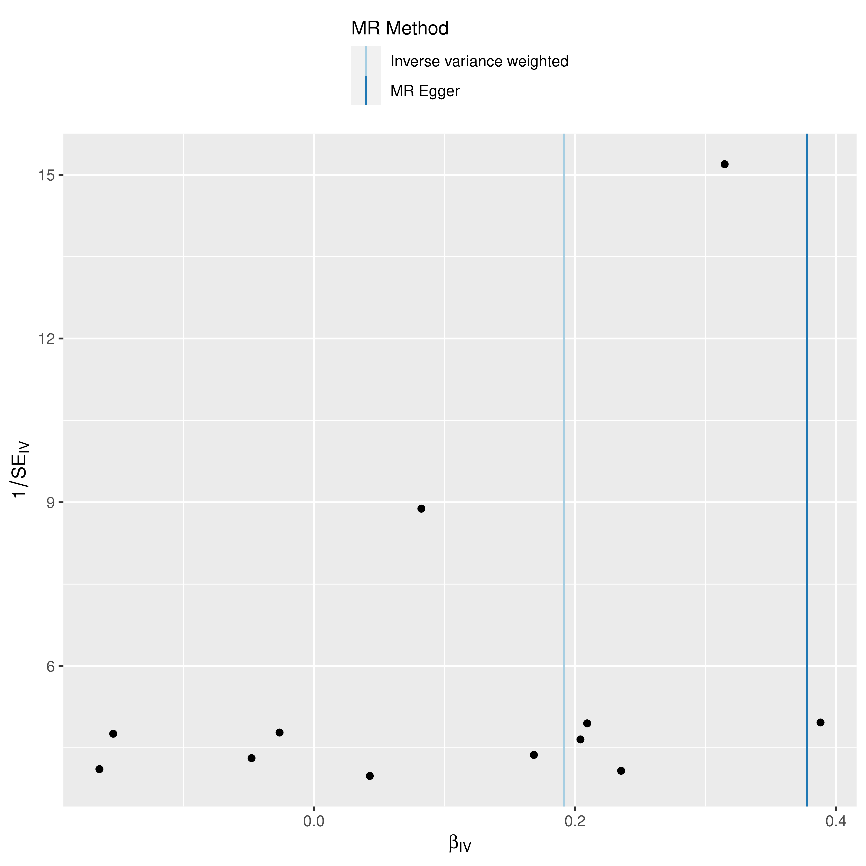^[[1]](#endnote-1)^** | **D** | **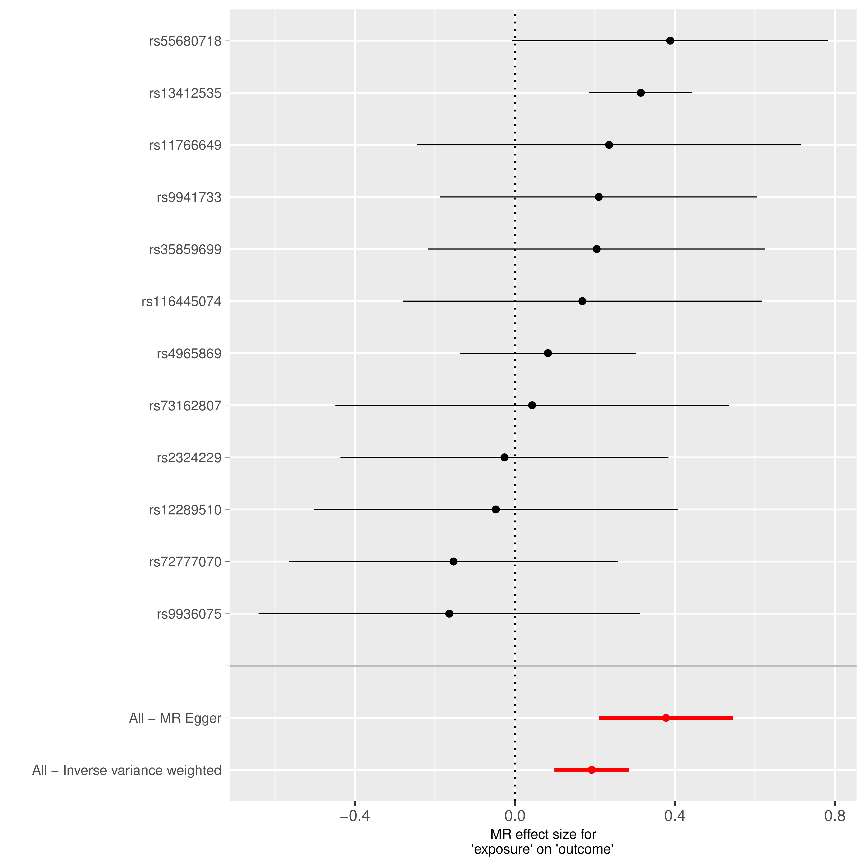** |

**Supplementary Figure 17: Analyses of the Association Between PDGF-BB and Deep Vein Thrombosis Risk.** (A) Leave-One-Out Analysis: Assessment of the influence of individual SNPs associated with PDGF-BB on Deep Vein Thrombosis risk. (B) Scatter Plot: Visualization of the causal relationship between individual SNPs within PDGF-BB and Deep Vein Thrombosis risk. (C) Funnel Plot: Examination of the overall heterogeneity in MR estimates for the impact of PDGF-BB on Deep Vein Thrombosis risk. (D) Forest Plot: Evaluation of the causal effects of individual SNPs on Deep Vein Thrombosis risk. PDGF-BB, Platelet-Derived Growth Factor bb; MR, Mendelian Randomization; SNP, Single Nucleotide Polymorphism.

| **A** | **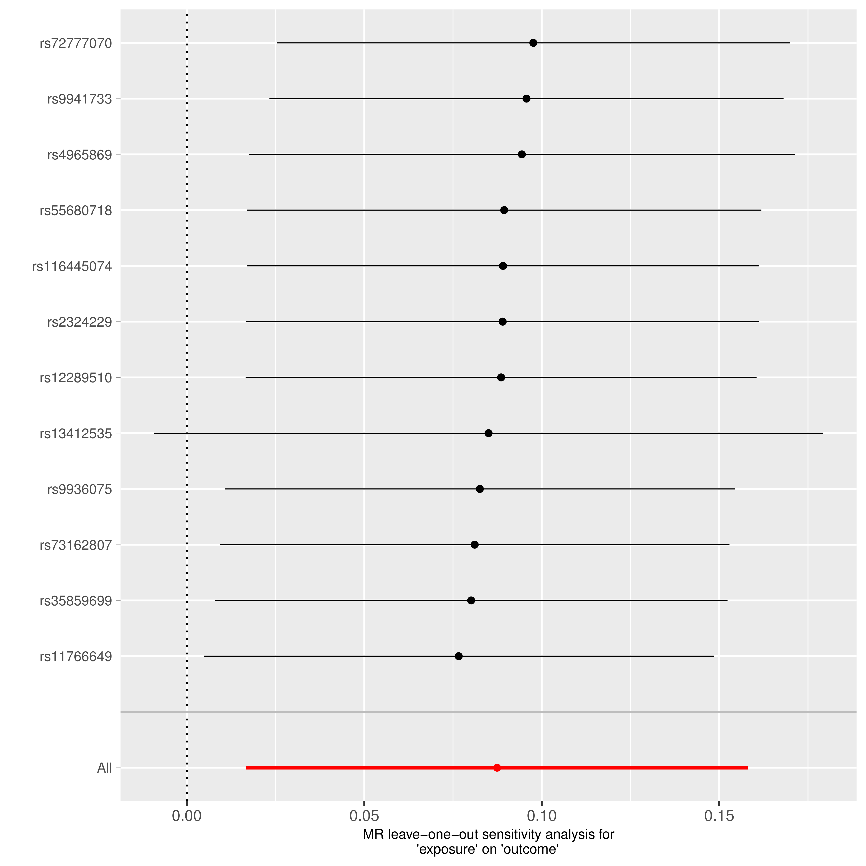** | **B** | **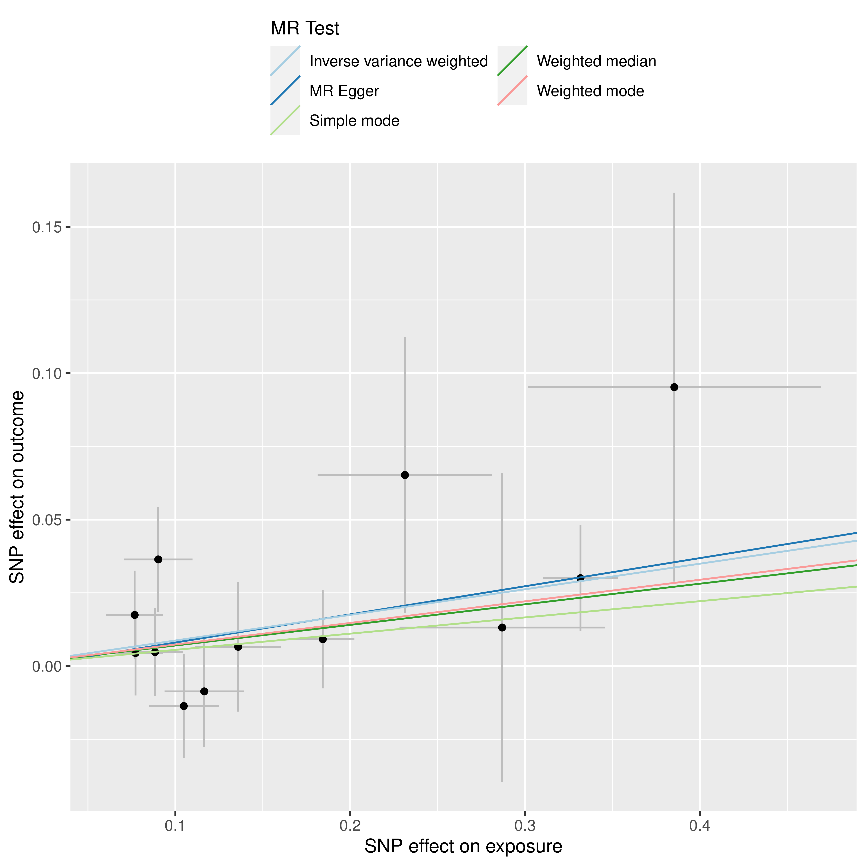** |
| --- | --- | --- | --- |
| **C** | **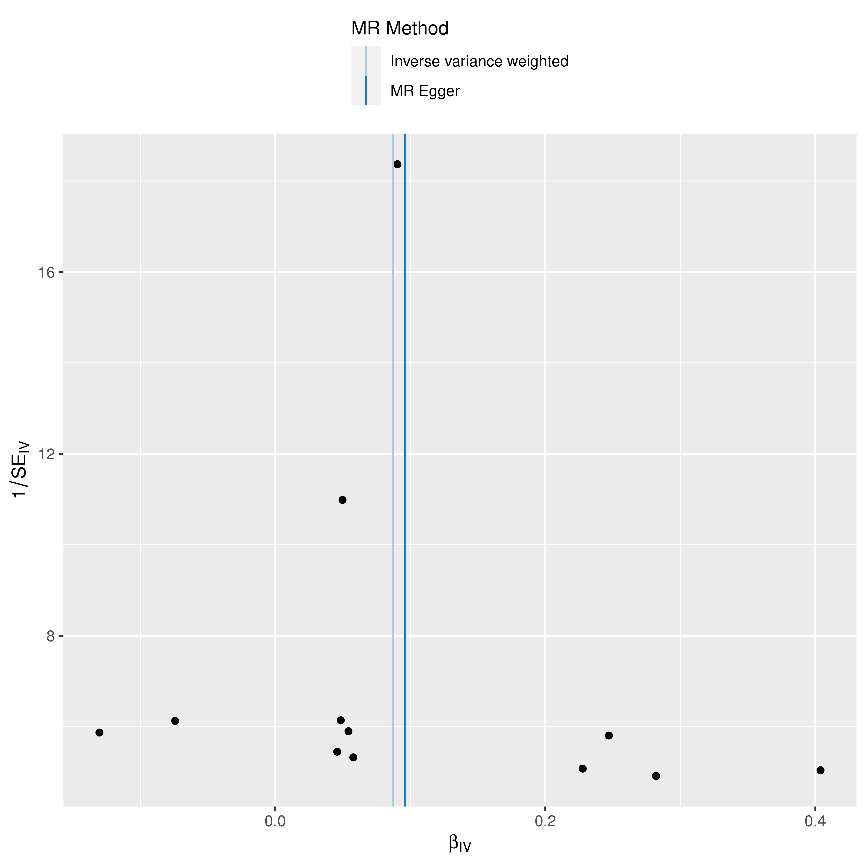** | **D** | **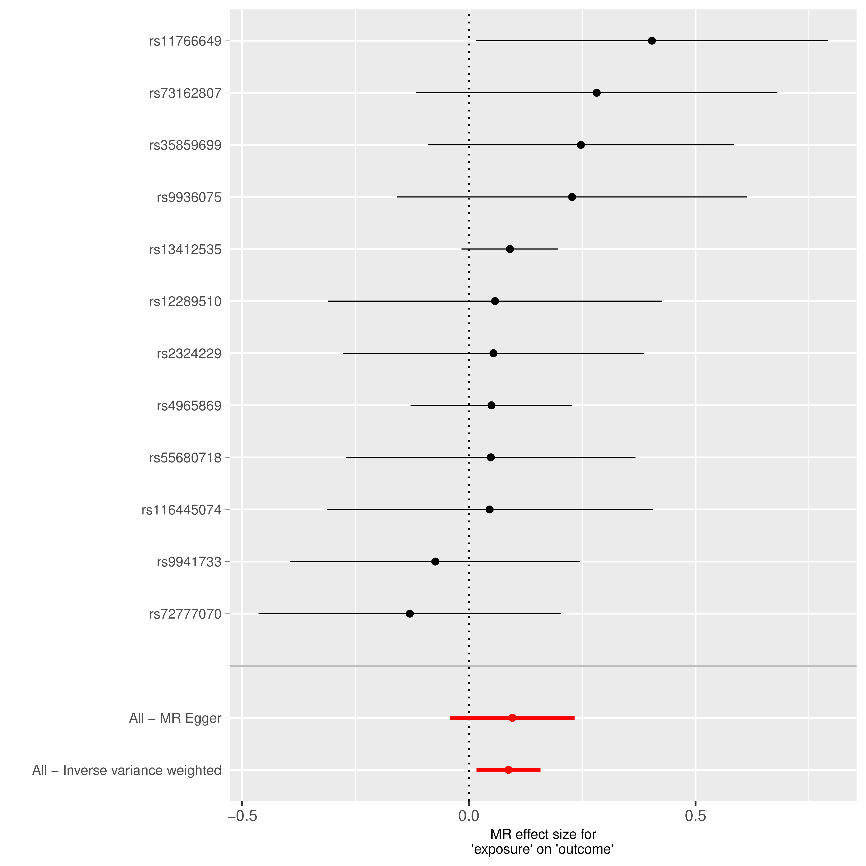** |

**Supplementary Figure 18: Analyses of the Association Between PDGF-BB and Pulmonary Embolism Risk.** (A) Leave-One-Out Analysis: Assessment of the influence of individual SNPs associated with PDGF-BB on Pulmonary Embolism risk. (B) Scatter Plot: Visualization of the causal relationship between individual SNPs within PDGF-BB and Pulmonary Embolism risk. (C) Funnel Plot: Examination of the overall heterogeneity in MR estimates for the impact of PDGF-BB on Pulmonary Embolism risk. (D) Forest Plot: Evaluation of the causal effects of individual SNPs on Pulmonary Embolism risk. PDGF-BB, Platelet-Derived Growth Factor bb; MR, Mendelian Randomization; SNP, Single Nucleotide Polymorphism.

1. [↑](#endnote-ref-1)
